# Phytochemical analysis and anticancer effect of *Camellia oleifera* bud ethanol extract in non-small cell lung cancer A549 cells

**DOI:** 10.3389/fphar.2024.1359632

**Published:** 2024-03-28

**Authors:** Jingming Niu, Xiaoyan Jia, Nian Yang, Yuanquan Ran, Xia Wu, Furong Ding, Dongxin Tang, Minyi Tian

**Affiliations:** ^1^ First Affiliated Hospital of Guizhou University of Traditional Chinese Medicine, Guiyang, China; ^2^ National and Local Joint Engineering Research Center for the Exploitation of Homology Resources of Southwest Medicine and Food, Guizhou University, Guiyang, China; ^3^ College of Life Sciences, Guizhou University, Guiyang, China

**Keywords:** *Camellia oleifera*, non-small cell lung cancer, UHPLC-Q-Orbitrap-MS, anti-proliferation, apoptosis, metastasis

## Abstract

*Camellia oleifera* is a medicine food homology plant widely cultivated in the Yangtze River Basin and southern China due to its camellia oil. *Camellia oleifera* bud and fruit exist simultaneously, and its bud is largely discarded as waste. However, *C. oleifera* bud has been used in traditional Chinese medicine to treat a variety of ailments. Thus, the purpose of this study was to identify the chemical components of *C. oleifera* bud ethanol extract (EE) and first evaluate its anticancer effects in non-small cell lung cancer A549 cells. Based on UHPLC-Q-Orbitrap-MS analysis, seventy components were identified. For anticancer activity, *C. oleifera* bud EE had remarkable cytotoxic effect on non-small cell lung cancer A549 (IC_50_: 57.53 ± 1.54 μg/mL) and NCI-H1299 (IC_50_: 131.67 ± 4.32 μg/mL) cells, while showed lower cytotoxicity on non-cancerous MRC-5 (IC_50_ > 320 μg/mL) and L929 (IC_50_: 179.84 ± 1.08 μg/mL) cells. It dramatically inhibited the proliferation of A549 cells by inducing cell cycle arrest at the G1 phase. Additionally, it induced apoptosis in A549 cells through a mitochondria-mediated pathway, which decreased mitochondrial membrane potential, upregulated Bax, activated caspase 9 and caspase 3, and resulted in PARP cleavage. Wound healing and transwell invasion assays demonstrated that *C. oleifera* bud EE inhibited the migration and invasion of A549 cells in a dose-dependent manner. The above findings indicated that *C. oleifera* bud EE revealed notable anticancer effects by inhibiting proliferation, inducing apoptosis, and suppressing migration and invasion of A549 cells. Hence, *C. oleifera* bud ethanol extract could serve as a new source of natural anticancer drugs.

## 1 Introduction

Cancer is the second leading cause of death, with lung cancer contributing to 18% of all cancer deaths (Sung et al., 2021). The cell types of lung cancer are categorized as small cell lung cancer (SCLC) and non-small cell lung cancer (NSCLC), in which NSCLC accounts for 85% of all lung cancer cases (Gupta et al., 2022). Natural products are a crucial source of anticancer drugs (Newman and Cragg, 2012). Numerous clinically used anticancer drugs are derived from natural products, such as vinblastine, vincristine, paclitaxel, etc. (van Der Heijden et al., 2004; Newman and Cragg, 2012; Naeem et al., 2022).

*Camellia oleifera* Abel., a medicine food homology plant belonging to the genus *Camellia* (Theaceae family), is widely distributed in the Yangtze River Basin and southern China and is an important woody oil crop to prepare camellia oil (Min and Bartholomew, 2007; Li X. et al., 2014; WFO, 2023). Camellia oil is extracted from *Camellia oleifera* seed and listed in the Pharmacopoeia of the People’s Republic of China as an injection solvent and ointment base (China Pharmacopoeia Committee, 2020). Besides, camellia oil is commonly used as cooking oil in China and is usually rich in unsaturated fatty acids, saturated fatty acids, and bioactive substances like polyphenols, flavonoids, phytosterol, squalene, and vitamin E (Li et al., 2022). Previous studies displayed that camellia oil and its bioactive substances had numerous pharmacological effects, such as modulating gastrointestinal microbiota, alleviating liver damage, regulating blood lipid levels, and possessing anticancer, anti-asthmatic, anti-diabetic, antibacterial, antioxidant, and anti-inflammatory properties (Li et al., 2022). *Camellia oleifera* seed cake is a defatted seed meal, a by-product of extracting oil from the seeds, and is used for animal feeding and burning for heating (Xiao et al., 2017). Besides, *C. oleifera* seed and seed cake are used in traditional Chinese medicine for the treatment of diarrhea, abdominal pain, constipation, pruritus, eczema, and scald (Chinese Materia Medica Editorial Committee, 1999). Previous studies have shown that seed and seed cake contain a variety of components like triterpenoid saponins, flavonoids, and polyphenols, which have numerous pharmacological activities, such as anticancer, hypoglycemic, antioxidant, anti-inflammatory, antibacterial, and anti-melanogenesis effects (Zong et al., 2015; Di et al., 2017; Luan et al., 2020; Zhang et al., 2020). *Camellia oleifera* fruit shell can be utilized as a natural colorant (Nakpathom et al., 2017), a skin-whitening agent (Liu et al., 2019), and in the preparation of activated carbon (Zhang et al., 2012). *Camellia oleifera* root, as a traditional Chinese medicine, is used to treat stomachache, pharyngitis, toothache, bruises, and burns (Chinese Materia Medica Editorial Committee, 1999). Various active ingredients have been identified in *C. oleifera* fruit shell and root and have been demonstrated to possess antitumor effects (Luan et al., 2020).

The time between flower bud differentiation and fruit growth of *C. oleifera* overlaps, and its bud and fruit exist at the same time (Wen et al., 2021; Ren et al., 2023). When *C. oleifera* fruit is harvested, flower bud is present in abundance and is discarded as waste. As a traditional Chinese medicine, *C. oleifera* bud has the effect of blood cooling and hemostasis and is utilized to treat vomiting blood, hematochezia, and scald (Chinese Materia Medica Editorial Committee, 1999; Sugimoto et al., 2009). Past studies have revealed that *C. oleifera* flower bud contains polysaccharides, phenolics, flavonoids, and procyanidins as well as possesses antioxidant and gastroprotective effects (Feng et al., 2022; Xiang et al., 2022; Chen et al., 2023).

Past studies primarily focus on the camellia oil, seed, seed cake, fruit shell, and root of *C. oleifera* and have confirmed their anticancer activity. *Camellia oleifera* bud has been used in traditional Chinese medicine to treat various ailments. However, little research has been done on the chemical composition and biological activities of *C. oleifera* bud, which may limit its exploitation. Therefore, our current study aims to analyze the chemical composition of *C. oleifera* bud and first explore its anti-tumor effects.

## 2 Materials and methods

### 2.1 Chemical and reagents

Cisplatin was purchased from Aladdin Industrial Corporation (Shanghai, China). AO (acridine orange), EB (ethidium bromide), MTT (3-[4, 5-dimethylth-iazol-2-yl]-2, 5 diphenyl tetrazolium bromide), BCA protein assay kit, hesperetin, kaempferol, and 4% paraformaldehyde solution were from Solarbio Life Sciences (Beijing, China). Crystal violet staining solution, Hoechst 33,258, RIPA lysis buffer, mitochondrial membrane potential assay kit with JC-1, and BeyoECL moon kit were purchased from Beyotime Institute of Biotechnology (Shanghai, China). Antibodies were from Cell Signaling Technology (Danvers, Massachusetts, United States).

### 2.2 Plant material

*Camellia oleifera* was obtained in October 2021 from Yuping County, Tongren District, Guizhou Province, China (latitude: 27°31′29.84″N and longitude: 108°93′20.97″E). The species identification of *C. oleifera* was confirmed by Prof. Guoxiong Hu from the College of Life Sciences, Guizhou University. The voucher specimen (herbarium code: CO20211027) was deposited at the National and Local Joint Engineering Research Center for the Exploitation of Homology Resources of Southwest Medicine and Food, Guizhou University.

### 2.3 Preparation of *Camellia oleifera* bud EE

Fresh buds (500 g) were crushed, placed in a round-bottomed flask, and extracted with ethanol (70%, 2 L) at reflux for 2 h. Then, we collected the filtrate by suction filtration. The filter residue was extracted again under the same conditions. Subsequently, the two filtrates were combined, evaporated under reduced pressure in a rotary evaporator, and then freeze-dried. The ethanol extract (EE) was preserved in sealed brown glass vials and stored in a desiccator.

### 2.4 Composition analysis of *Camellia oleifera* bud EE

UHPLC-Q-Orbitrap-MS (ultra-high-performance liquid chromatography coupled with quadrupole orbitrap mass spectrometer) was used to analyze the phytochemicals in *C. oleifera* bud EE. Dionex Ultimate 3000 RSLC UHPLC was utilized under the following parameters: Thermo Fisher Hypersil GOLD aQ column (100 mm × 2.1 mm, 1.9 μm), column temperature (40 °C), flow rate (0.3 mL/min), injection volume (5 μL), and mobile phase consisted of 0.1% formic acid acetonitrile (A) and 0.1% formic acid aqueous solution (B). Gradient elution was employed to separate the components in EE as follows: 5% A (0–2 min); 5%–95% A (2–42 min); 95% A (42–47 min); 95%–5% A (47–47.1 min); 5% A (47.1–50 min).

Thermo Fisher Scientific Q-Orbitrap-MS with HESI-II (heated electrospray ionization) was used to collect the MS data. The parameters for HESI-II were as follows: capillary temperature (320°C), vaporizer temperature (350°C), spray voltages (−2.5/+ 3.0 kV), RF lens amplitude (60), and auxiliary, sheath, and sweep gas (10 arb, 35 arb, 0 arb). Full mass/ddMS2 mode was employed, and its specific parameters were as follows: full scan range (*m/z* 100 to 1,500), maximum injection time MS1 (100 ms) and MS2 (50 ms), automatic gain control target values MS1 (1e^6^) and MS2 (2e^5^), resolution MS1 (70,000) and MS2 (17,500), and stepped normalized collision energy (20/40/60 eV). Thermo Fisher Scientific Xcalibur 4.1 was utilized for analyzing mass spectrum data. Chemical components were identified by comparison of MS1 and MS2 fragments with the mzVault database and literature data. The allowable relative mass error is limited to 10 ppm.

### 2.5 Cell culture

Non-small cell lung adenocarcinoma cells (A549), non-small cell lung cancer cells (NCI-H1299), murine fibroblast cells (L929), and fetal lung fibroblast cells (MRC-5) were from Kunming Cell Bank, Chinese Academy of Sciences (Kunming, China). A549, NCI-H1299, and L929 cell lines were maintained in Roswell Park Memorial Institute (RPMI) 1640 medium supplemented with 10% fetal bovine serum (FBS), 2 mM glutamine, 100 U/mL penicillin, and 100 μg/mL streptomycin. MRC-5 cells were maintained in Dulbecco’s modified eagle medium (DMEM). All the cell lines were cultured in a humidified incubator at 37°C and 5% CO_2_.

### 2.6 Cytotoxic activity

Cytotoxic activities of *C. oleifera* bud EE, hesperetin, and kaempferol against A549, NCI-H1299, L929, and MRC-5 cell lines were analyzed by MTT assay. Cisplatin was a positive control drug. *Camellia oleifera* bud EE, hesperetin, and kaempferol were dissolved in DMSO and two-fold serially diluted using a medium (the final DMSO concentration <0.05%). For the experimental group, cell suspensions (8 × 10^3^ cells/well, 80 µL) were seeded into 96-well plates. After 24 h incubation, different concentrations of sample solutions (20 µL) were added, and the final concentrations of samples were 20, 40, 80, 160, and 320 μg/mL. For the negative group, the cell suspension (8 × 10^3^ cells per well, 80 µL) was seeded into 96-well plates and incubated for 24 h, and then 20 µL of medium was added. For the blank group, 80 μL medium was added and incubated for 24 h, and 20 μL medium was added. All three groups were incubated for 48 h. Next, MTT solutions (5 mg/mL, 12 µL/well) were added and incubated for 4 h. Finally, DMSO (150 µL) was added to each well to dissolve the formazan crystals, and the absorbance (Ab) was measured at 490 nm by an i-Mark micro-plate reader (Bio-Rad Laboratories, Inc., Hercules, CA, United States). The cell viability rates were calculated using the following formula:

$$\text{Cell viaibility rate}=\left[\frac{{\text{Ab}}_{\left(\text{experimental}\;\text{group}\right)}{\;‐\;\text{Ab}}_{\left(\text{blank}\;\text{group}\right)}}{{\text{Ab}}_{\left(\text{negative}\;\text{group}\right)}{\;‐\;\text{Ab}}_{\left(\text{blank}\;\text{group}\right)}}\right]\times 100\%$$

### 2.7 Colony formation assay

Colony formation assay was used to evaluate A549 cell proliferation ability. Cells were seeded into six-well plates at a density of 200 cells per well, cultured for 24 h, and treated with different concentrations of *C. oleifera* bud EE (0, 10, 20, 30, and 40 μg/mL) for 24 h. Then, we removed the medium, washed each well, and added fresh medium. After culturing for 7 days, cells were washed twice with PBS, fixed with formaldehyde solution (10%, 700 μL) for 30 min, permeabilized with anhydrous methanol (700 μL) for 20 min, and stained with crystal violet (0.1%, 700 μL) for 15 min. The plates were washed with water, dried at room temperature, and photographed. The colony formation rate was calculated according to the following formula:

$$\text{Clone formation rate}=\frac{\text{The}\;\text{number}\;\text{of}\;\text{clones}}{\text{The}\;\text{number}\;\text{of}\;\text{inoculated}\;\text{cells}}\times 100\%$$

### 2.8 Cell cycle analysis

The cell cycle was assessed according to the instructions in the cell cycle staining kit (Multi Sciences (Lianke) Biotech, Co., Ltd., Hangzhou, China). A549 cells were plated in 6-well plates (4 × 10^5^ cells per well), cultured for 24 h, and then exposed to *C. oleifera* bud EE (0, 10, 20, 40, 80, and 160 μg/mL) for 24 h. The cells were washed with cold PBS and stained with 1 mL DNA staining solution containing 10 μL permeabilization solutions in the dark. After 30 min, the stained cells were detected by flow cytometer (ACEA NovoCyte™, ACEA Biosciences, San Diego, CA, United States).

### 2.9 Cell apoptosis assay

#### 2.9.1 Morphology observation

To investigate the effect of *C. oleifera* bud EE on A549 cell apoptosis, cells (4 × 10^5^ cells per well) were seeded into 6-well plates and incubated for 24 h. The cells were subsequently exposed to fresh mediums containing different concentrations of *C. oleifera* bud EE (0, 20, 40, 80, and 160 μg/mL). After 48 h of incubation, a Leica DMi8 microscope (Leica Microsystems, Germany) was utilized to observe changes in A549 cell morphology.

#### 2.9.2 AO/EB dual staining assay

In the AO/EB dual staining assay, AO (1 mg) was added to PBS (10 mL) and fully dissolved to obtain an AO dye solution (100 μg/mL). EB dye solution (100 μg/mL) was obtained by the same method. The AO/EB mixture was prepared by mixing AO dye solution and EB dye solution in equal volumes (1:1). Cells were seeded into 6-well plates at a density of 4 × 10^5^ cells per well and incubated for 24 h. Subsequently, cells were treated with different concentrations (0, 20, 40, 80, and 160 μg/mL) of *C. oleifera* bud EE. After incubation for 48 h, cells were washed twice with PBS and stained with AO/EB mixture (1 mL) for 5 min in the absence of light. Finally, cells were observed under a fluorescence microscope.

#### 2.9.3 Hoechst 33258 staining assay

A549 cells were subjected to the same treatment described above in the Hoechst 33,258 staining assay (Hong et al., 2022). After discarding the previous medium, 4% paraformaldehyde (500 μL) was added, and cells were fixed for 20 min. Subsequently, cells were washed with PBS and incubated in Hoechst 33,258 staining solution (500 μL) (Beyotime, Shanghai, China) for 5 min. Finally, morphological alterations of the nuclei were observed using fluorescence microscopy.

#### 2.9.4 Annexin V-PE/7-AAD assay

Quantitative measurement of A549 cell apoptosis was performed using an Annexin V-PE/7-AAD apoptosis kit. A549 cells (4 × 10^5^ per well) were inoculated in 6-well plates for 24 h. Then, cells were treated with different concentrations (0, 10, 20, 40, 80, and 160 μg/mL) of *C. oleifera* bud EE for 48 h and washed with precooled PBS. Afterward, cells were resuspended in 1 × binding buffer (500 μL) and stained with 5 μL of Annexin V-PE and 10 μL of 7-AAD for 5 min. Finally, the apoptosis rate was measured using a flow cytometer.

### 2.10 Mitochondrial membrane potential assay

The mitochondrial membrane potential (ΔΨm) was detected by the JC-1 assay. Briefly, A549 cells were plated in 6-well plates at 4 × 10^5^ cells per well and incubated for 24 h. Next, cells were treated with different concentrations (0, 10, 20, 40, 80, and 160 μg/mL) of *C. oleifera* bud EE for 48 h. Subsequently, the supernatants were discarded, and cells were washed with PBS. A mixture of culture medium (1 mL) and JC-1 working solution (1 mL) was added and incubated for 20 min. Next, supernatants were removed, and cells were washed twice with JC-1 staining buffer. Finally, cells were observed under a fluorescence microscope.

### 2.11 Wound healing assay

A549 cells were seeded into 6-well plates at 3 × 10^5^ cells per well and incubated overnight until cells were confluent. Subsequently, cells were scratched with a 200 μL pipette tip, washed twice with PBS, and treated with different concentrations (0, 10, 20, 30, and 40 μg/mL) of *C. oleifera* bud EE solutions (2 mL, 0.5% FBS medium preparation) for 24 h. Finally, migration distance was recorded at 0 and 24 h under a Leica DMi8 microscope. Cell migration ability was assessed using migration rate (%), whose calculation formula was as follows:

$$\text{Migration rate}=\frac{{\text{Wound}\;\text{width}}_{\left(0\;h\right)}{\;‐\text{Wound}\;\text{width}}_{\left(24\;h\right)}}{{\text{Wound}\;\text{width}}_{\left(0\;h\right)}}\times 100\%$$

### 2.12 Transwell invasion assay

Transwell invasion assay was performed according to the instructions of Corning^®^ BioCoat™ Matrigel^®^ Invasion Chamber (Corning, NY, United States). The upper chamber was loaded with A549 cells in 5% FBS medium (250 μL, 4 ×10^5^ cells/mL) and various concentrations of *C. oleifera* bud EE solutions (dissolved in 5% FBS medium, 250 µL). Medium (750 μL, containing 15% FBS and different concentrations of *C. oleifera* bud EE) was injected into the lower chamber. After incubation at 37°C for 48 h, cells in the upper chamber that had not penetrated the membrane were removed with a cotton swab. Subsequently, invasive cells were fixed with 4% paraformaldehyde for 2 min, incubated with anhydrous methanol for 20 min, and stained with 0.1% crystal violet for 15 min. Images were captured by a microscope. The invaded cells per field of view were quantified using ImageJ.

### 2.13 Western blotting analysis

A549 cells (4 × 10^5^ cells/well) were incubated in 6-well plates for 24 h and treated with 0 and 160 μg/mL of *C. oleifera* bud EE for 48 h. Then, cell total proteins were isolated through RIPA lysis buffer, and protein concentrations were determined by a BCA protein assay kit (Solarbio, Beijing, China). Subsequently, proteins were separated by 10% sodium dodecyl sulfate-polyacrylamide gel electrophoresis (SDS-PAGE), transferred to polyvinylidene difluoride (PVDF) membranes, blocked with 5% milk in TBS-T (TBS, 0.1% Tween-20) for 1 h, and blotted with primary antibodies at overnight under 4°C. Next, membranes were washed three times with TBS-T and incubated with HRP-conjugated secondary antibodies for 1 h. Proteins were visualized using a BeyoECL moon kit (Beyotime, Shanghai, China), imaged with a ChemiDoc touch imaging system (Bio-Rad Laboratories, Inc., Hercules, CA, United States), and quantified *via* Image Lab software.

### 2.14 Statistical analysis

Data were expressed as means ± standard deviation (SD). The statistical analysis was performed using SPSS 26.0 software (SPSS, Inc., Chicago, IL, United States). The significance of differences between groups was evaluated using a two-tailed unpaired *t*-test or one-way analysis of variance (ANOVA) with the least significant difference (LSD) for *post hoc* tests (*p < 0.05, **p < 0.01, ***p < 0.001).

## 3 Results

### 3.1 Phytochemical compounds of *Camellia oleifera* bud EE

**FIGURE 1:**
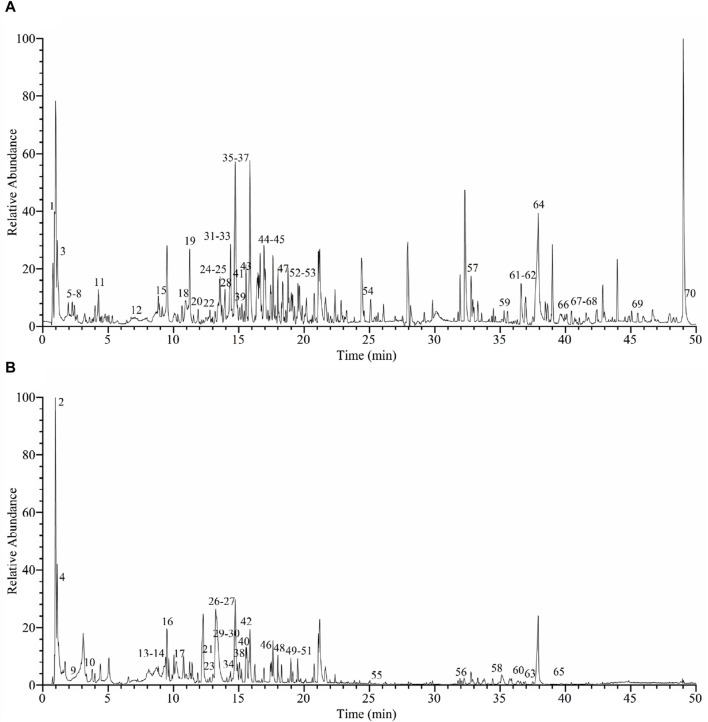
UHPLC-Q-Orbitrap-MS base peak chromatograms of *Camellia oleifera* bud EE in positive ion mode **(A)** and negative ion mode **(B)**.

The yield of EE from *C. oleifera* bud was 3.06%. The chromatogram of *C. oleifera* bud extract acquired by UHPLC-Q-Orbitrap-MS in positive and negative ion mode was presented in Figure 1. By comparing the chemical composition of MS1 and MS2 fragments with data from the mzVault database and references, a total of 70 compounds were identified, including 23 flavonoids, 15 phenol compounds, 17 terpenoid compounds, and 15 other types of compounds (Table 1, Supplementary Material). Twenty-three identified flavonoid compounds were procyanidin B1 (14), epicatechin (15), (+)-catechin hydrate (16), procyanidin B2 (17), cianidanol (18), 2'-*O*-galloylhyperin (23), isorhamnetin (24), (−)-epicatechin gallate (27), isoquercitrin (28), astilbin (29), astragalin (31), kaempferol (32), isosakuranetin (33), trilobatin (34), hesperetin (35), quercitrin (38), phloridzin (42), licochalcone B (45), morin (46), phloretin (49), eupafolin (51), cinnamaldehyde (52), and dichotomitin (54). Fifthteen identified phenol compounds were *L*-tyrosine (7), gallic acid (9), corilagin (10), 2-hydroxy-4-methoxybenzaldehyde (20), ethyl gallate (21), 3,5-dimethoxy-4-hydroxybenzaldehyde (22), dihydroresveratrol (25), ellagic acid (26), 1,2,3,4,6-pentagalloylglucose (30), ferulaldehyde (36), sinapyl aldehyde (41), orsellinic acid (43), *o*-veratraldehyde (47), astringin (50), and ethylparaben (70). Seventeen identified terpenoid compounds were ailanthone (39), *α*-cyperone (44), atractyloside A (48), medicagenic acid (55), echinocystic acid (56), 18*β*-glycyrrhetintic acid (57), quillaic acid (58), maslinic acid (59), bayogenin (60), acetyl-11-keto-*β*-boswellic acid (61), 3-*O*-acetyl-16*α*-hydroxytrametenolic acid (63), ursolic acid (64), oleanonic acid (65), lupenone (66), roburic acid (67), *α*-boswellic acid (68), and *β*-elemonic acid (69). Besides, 15 other types of compounds were identified from *C. oleifera* bud EE, including *γ*-aminobutyric acid (1), quinic acid (2), 2-pyrrolidinecarboxylic acid (3), citric acid (4), *L*-pyroglutamic acid (5), *L*-phenylalanine (6), *L*-leucine (8), nicotinic acid (11), *L*-tryptophan (12), 2-isopropylmalic acid (13), benzoic acid (19), 7-methoxycoumarin (37), azelaic acid (40), sauchinone (53), and *α*-linolenic acid (62). Except for gallic acid (9), kaempferol (32), and oleanonic acid (65) (Sugimoto et al., 2009; Ma, 2019; Luan et al., 2020), the remaining 67 compounds were first identified from *C. oleifera* bud. The above data indicated that *C. oleifera* bud EE was rich in terpenoids, flavonoids, and phenolic compounds.

**TABLE 1:** Phytochemical compounds of *Camellia oleifera* bud EE were detected and characterized using UHPLC-Q-Orbitrap-MS in positive and negative ionization modes.

Peak NO.	RT [min]	Identification^a^	Formula	[M + H]^+^ (m/z)	[M-H]^-^ (m/z)	Error ppm	MS^2^ fragment ions
1	0.91	*γ*-Aminobutyric acid	C_4_H_9_NO_2_	104.07043		−1.7	87.04406, 86.06005, 69.03378, 60.08126, 58.06558
2	0.96	Quinic acid	C_7_H_12_O_6_		191.05440	−9.0	173.04369, 155.03320, 127.03826, 111.04336, 109.02776
3	1.04	2-Pyrrolidinecarboxylic acid	C_5_H_9_NO_2_	116.07013		−4.1	116.07010, 70.06548, 68.04980
4	1.11	Citric acid	C_6_H_8_O_7_		191.01796	−9.2	173.00717, 129.01765, 111.00700, 87.00703, 85.02776
5	1.81	*L*-Pyroglutamic acid	C_5_H_7_NO_3_	130.04919		−5.2	112.07528, 102.05456, 97.00763, 84.04452, 70.06536
6	1.96	*L*-Phenylalanine	C_9_H_11_NO_2_	166.08536		−5.4	120.08038, 103.05396, 91.05416
7	2.20	*L*-Tyrosine	C_9_H_11_NO_3_	182.08015		−5.6	165.05351, 147.04303, 136.07487, 123.04343, 119.04858
8	2.25	*L*-Leucine	C_6_H_13_NO_2_	132.10123		−5.1	114.09131, 86.09682, 69.07053
9	2.43	Gallic acid	C_7_H_6_O_5_		169.01248	−9.5	125.02268, 107.01203, 97.2773
10	3.33	Corilagin	C_27_H_22_O_18_		633.07056	−4.4	300.99738, 275.01837, 229.01262, 169.01250, 125.02256
11	4.29	Nicotinic acid	C_6_H_5_NO_2_	124.03875		−4.5	106.02863, 96.04424, 80.04961, 78.03397
12	7.05	*L*-Tryptophan	C_11_H_12_N_2_O_2_	205.09587		−6.3	188.06924, 170.05893, 146.05901, 144.07974, 132.07991
13	8.45	2-Isopropylmalic acid	C_7_H_12_O_5_		175.05936	−9.5	157.04880, 115.03831, 113.05902, 85.06414
14	8.83	Procyanidin B1	C_30_H_26_O_12_		577.13220	−5.1	451.09979, 425.08585, 407.07483, 289.07013, 287.05460
15	9.33	Epicatechin	C_15_H_14_O_6_	291.08438		−6.6	273.07382, 249.07423, 179.06845, 165.05342, 139.03799
16	9.49	(+)-Catechin hydrate	C_15_H_14_O_6_		289.07025	−5.2	245.08026, 227.06969, 205.04890, 203.06972, 151.03824
17	10.63	Procyanidin B2	C_30_H_26_O_12_		579.14630	4.3	427.10019, 409.08905, 291.08432, 289.06900, 139.03792
18	10.94	Cianidanol	C_15_H_14_O_6_	291.08450		−6.2	273.07446, 179.06909, 147.04301, 139.03802, 123.04340
19	11.23	Benzoic acid	C_7_H_6_O_2_	123.04334		−5.8	105.03313, 95.04899, 77.03870
20	11.84	2-Hydroxy-4-methoxybenzaldehyde	C_8_H_8_O_3_	153.05371		−6.0	125.05903, 121.02778, 111.04371, 93.03341
21	12.17	Ethyl gallate	C_9_H_10_O_5_		197.04384	−8.7	169.01239, 151.00180, 125.02257, 124.01472
22	12.73	3,5-Dimethoxy-4-hydroxybenzaldehyde	C_9_H_10_O_4_	183.06406		−6.1	155.06918, 140.04584, 123.04350, 95.04913
23	12.97	2'-*O*-Galloylhyperin	C_28_H_24_O_16_		615.09589	−5.3	463.08563, 301.03372, 300.02588, 271.02310, 255.02821
24	13.13	Isorhamnetin	C_16_H_12_O_7_	317.06351		−6.5	302.00400, 285.00095, 257.00607, 165.05336, 107.04890
25	13.21	Dihydroresveratrol	C_14_H_14_O_3_	231.10002		−6.7	137.05884, 125.05910, 121.06423, 107.04884, 93.06979
26	13.27	Ellagic acid	C_14_H_6_O_8_		300.99741	−5.3	257.00760, 229.01253, 201.01765, 185.02252, 145.02768
27	13.39	(−)-Epicatechin gallate	C_22_H_18_O_10_		441.08023	−5.6	289.07028, 245.08020, 169.01245, 125.02263, 109.02773
28	13.70	Isoquercitrin	C_21_H_20_O_12_	465.10043		−5.0	303.04776, 285.03796, 257.04263, 153.01694, 137.02264
29	13.90	Astilbin	C_21_H_22_O_11_		449.10654	−5.3	431.05960, 303.04785, 285.03873, 151.00189, 125.02262
30	13.93	1,2,3,4,6-Pentagalloylglucose	C_41_H_32_O_26_		939.10663	−4.6	787.09735, 769.08594, 617.07489, 465.06393, 313.05508
31	14.30	Astragalin	C_21_H_20_O_11_	449.10526		−5.7	287.05304, 259.05972, 165.01686, 153.01730, 121.02766
32	14.30	Kaempferol	C_15_H_10_O_6_	287.05322		−6.3	269.04196, 259.05841, 165.01712, 153.01717, 121.02775
33	14.34	Isosakuranetin	C_16_H_14_O_5_	287.09351		7.3	165.01712, 153.01717, 137.02225, 107.04870
34	14.35	Trilobatin	C_21_H_24_O_10_		435.12729	−5.5	315.08569, 273.07520, 179.03323, 167.03316, 137.05902
35	14.39	Hesperetin	C_16_H_14_O_6_	303.08383		−8.2	285.03781, 257.04251, 153.01721, 137.02249
36	14.66	Ferulaldehyde	C_10_H_10_O_3_	179.06908		−6.7	164.04585, 161.05861, 151.03781, 147.04311, 123.04353
37	14.90	7-Methoxycoumarin	C_10_H_8_O_3_	177.05350		−6.3	162.06641, 145.06406, 133.06400, 117.06933, 91.05413
38	15.00	Quercitrin	C_21_H_20_O_11_		447.09085	−5.4	301.03366, 300.02582, 271.02325, 255.02840, 178.99661
39	15.03	Ailanthone	C_20_H_24_O_7_	377.15692		−6.8	349.04330, 331.03088, 181.08484, 163.07410, 151.03786
40	15.12	Azelaic acid	C_9_H_16_O_4_		187.09579	−9.6	169.08522, 125.09540, 97.06409
41	15.14	Sinapyl aldehyde	C_11_H_12_O_4_	209.07951		−6.3	194.05582, 191.06888, 181.08464, 177.05339, 153.05353
42	15.54	Phloridzin	C_21_H_24_O_10_		435.12726	−5.5	273.07538, 255.06435, 167.03322, 123.04335
43	15.55	Orsellinic acid	C_8_H_8_O_4_	169.04840		−6.7	151.03795, 123.04345, 109.06463, 95.04911
44	16.95	*α*-Cyperone	C_15_H_22_O	219.17287		−6.7	201.08958, 189.08969, 105.06953, 67.05454
45	17.03	Licochalcone B	C_16_H_14_O_5_	287.08942		−6.9	165.01758, 153.01717, 137.02289, 121.02781, 91.05396
46	17.50	Morin	C_15_H_10_O_7_		301.03375	−5.4	273.03891, 229.04890, 193.01254, 151.00189, 107.01204
47	17.79	*o*-Veratraldehyde	C_9_H_10_O_3_	167.06929		−5.9	152.04579, 134.03499, 123.04339, 95.04888
48	18.10	Atractyloside A	C_21_H_36_O_10_		447.22113	−5.4	315.18069, 179.05435, 113.02264, 101.02264
49	19.13	Phloretin	C_15_H_14_O_5_		273.0755	−4.9	255.06511, 167.03323, 151.00209, 149.02246, 119.04848
50	19.20	Astringin	C_20_H_22_O_9_		405.11694	−2.2	243.06473, 215.06960, 135.00700, 123.00700
51	19.51	Eupafolin	C_16_H_12_O_7_		315.04938	−5.2	300.02582, 287.05478, 272.03140, 271.02377
52	20.15	Cinnamaldehyde	C_9_H_8_O	133.06406		−5.5	115.05382, 105.06958, 103.05398, 91.05408, 79.05437
53	20.20	Sauchinone	C_20_H_20_O_6_	357.13080		−6.9	339.11850, 327.12097, 309.10880, 219.07890, 203.06882
54	24.58	Dichotomitin	C_18_H_14_O_8_	359.07404		−5.9	344.07355, 329.02664, 301.03250, 285.03696, 257.04254
55	25.42	Medicagenic acid	C_30_H_46_O_6_		501.31955	−5.2	483.30869, 465.29675, 439.31976, 421.31024, 393.27829
56	32.72	Echinocystic acid	C_30_H_48_O_4_		471.34589	−4.4	453.33438, 425.33344, 407.32996, 391.29947, 373.25339
57	32.77	18*β*-Glycyrrhetintic acid	C_30_H_46_O_4_	471.34482		−4.4	425.33859, 407.32861, 317.20908, 271.20364, 235.16823
58	34.80	Quillaic acid	C_30_H_46_O_5_		485.3251	−4.4	467.31140, 439.32510, 423.32510, 393.32126, 377.28320
59	35.32	Maslinic acid	C_30_H_48_O_4_	473.36053		−4.2	437.33963, 427.35474, 409.34610, 247.16728, 207.17299
60	36.23	Bayogenin	C_30_H_48_O_5_		487.34082	−4.3	469.33301, 425.34207, 409.30930, 407.29492, 393.27802
61	36.57	Acetyl-11-keto-*β*-boswellic acid	C_32_H_48_O_5_	513.35504		−4.7	467.34991, 271.20413, 235.16748, 189.16273, 217.15742
62	37.44	*α*-Linolenic acid	C_18_H_30_O_2_	279.23059		−4.5	261.21979, 243.20946, 123.11636, 109.10092, 81.07011
63	37.50	3-*O*-Acetyl-1*α*-hydroxytrametenolic acid	C_32_H_50_O_5_		513.35626	−4.5	497.32782, 453.33615, 451.31570, 393.31430, 59.01230
64	37.74	Ursolic acid	C_30_H_48_O_3_	457.36511		−5.5	439.37219, 411.36008, 393.35007, 249.18439, 203.17842
65	39.62	Oleanonic acid	C_30_H_46_O_3_		453.33585	−3.5	407.33038, 391.29877, 377.28339
66	39.74	Lupenone	C_30_H_48_O	425.37589		−4.5	407.36414, 217.19411, 215.17825, 203.17888, 191.17831
67	41.57	Roburic acid	C_30_H_48_O_2_	441.37109		−3.7	423.26172, 219.17332, 207.17349, 189.16287, 147.11618
68	41.98	*α*-Boswellic acid	C_30_H_48_O_3_	457.36813		−1.3	439.35559, 263.20038, 235.16800, 207.17345, 189.16286
69	45.53	*β*-Elemonic acid	C_30_H_46_O_3_	455.34958		−5.0	437.33835, 325.28699, 245.22603, 237.14705, 229.19455
70	49.79	Ethylparaben	C_9_H_10_O_3_	167.06908		−5.0	149.02258, 139.07452, 123.04351, 107.08511, 95.04925

Identification: Based on comparison with mzVault database and references (Supplementary Material).

### 3.2 Cytotoxic activity of *Camellia oleifera* bud EE

**FIGURE 2:**
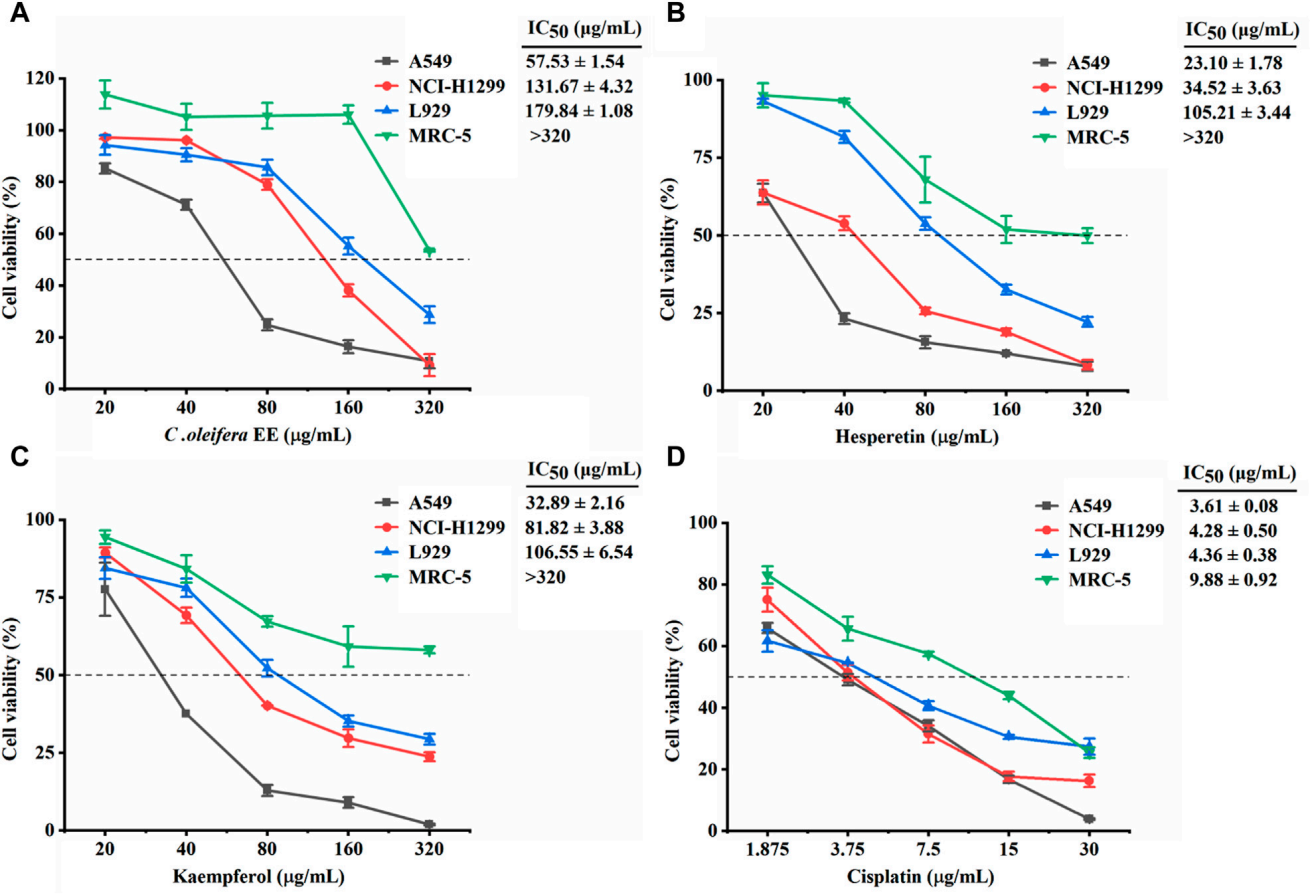
Effect of *Camellia oleifera* bud EE on the viability of cancerous cells (A549 and NCI-H1299) and non-cancerous cells (L929 and MRC-5). Cytotoxic activities of *Camellia oleifera* bud EE **(A)**, hesperetin **(B)**, kaempferol **(C)**, and cisplatin **(D)** were tested using MTT assay. Cisplatin was a positive control. IC_50_: Sample concentration reducing cell growth by 50%.

The cytotoxic activities of *C. oleifera* bud EE on cancerous cells (A549 and NCI-H1299) and non-cancerous cells (L929 and MRC-5) were estimated using MTT assay. Cisplatin was used as the positive control. As presented in Figure 2, *C. oleifera* bud EE showed higher toxicity on cancerous cells A549 (IC_50_: 57.53 ± 1.54 μg/mL) and NCI-H1299 (IC_50_: 131.67 ± 4.32 μg/mL), while displayed lower toxicity on non-cancerous cell lines MRC-5 (IC_50_: >320 μg/mL) and L929 (IC_50_: 179.84 ± 1.08 μg/mL). These results indicated that *C. oleifera* bud EE displayed selective cytotoxicity against cancerous cells, especially A549 cells. Thus, the anticancer effects of *C. oleifera* bud EE on A549 cells were selected for subsequent studies.

According to network pharmacology and molecular docking results, hesperetin, kaempferol, isorhamnetin, cianidanol, ellagic acid, licochalcone B, morin, and procyanidin B1 identified from *C. oleifera* bud EE play an important role in the treatment of NSCLC (Supplementary Material). Hesperetin and kaempferol were chosen as representatives to detect cytotoxicity (Figures 2B, C). Hesperetin and kaempferol exhibited greater cytotoxicity to cancer cells A549 (IC_50_: 23.10 ± 1.78 and 32.89 ± 2.16 μg/mL, respectively) and NCI-H1299 (IC_50_: 34.52 ± 3.63 and 81.82 ± 3.88 μg/mL, respectively) and were less toxic to non-cancer cells MRC-5 (IC_50_: >320 μg/mL) and L929 (IC_50_: 105.21 ± 3.44 and 106.55 ± 6.54 μg/mL, respectively). Thus, hesperetin and kaempferol showed selective cytotoxicity to cancer cells, in particular to A549 cells.

### 3.3 *Camellia oleifera* bud EE inhibited proliferation of A549 cells

**FIGURE 3:**
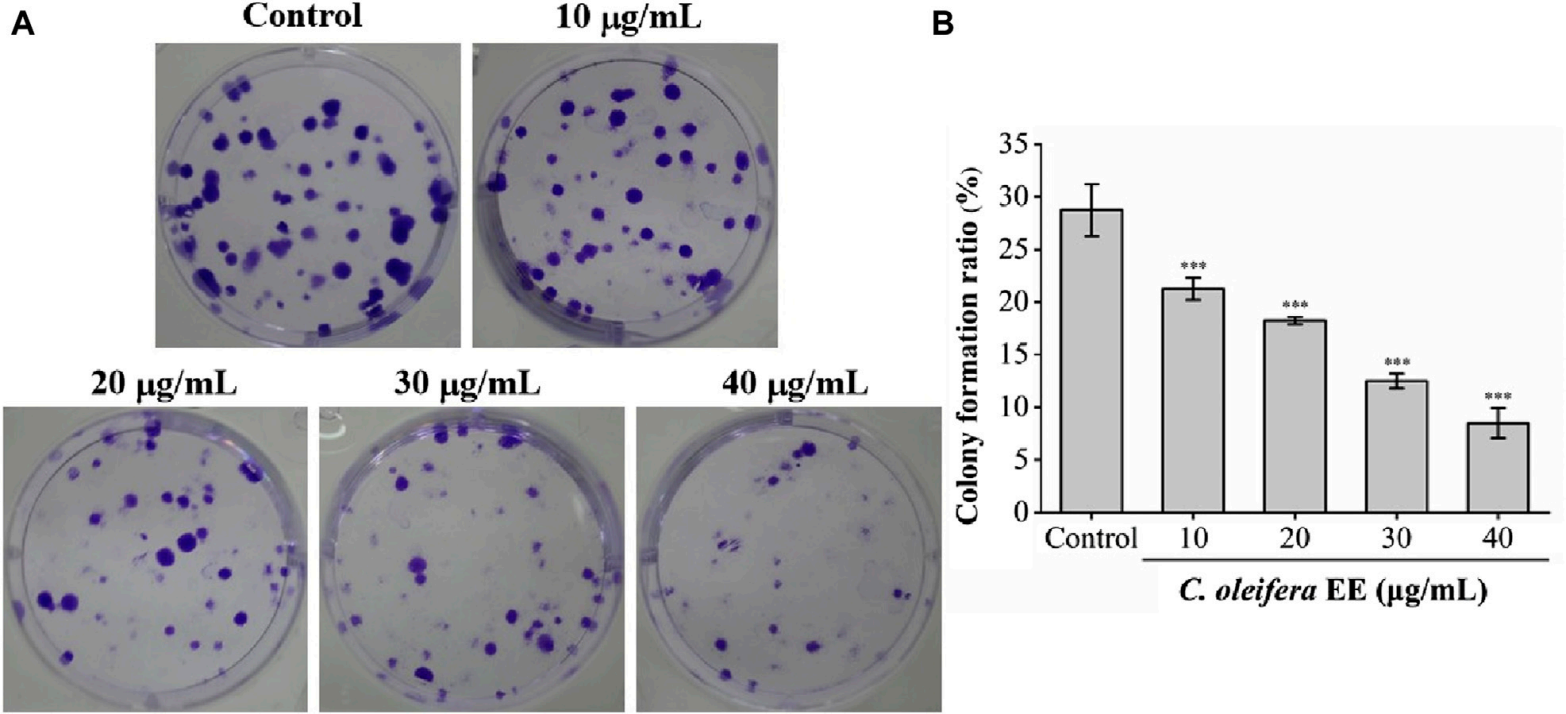
*Camellia oleifera* bud EE suppressed colony formation of A549 cells. **(A)** Colony formation assay. **(B)** The colony formation ratio (%) of A549 cells. Data were presented as means ± SD. ***p < 0.001 *versus* the control group.

The anti-proliferative activity of *C. oleifera* bud EE was evaluated using a cell colony formation assay. *Camellia oleifera* bud EE dramatically reduced the size and number of A549 cell colonies (Figure 3A). As shown in Figure 3B, compared with the control group (clone formation rate: 28.75% ± 2.48%), the clone formation rates of A549 cells treated with different doses of *C. oleifera* bud EE (10, 20, 30, and 40 μg/mL) were significantly reduced to 21.25% ± 1.06%, 18.25% ± 0.35%, 12.50% ± 0.71%, and 8.50% ± 1.41%, respectively. The above data demonstrated that *C. oleifera* bud EE concentration-dependently inhibited the proliferation of A549 cells.

**FIGURE 4:**
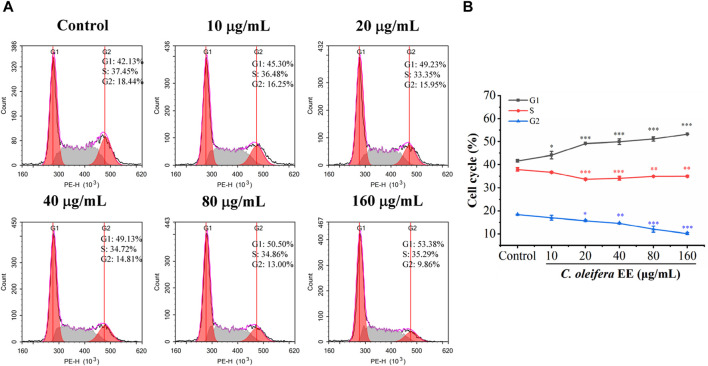
*Camellia oleifera* bud EE induced cell cycle arrest at G1 phase in A549 cells. **(A)** A549 cells were treated with indicated concentrations of *Camellia oleifera* bud EE and detected using flow cytometry. **(B)** The proportion of cells in the G1, S, and G2 phases. Data were presented as means ± SD. *p < 0.05, **p < 0.01, ***p < 0.001 *versus* the control group.

The malignant proliferation of tumor cells is closely related to cell cycle dysregulation (Diaz-Moralli et al., 2013). To determine whether the antiproliferative effect of *C. oleifera* bud EE was caused by cell cycle arrest, we examined its impact on the cell cycle (Figure 4). The proportions of G1 phase cells following treatment with *C. oleifera* bud EE at concentrations of 10, 20, 40, 80, and 160 μg/mL were raised from 41.70% ± 0.61% in the control to 44.16% ± 1.62%, 49.22% ± 0.02%, 49.97% ± 1.19%, 51.18% ± 0.96%, and 53.33% ± 0.08%, respectively. The above findings suggested that *C. oleifera* bud EE suppressed A549 cell proliferation by arresting the cell cycle at the G1 phase.

### 3.4 *Camellia oleifera* bud EE induced A549 cells apoptosis

**FIGURE 5:**
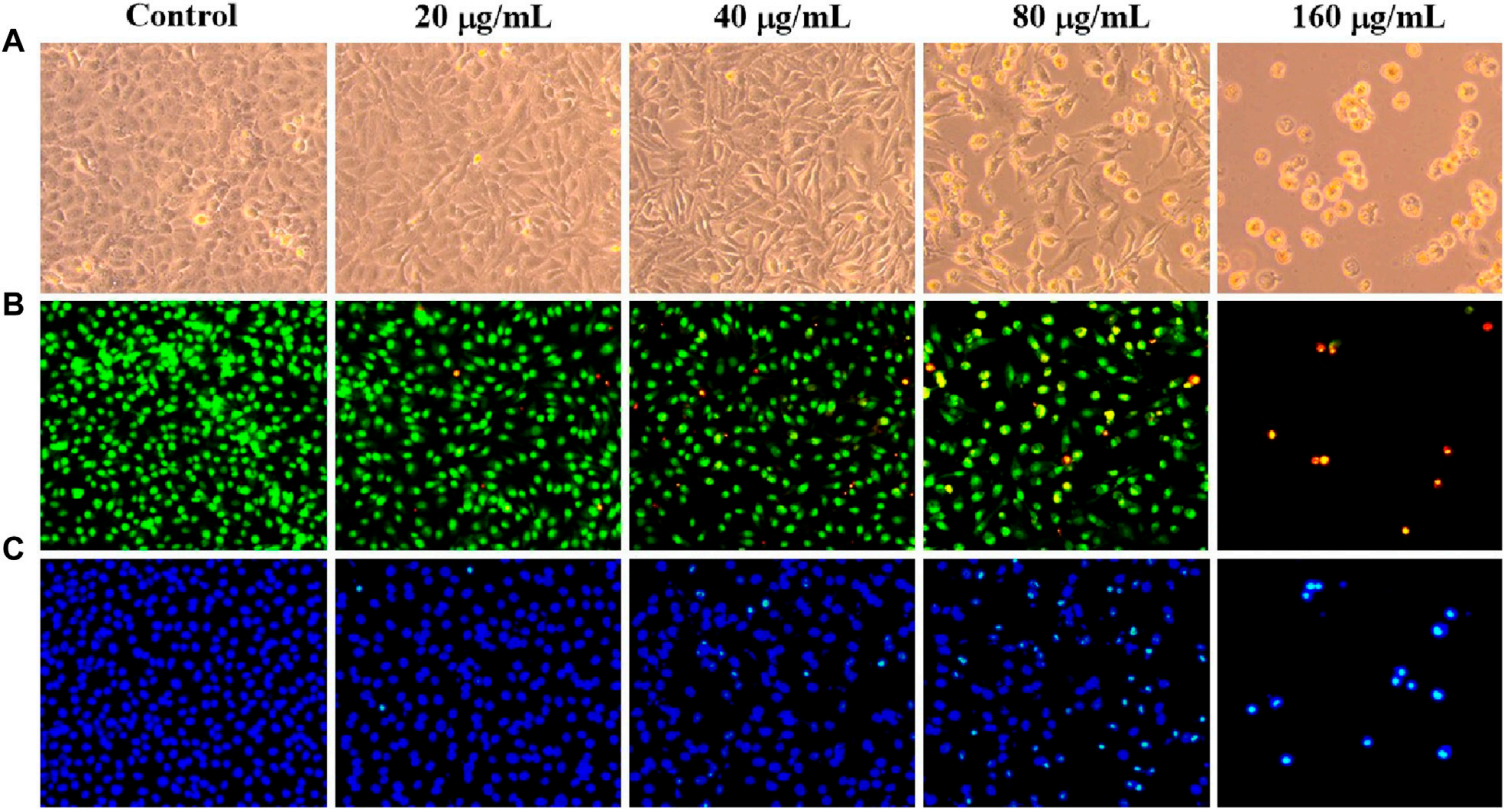
*Camellia oleifera* bud EE induced A549 cell apoptosis. **(A)** Morphological changes of A549 cells were visualized under a phase contrast microscope. **(B, C)** The nuclear morphology changes of A549 cells were examined using AO/EB **(B)** and Hoechst 33258 **(C)** staining and observed under an inverted fluorescence microscope.

Avoiding apoptosis is one of the hallmarks of cancer, and inducing apoptosis has become a key therapeutic strategy (Kornienko et al., 2013). Under an inverted microscope, *C. oleifera* bud EE-treated A549 cells displayed typical morphological apoptotic alterations like cell rounding and shrinkage (Figure 5A). In addition, AO/EB staining and Hoechst 33,258 staining were used to examine nuclear morphological changes in A549 cells. In the AO/EB staining assay, after *C. oleifera* bud EE treatment, the proportion of live cells with green fluorescent nuclei decreased, while the proportion of apoptotic cells with orange-red fluorescent nuclei increased (Figure 5B). Hoechst 33,258 staining (Figure 5C) displayed that the proportion of bright blue fluorescent cells with dense nuclei increased gradually after treatment with *C. oleifera* bud EE, which had the characteristics of apoptosis.

**FIGURE 6:**
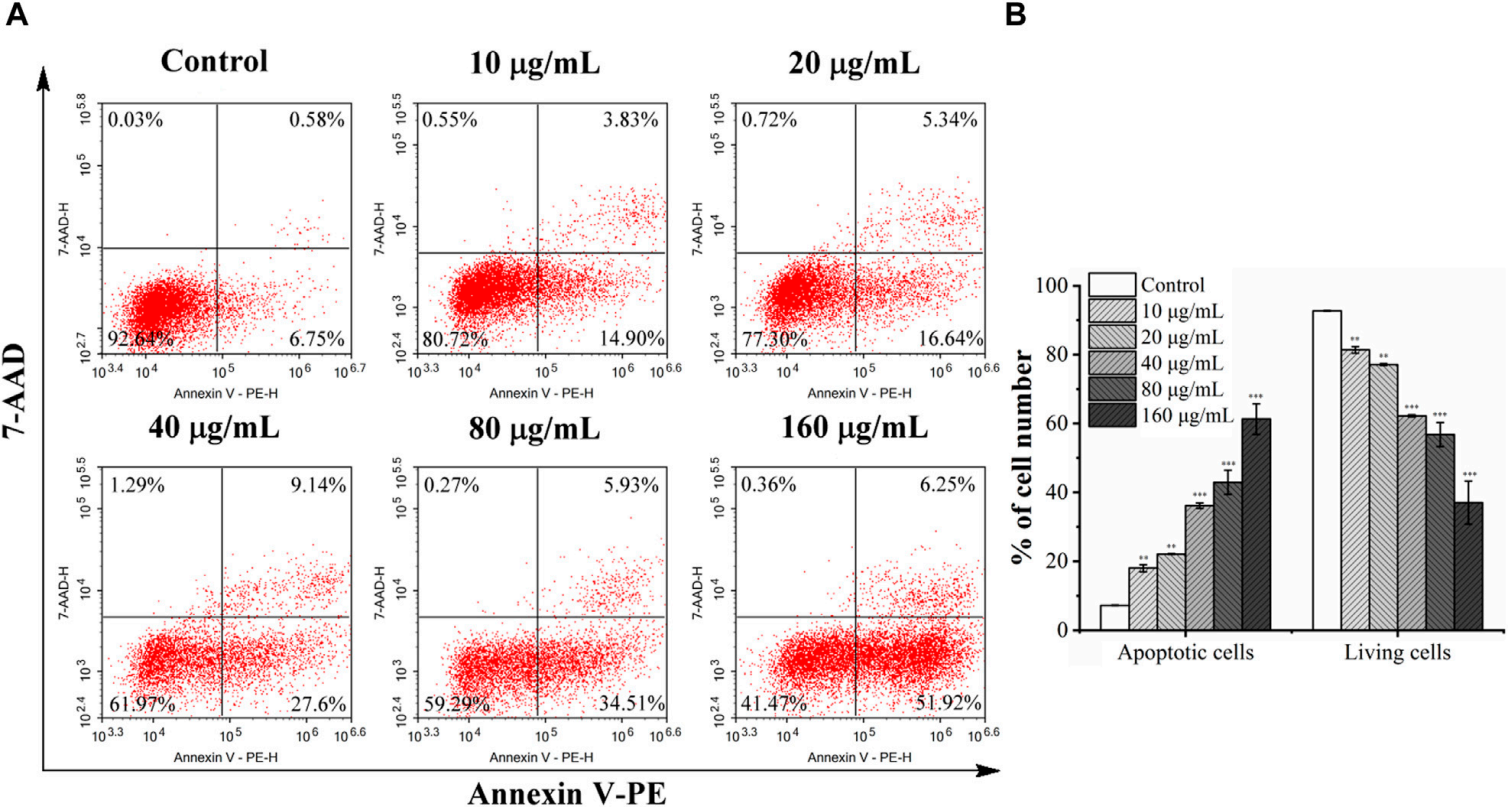
*Camellia oleifera* bud EE induced A549 cells apoptosis. **(A)** After treatment with specified concentrations of *Camellia oleifera* bud EE, A549 cells were labeled with Annexin V-PE and 7-AAD and detected by a flow cytometer. Cells in the upper right quadrant (PE+/7-AAD+): late apoptotic cells; lower right quadrant (PE+/7-AAD–): early apoptotic cells; upper left quadrant (PE–/7-AAD+): necrotic cells; lower left quadrant (PE–/7-AAD–): live cells. **(B)** The proportion of living and apoptotic cells. Data were expressed as means ± SD. **p < 0.01, ***p < 0.001 *versus* the control group.

Flow cytometry was used to quantitatively evaluate apoptosis induced by *C. oleifera* bud EE. As shown in Figure 6, the percentage of apoptotic cells after treatment with *C. oleifera* bud EE increased significantly. The apoptotic rates increased from 7.28% ± 0.08% of untreated cells to 18.04% ± 0.98% at 10 μg/mL, 22.09% ± 0.16% at 20 μg/mL, 36.18% ± 0.80% at 40 μg/mL, 42.92% ± 3.51% at 80 μg/mL, and 61.31% ± 4.43% at 160 μg/mL. The above results suggested that *C. oleifera* bud EE induced A549 cell apoptosis in a concentration-dependent manner.

### 3.5 *Camellia oleifera* bud EE decreased mitochondrial membrane potential of A549 cells

**FIGURE 7:**
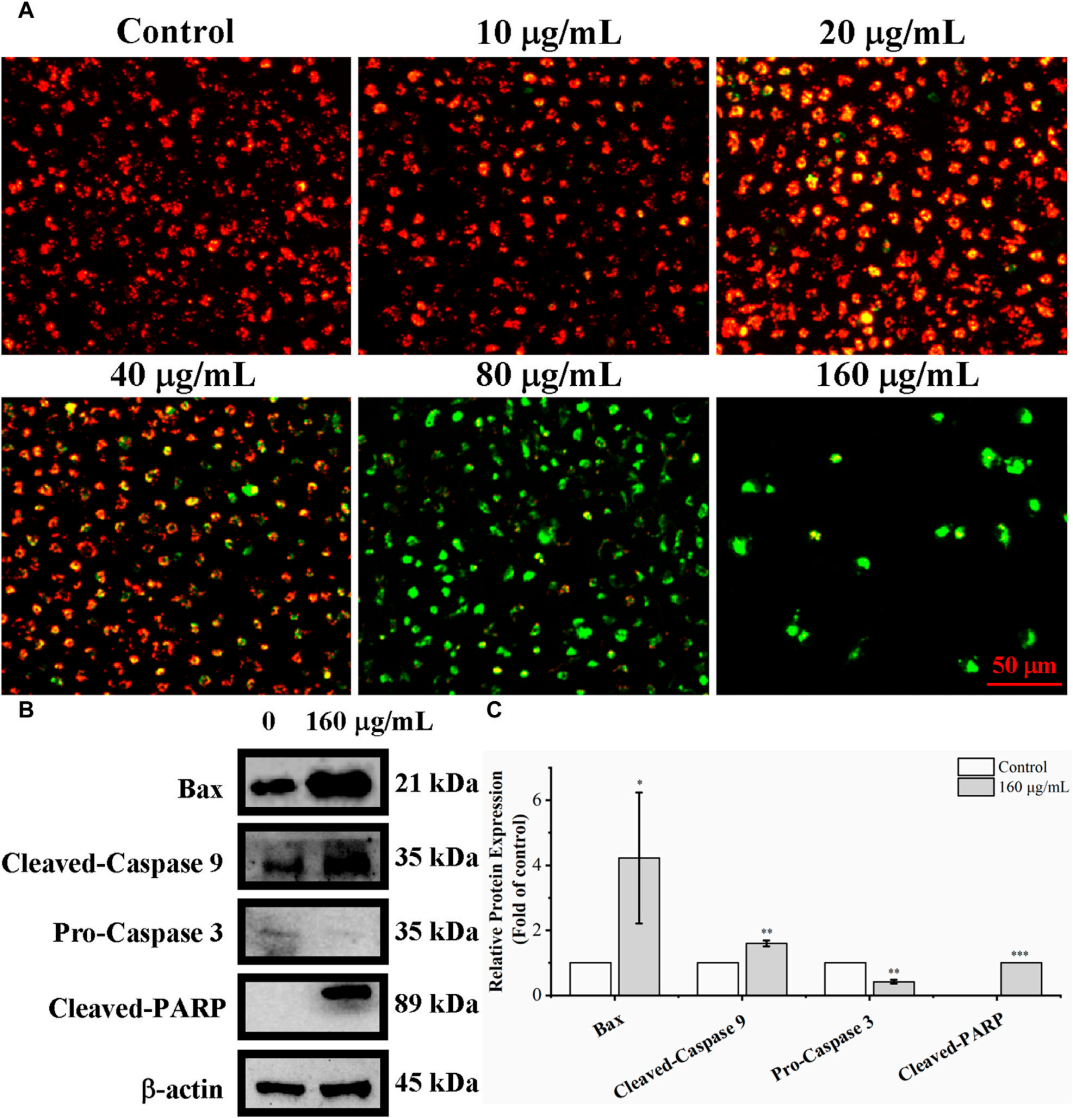
*Camellia oleifera* bud EE induced A549 apoptosis by mitochondria-mediated pathway. **(A)** Mitochondrial membrane potential in *Camellia oleifera* bud EE-treated A549 cells was detected by JC-1 staining and photographed under an inverted fluorescence microscope. **(B)** Western blot detection for the expression levels of Bax, cleaved-caspase 9, pro-caspase 3, and cleaved-PARP proteins in *Camellia oleifera* bud EE-treated cells. **(C)** The relative expression levels of related proteins. Results were shown as the mean ± SD. *p < 0.05, **p < 0.01, ***p < 0.001 *versus* the control group.

The loss of mitochondrial membrane potential is one of the key events in apoptosis (Tsujimoto and Shimizu, 2007). Mitochondrial membrane potential was detected with the fluorescent probe JC-1 to determine whether the loss of mitochondrial transmembrane potential (ΔΨm) is related to *C. oleifera* bud EE-activated apoptosis. When stained with JC-1 dye, apoptotic cells with low ΔΨm emit green fluorescence (JC-1 monomers), whereas normal cells with high ΔΨm emit red fluorescence (JC-1 aggregates). As shown in Figure 7, after treating A549 cells with different concentrations of *C. oleifera* bud EE, the proportion of red fluorescent cells gradually decreased, and the proportion of green fluorescent cells gradually increased. In particular, after *C. oleifera* bud EE treatment at doses of 80 μg/mL and 160 μg/mL, the cells almost entirely displayed green fluorescence. These results revealed that *C. oleifera* bud EE induced A549 cell apoptosis by reducing mitochondrial membrane potential.

Proteins associated with the mitochondria-mediated apoptosis pathway were detected by Western blot. As shown in Figures 7B, C, *C. oleifera* bud EE upregulated the levels of Bax, cleaved-caspase 9, and cleaved-PARP and downregulated the expression of pro-caspase 3. These results indicated that it activated caspase 9 and caspase 3 by upregulating Bax, thereby leading to the cleavage of PARP. Hence, *C. oleifera* bud EE induced A549 cell apoptosis *via* the mitochondrion-mediated pathway.

### 3.6 *Camellia oleifera* bud EE inhibited the migration and invasion ability of A549 cells

**FIGURE 8:**
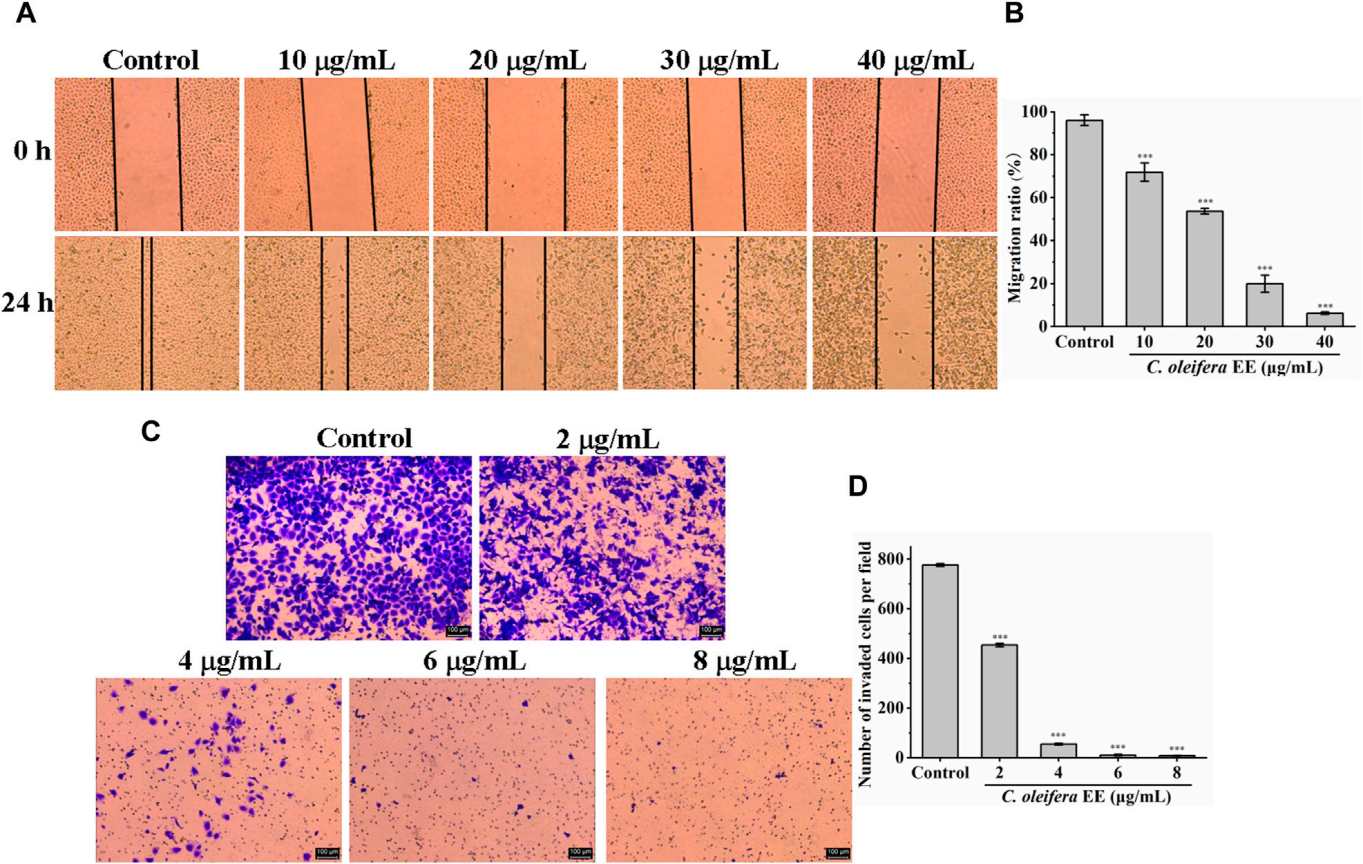
*Camellia oleifera* bud EE inhibited the invasion and migration abilities of A549 cells. **(A)** Wound healing assay detected the migratory potential (×50). **(B)** Quantitative analysis of the migratory capacity through migration ratio (%). **(C)** Transwell invasion assay measured the invasive ability. **(D)** Quantitative analysis of the invasive capacity through the average number of invaded A549 cells per field. Data were presented as mean ± SD. ***p < 0.001 compared with the control group.

The metastasis of cancer from the original site to distant organs is the main cause of cancer death (Riihimäki et al., 2014). To evaluate the impact of *C. oleifera* bud EE on the migration capability, a wound healing test was performed. As illustrated in Figures 8A, B, the migration rates of A549 cells treated with 10, 20, 30, and 40 μg/mL *C oleifera* bud EE were 71.83% ± 4.25%, 53.60 ± 1.23, 20.01% ± 3.94%, and 6.20% ± 0.59%, respectively, which were significantly lower than that of the control group (96.04% ± 2.46%). Transwell invasion assay was performed to assess the impact of *C. oleifera* bud EE on the invasive ability of A549 cells. As shown in Figures 8C, D, compared with the control group, the number of invasive cells in the *C. oleifera* bud EE treatment group was significantly reduced in a dose-dependent manner. All these results suggested that *C. oleifera* bud EE repressed the migration and invasion abilities of A549 cells.

## 4 Discussion

UHPLC-Q-Orbitrap-MS was used to identify the chemical components of *C. oleifera* bud EE, and 70 compounds were identified. According to the network pharmacology analysis (Supplementary Figure S1, Supplementary Material), 10 potential active components (astilbin, cianidanol, ellagic acid, hesperetin, isorhamnetin, kaempferol, licochalcone B, morin, procyanidin B1, and *α*-boswellic acid) and 3 core target proteins (Epidermal growth factor receptor, EGFR; RAC-alpha serine/threonine-protein kinase, AKT1; Heat shock protein HSP 90-beta, HSP90AB1) were screened out. The molecular docking method was employed to further validate the binding of target proteins and active components (Supplementary Figure S3, Supplementary Material). Their binding affinities were lower than −6, indicating they possessed potent binding activities. As shown in Supplementary Table S1, the binding energies of AKT1 to ellagic acid, isorhamnetin, and kaempferol were −6, −6.1, and −6 kcal/mol, respectively. The binding energies of EGFR to these active components were as follows: −8 kcal/mol (ellagic acid), −8 kcal/mol (isorhamnetin), −8.1 kcal/mol (kaempferol), −7.3 kcal/mol (licochalcone B), −8 kcal/mol (morin), and −10.1 kcal/mol (procyanidin B1). Additionally, the binding energies of HSP90AB1 to cianidanol, ellagic acid, hesperetin, isorhamnetin, kaempferol, licochalcone B, morin, and procyanidin B1 were −8.1, −8.7, −8.7, −8.8, −8.5, −7.7, −8.8, and −9.6 kcal/mol, respectively. AKT1 is involved in various physiological processes of cancer cells, including cell proliferation, cell cycle control, apoptosis, cell metastasis, etc. (Fortier et al., 2011). EGFR is closely related to cancer cell proliferation, apoptosis, and metastasis (Wee and Wang, 2017). HSP90AB1 protein participates in multiple cancer hallmarks, such as evasion of apoptosis, unlimited proliferation, as well as tissue invasion and metastasis (Youssef et al., 2023). Blocking AKT1, EGFR, and HSP90AB1 can inhibit proliferation, induce apoptosis, and suppress metastasis. Thus, these compounds (cianidanol, ellagic acid, hesperetin, isorhamnetin, kaempferol, licochalcone B, morin, and procyanidin B1) may affect the proliferative, apoptotic, and metastatic abilities of A549 cells by modulating three targets (AKT1, EGFR, and HSP90AB1).

Based on a previous study, ellagic acid suppressed proliferation, blocked the cell cycle, and induced apoptosis of A549 cells by restraining the PI3K/Akt signaling pathway (Liu et al., 2018). Hesperetin suppressed A549 cell proliferation and induced mitochondria-dependent apoptosis *via* Hsp70-mediated activation of Bax (Tanaka et al., 2022). Previous studies revealed that isorhamnetin inhibited A549 cell proliferation and induced apoptosis *in vitro* and *in vivo* by down-regulating Bcl-2 and upregulating Bax and caspase 3 (Li et al., 2015; Luo et al., 2019). Kaempferol induced apoptosis in lung cancer A549 cells by inactivating AKT1, downregulating the expression levels of Bcl-2 and Bcl-xL, upregulating the expression levels of Bax, and cleaving PARP (Nguyen et al., 2003). Besides, kaempferol blocked the migration of A549 cells by inhibiting AKT1-mediated phosphorylation of Smad3 at Thr179 residue (Jo et al., 2015). Licochalcone B has been reported to suppress NSCLC cell proliferation and induce apoptosis through targeting EGFR (Oh et al., 2019). According to past research, morin suppressed lung cancer A549 cell viability, proliferation, and migration (Yao et al., 2017). Therefore, the anti-NSCLC effect of *C. oleifera* bud EE may be related to the induction of apoptosis and inhibition of proliferation and metastasis of A549 cells by these compounds.

According to the MTT results, *C. oleifera* bud EE had high toxicity to A549 cells and low toxicity to non-cancer cells (L929 and MRC-5). Therefore, the anticancer effects of *C. oleifera* bud EE on A549 cells were further studied. In addition, hesperetin and kaempferol identified from *C. oleifera* bud EE were chosen as representatives to detect cytotoxicity. Our results indicated that hesperetin and kaempferol had greater cytotoxicity to A549 cells and were less toxic to non-cancer MRC-5 and L929 cells. According to a previous study, after 48 h of treatment, kaempferol inhibited the cell viability of A549 (IC_50_ = 105.4 μM) and H1299 (570.0 μM) cells in a dose-dependent manner (Wang et al., 2023). Hesperetin suppressed A549 cell viability in a concentration-dependent manner, with an IC_50_ value of 520 µM (Tanaka et al., 2022). Hence, hesperetin and kaempferol may play an important role in the cytotoxicity of *C. oleifera* bud EE.

Uncontrolled proliferation is a characteristic of malignant cells and is associated with cell cycle dysregulation (Diaz-Moralli et al., 2013). According to the results of colony formation assay and cell cycle analysis, *C. oleifera* bud EE inhibited A549 cell proliferation by arresting the cell cycle in the G1 phase. Previous studies revealed that ellagic acid suppressed cell proliferation and increased the relative proportion of A549 cells in the G1 phase (Liu et al., 2018). In addition, kaempferol, isorhamnetin, licochalcone B, procyanidin B1, and morin have been demonstrated to inhibit cancer cell proliferation by inducing cell cycle arrest (Kuo et al., 2007; Li C. et al., 2014; Oh et al., 2019; Zhu and Xue, 2019; Lei et al., 2023). Hence, the antiproliferative effect of *C. oleifera* bud EE may be attributed to the presence of these components.

Based on the results of morphological observation, AO/EB dual staining, and Hoechst 33,258 staining, A549 cells treated with EE revealed typical morphological apoptotic alterations like cell rounding, cell shrinkage, and nuclear pyknosis. Moreover, Annexin V-PE/7-AAD analysis further indicated that *C. oleifera* bud EE induced apoptosis in A549 cells in a concentration-dependent manner. Loss of ΔΨm plays an essential role in cell apoptosis (Ly et al., 2003). Mitochondrial membrane potential assay results revealed that *C. oleifera* bud EE induced A549 cell apoptosis by reducing mitochondrial membrane potential. Further Western blot detection of mitochondrion-mediated apoptosis-related proteins showed that *C. oleifera* bud EE upregulated Bax and cleaved-caspase 9 and downregulated pro-caspase 3, leading to cleavage of PARP. Hence, *C. oleifera* bud EE induced A549 cell apoptosis through the mitochondria-mediated apoptotic pathway. Kaempferol has been proven to induce apoptosis in A549 cells by increasing the expression of Bax, cleaved-caspase 3, cleaved-caspase 9, and cleaved-PARP (Nguyen et al., 2003; Qin et al., 2016). Hesperetin induced A549 cell apoptosis by activating Bax (Tanaka et al., 2022). Ellagic acid induced apoptosis in A549 cells by regulating apoptosis-related proteins Bax, Bcl-2, and caspase 3 (Liu et al., 2018). In addition, procyanidin B1 and licochalcone B have been confirmed to induce apoptosis in cancer cells (Oh et al., 2019; Lei et al., 2023). Therefore, these active ingredients may play an important role in *C. oleifera* bud EE-induced A549 cell apoptosis.

Cancer metastasis is responsible for 90% of cancer deaths, which is the main cause of cancer death (Yilmaz et al., 2007). The wound healing assay result showed that *C. oleifera* bud EE reduced the migration ability of A549 cells in a dose-dependent manner. In the transwell invasion assay, *C. oleifera* bud EE dose-dependently reduced the number of invaded cells. All these results suggested that *C. oleifera* bud EE repressed the migration and invasion abilities of A549 cells. Based on past research, kaempferol inhibited transforming growth factor-β1-induced epithelial-to-mesenchymal transition and migration in A549 cells (Jo et al., 2015). Isorhamnetin had a significant inhibitory effect on the invasion and migration of A549 cells (Luo et al., 2019). In addition, hesperetin, licochalcone B, ellagic acid have been demonstrated to possess the ability to inhibit the metastasis of cancer cells (Zhao et al., 2014; Kim et al., 2021; Dalpatraj et al., 2022). Thus, the effect of *C. oleifera* bud EE on inhibiting the metastasis of A549 cells may be related to these active constituents.

## 5 Conclusion

The current study analyzed *C. oleifera* bud EE’s chemical composition and first explored its anticancer properties. Seventy phytochemicals were identified by UHPLC-Q-Orbitrap-MS analysis, mainly including terpenes, flavonoids, and phenolic compounds. It exhibited selective cytotoxicity on A549 cells and low toxicity on non-cancerous cells. Besides, it suppressed A549 cell proliferation by arresting the cell cycle at the G1 phase, induced apoptosis through the mitochondrion-mediated pathway, and inhibited migration and invasion abilities. Therefore, *C. oleifera* bud EE has distinguished anticancer properties and can be used as a new source of natural anticancer agents.

## Data Availability

The original contributions presented in the study are included in the article/Supplementary Material, further inquiries can be directed to the corresponding authors.
