# Supplementary material for: Phytochemical analysis and anticancer effect of Camellia oleifera bud ethanol extract in non-small cell lung cancer A549 cells
Source: Front Pharmacol. 2024 Mar 28;15:1359632. doi: 10.3389/fphar.2024.1359632 (PMC11007092; doi:10.3389/fphar.2024.1359632)
Supplement: Supplementary file 1 [file DataSheet1.PDF]

Supplementary Data

1 Methods of network pharmacology

1.1. Access of potential targets of potential active components in *C. olerfera* bud EE to treat NSCLC

TCMSP (Traditional Chinese Medicine Systems Pharmacology Database and Analysis Platform, https://old.tcmsp-e.com/tcmsp.php) database was used, and oral bioavailability (OB) ≥ 0.36 and drug-likeness (DL) ≥ 0.18 were set to screen out the potential active compounds of *C. oleifera* bud EE. Canonical SMILES (Simplified Molecular Input Line Entry System) of potential active compounds were obtained through the PubChem database and then imported into SwissTargetPrediction (http://www.swisstargetprediction.ch/) to attain potential targets. The obtained targets were translated into gene names *via* the UniProt database (https://www.uniprot.org/) (UniProt Consortium, 2023).

In addition, potential target genes of non-small cell lung cancer (NSCLC) were searched in Genecards (https://www.genecards.org/), Therapeutic Target Database (TTD, https://db.idrblab.net/ttd/), OMIM database (https://www.omim.org/), and PharmGKB (https://www.pharmgkb.org/). The NSCLC-related targets and target genes of the potential active compounds in *C. oleifera* bud EE were overlapped through Venny 2.1.0 (https://bioinfogp.cnb.csic.es/tools/venny/index.html) (Oliveros, 2007), and the overlapping targets were obtained, which were potential target genes of *C. oleifera* bud EE for the treatment of NSCLC.

1.2. Protein-protein interaction (PPI) network and drug-target network construction

The PPI network of intersectional targets was constructed in the STRING database, the species were set as “Homo sapiens”, while the minimum required interaction score was set to the high confidence level (0.700), and disconnected nodes were hidden. Then, the PPI network was optimized and refined through Cytoscape 3.9.1 software. This software was extensively employed for data integration, visualization, and analysis of complex networks in network pharmacology. Besides, Cytoscape software was used to construct potential active compounds and targets network of *C. oleifera* bud EE in treating NSCLC. Centiscape 2.2 plug-in was used in Cytoscape software to analyze the network. There were 61 nodes whose degree value (degree) and betweenness centrality (BC) were higher than the mean values (degree value = 12.05283019; betweenness centrality = 529.0113208). Subsequently, the top 3 targets ranked by degree value were screened as core targets for the following analysis. Next, the Cytoscape software was also used to construct the *C. oleifera* bud EE-potential active compounds-targets network to explore the anti-NSCLC mechanism of *C. oleifera* bud EE. *C. oleifera* bud EE, components, and target genes were represented by nodes, and their interrelationships were displayed by edges.

1.3. GO and KEGG pathway enrichment

Gene Ontology (GO) and Kyoto Encyclopedia of Genes and Genomes (KEGG) pathway enrichment analysis were performed in the DAVID database (https://david.ncifcrf.gov/), and the P value was set to 0.01. The obtained data were visualized through the online tool (http://www.bioinformatics.com.cn/).

1.4. Molecular docking

The molecular docking approach was used to validate the binding of the target to the corresponding compound. The original ligand files were obtained from the TCMSP database. The crystal structures of proteins were downloaded from the PDB (RCSB Protein Data Bank, https://www.rcsb.org). Autodock 4.2 software was used to remove unwanted water molecule hydrogenation and then predict protein-ligand binding, and we visualized the results on PyMOL software.

2 Results of network pharmacology

2.1. Identification of the core targets of *C. oleifera* bud EE for treating NSCLC

Ten potential active compounds (astilbin, cianidanol, ellagic acid, hesperetin, isorhamnetin, kaempferol, licochalcone B, morin, procyanidin B1, and α-boswellic acid) were screened from the TCMSP database according to the parameter settings. A total of 382 targets were obtained through the TCMSP database and the SwissTargetPrediction database. A total of 7502 potential target genes of NSCLC were obtained from the four disease databases mentioned above. As shown in Figure S1A, the 284 overlapping targets were obtained using a Venn diagram and as potential therapeutic targets for further analysis. A PPI network of 284 overlapping target genes was constructed through the STRING (Figure S1B). Cytoscape 3.9.1 was used to construct and visualize the network, revealing the relationships of *C. oleifera* bud EE-potential active components-targets. Subsequently, the Cytoscape revealed clearer relationships and identified core targets based on degree values (Figure S1C). As shown in Figure S1D, the network was composed of 282 nodes and 1597 edges. In the network, three core target genes were identified as AKT1, EGFR, and HSP90AB1 according to degree value.

AKT1 is involved in various physiological processes of cancer cells, including cell proliferation, cell cycle control, apoptosis, cell metastasis, etc. (Fortier et al., 2011). EGFR is closely related to cancer cell proliferation, apoptosis, and metastasis (Wee and Wang, 2017). HSP90AB1 protein participates in multiple cancer hallmarks, such as evasion of apoptosis, unlimited proliferation, as well as tissue invasion and metastasis (Youssef et al., 2023). Hence, these three core targets are closely related to cancer cell proliferation, apoptosis, and metastasis.

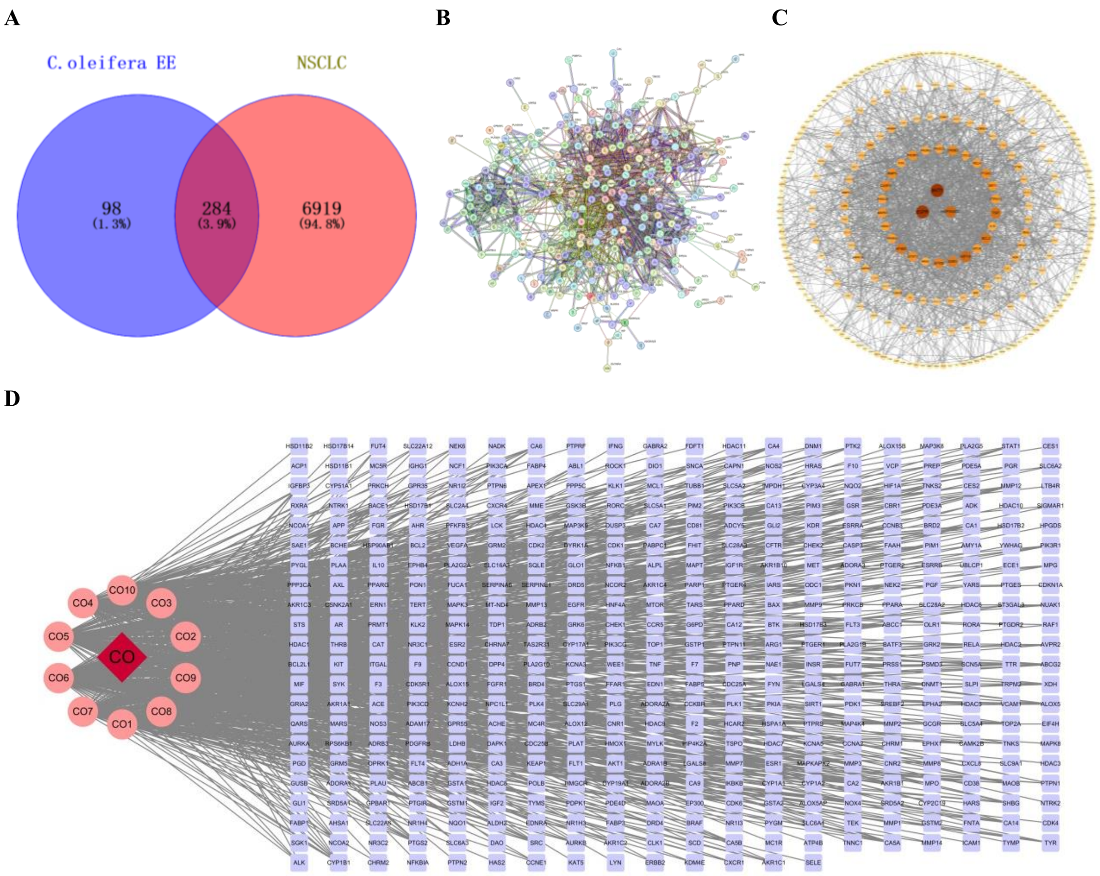

FIGURE S1 Network pharmacology of anti-NSCLC activity of *C. oleifera* bud EE. (A) Venn diagram of NSCLC-related genes and target genes of *C. oleifera* bud EE. (B) The PPI network diagram. (C) The PPI network diagram displayed in Cytoscape 3.9.1. (D) Network diagram of "*C. oleifera* bud EE-potential active compounds-target network". *C. oleifera* bud EE was expressed as CO. The major components in *C. oleifera* bud EE were as follows: astilbin (CO1), cianidanol (CO2), ellagic acid (CO3), hesperetin (CO4), isorhamnetin (CO5), kaempferol (CO6), licochalcone B (CO7), morin (CO8) procyanidin B1 (CO9), and *α*-boswellic acid (CO10).

2.2. GO and KEGG pathway enrichment analysis

GO and KEGG pathway enrichment analyses were also performed to elucidate the functions and enrichment pathways of potential anti-NSCLC genes targeted by *C. oleifera* bud EE. We extracted the top 20 significantly enriched terms from the three categories according to the counts and displayed by bar charts in Figure S2A. Analysis of biological process (BP) results showed that the targets of the compound *C. oleifera* bud EE involved in anti-NSCLC were significantly enriched in positive regulation of autophagy, electron transport chain, mitotic spindle organization, and other biological processes. Cellular component (CC) analysis highlighted the importance of cellular components such as endoplasmic reticulum, nuclear speck, early endosome, and other cellular components. Molecular function (MF) analysis results emphasized that protein C-terminus binding, chaperone binding, double-stranded DNA binding, and other molecular functions played important roles. KEGG pathway enrichment analysis revealed that 143 signaling pathways associated with the interaction between *C. oleifera* bud EE and NSCLC were statistically significant. These pathways were related to pathways in cancer, PI3K-Akt signaling pathway, chemical carcinogenesis-reactive oxygen species, etc. A visual representation in the form of a

bubble chart depicts the top 20 pathways with significant enrichment potential, along with the highest counts of associated genes (Figure S2B).

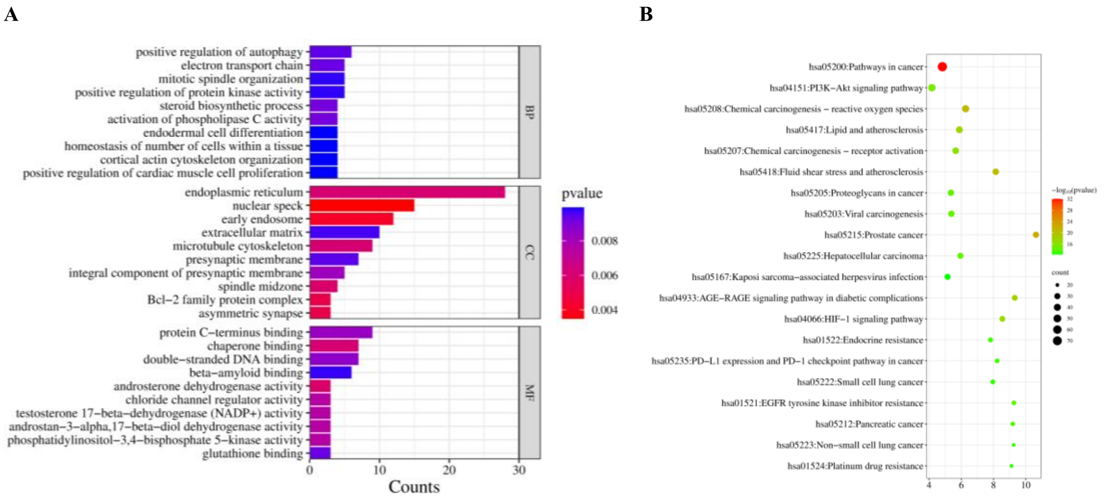

FIGURE S2 Analysis of potential targets of *C. oleifera* bud EE for anti-NSCLC based on GO enrichment (A) and KEGG enrichment (B).

2.3. Molecular docking results

Based on network pharmacology analysis results, molecular docking was performed on Autodock 4.2 to confirm the interaction of bioactive compounds and their core targets. As shown in Figure S3 and Table S1, the binding energies of AKT1 to ellagic acid, isorhamnetin, and kaempferol were -6, -6.1, and -6 kcal/mol, respectively. The binding energies of EGFR to these active components were as follows: -8 kcal/mol (ellagic acid), -8 kcal/mol (isorhamnetin), -8.1 kcal/mol (kaempferol), -7.3 kcal/mol (licochalcone B), -8 kcal/mol (morin), and -10.1 kcal/mol (procyanidin B1). Additionally, the binding energies of HSP90AB1 to cianidanol, ellagic acid, hesperetin, isorhamnetin, kaempferol, licochalcone B, morin, procyanidin B1 were -8.1, -8.7, -8.7, -8.8, -8.5, -7.7, -8.8 and -9.6 kcal/mol. Hence, the effects of *C. oleifera* bud EE on inducing apoptosis and inhibiting proliferation and metastasis of A549 cells may be related to the regulation of these three targets (AKT1, HSP90AB1, and EGFR) by these compounds.

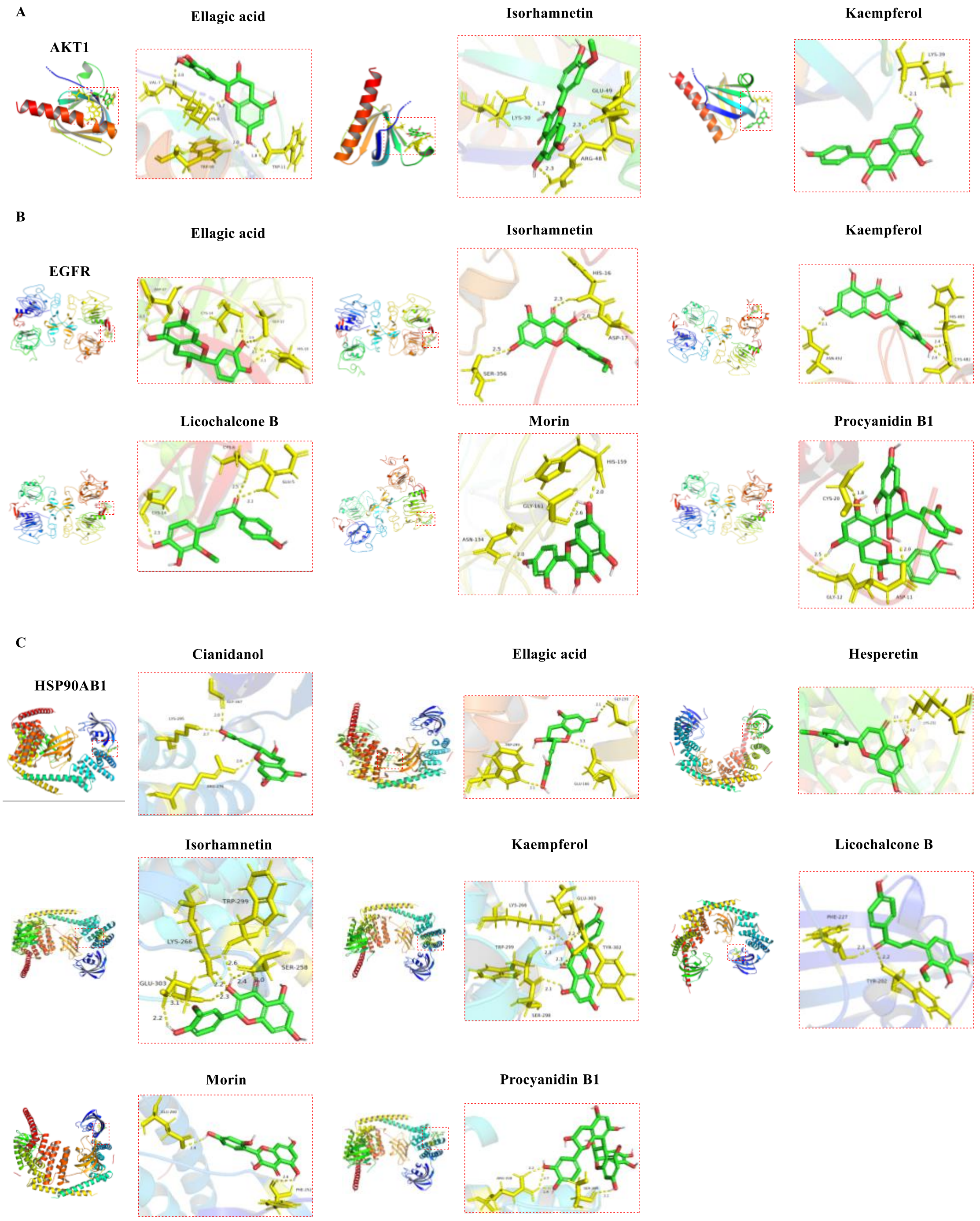

FIGURE S3 Molecular docking analysis showing the bond pattern. (A) The docking mode of AKT1 with corresponding components (ellagic acid, isorhamnetin, and kaempferol). (B) The docking mode of EGFR with related components (ellagic acid, isorhamnetin, kaempferol, licochalcone B, morin, and procyanidin B1). (C) The docking mode of HSP90AB1 with corresponding components (cianidanol, ellagic acid, hesperetin, isorhamnetin, kaempferol, licochalcone B, morin, and procyanidin B1).

TABLE S1 Molecular docking binding affinity of ligand with receptor (kcal/mol).

Receptor	PDB ID	Ligand	Binding affinity (kcal/mol)
AKT1	1H10	Ellagic acid	-6

	1H10	Isorhamnetin	-6.1
	1H10	Kaempferol	-6
EGFR	1IVO	Ellagic acid	-8
	1IVO	Isorhamnetin	-8
	1IVO	Kaempferol	-8.1
	1IVO	Licochalcone B	-7.3
	1IVO	Morin	-8
	1IVO	Procyanidin B1	-10.1
HSP90AB1	1QZ2	Cianidanol	-8.1
	1QZ2	Ellagic acid	-8.7
	1QZ2	Hesperetin	-8.7
	1QZ2	Isorhamnetin	-8.8
	1QZ2	Kaempferol	-8.5
	1QZ2	Licochalcone B	-7.7
	1QZ2	Morin	-8.8
	1QZ2	Procyanidin B1	-9.6

[1] γ-Aminobutyric acid (Wojnicz et al., 2016)

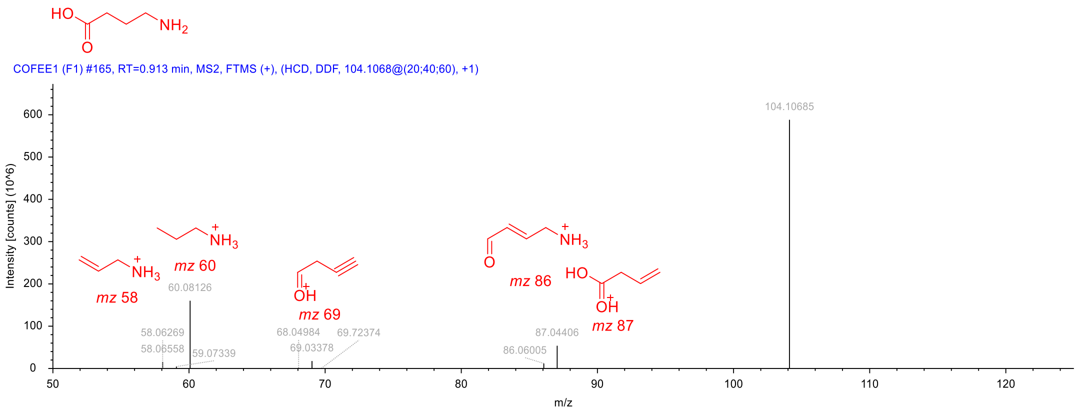

[2] Quinic acid (Deshpande et al., 2016)

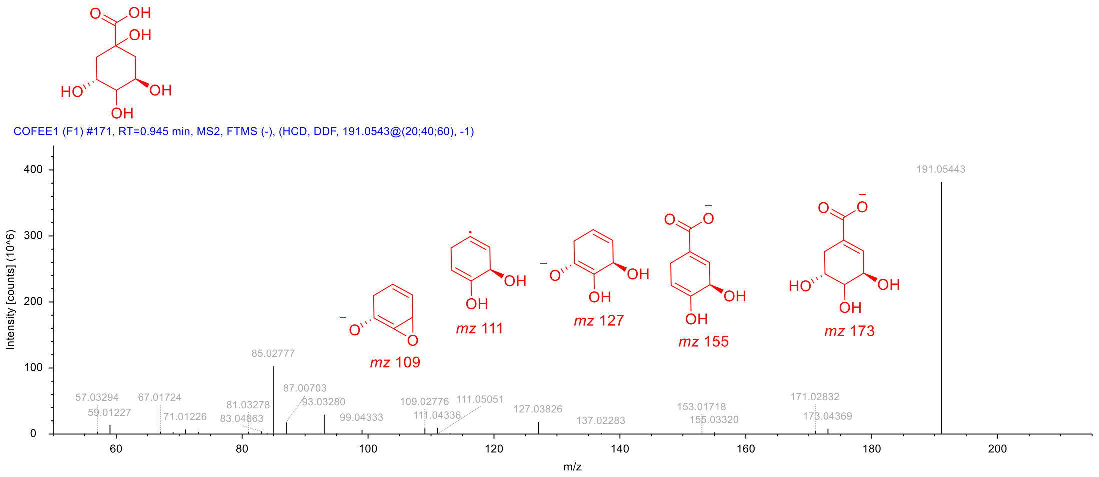

[3] 2-Pyrrolidinecarboxylic acid (Han et al., 2022)

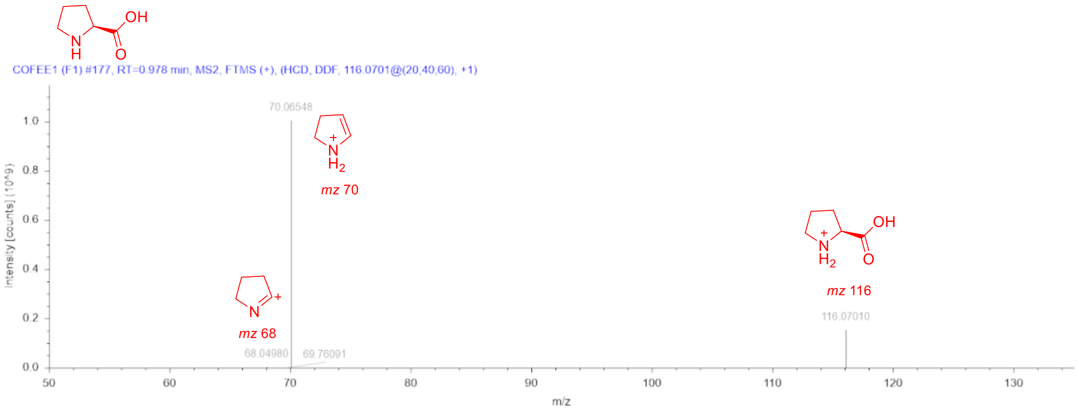

[4] Citric acid (Wang et al., 2019)

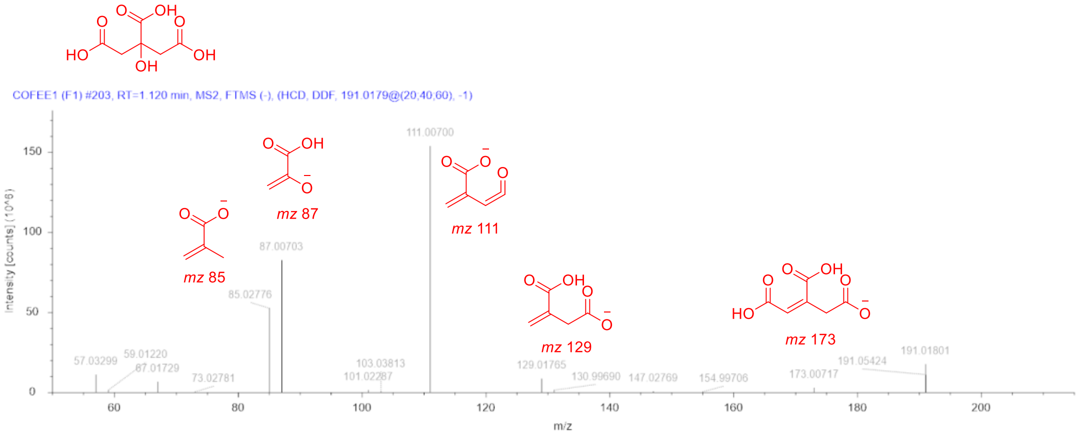

[5] *L*-Pyroglutamic acid

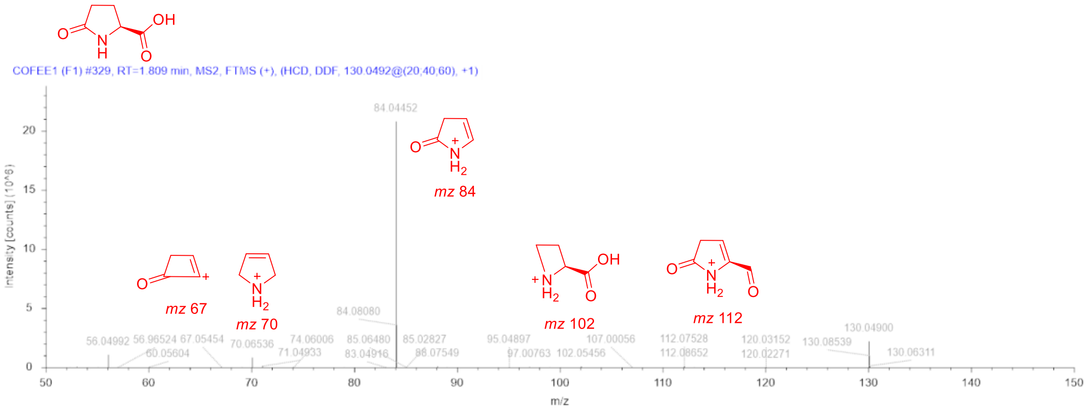

[6] *L*-Phenylalanine (Scheubert et al., 2013)

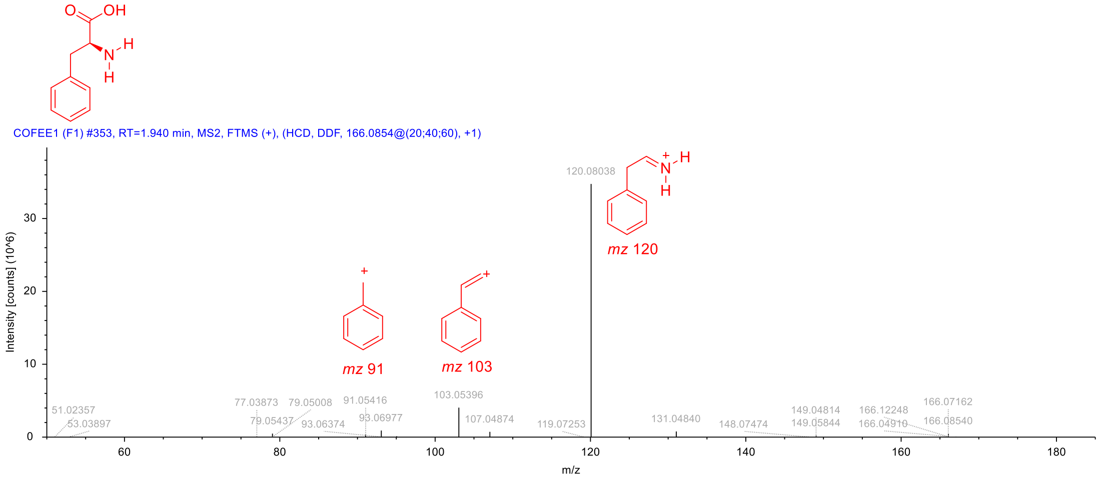

[7] *L*-Tyrosine (Zhao et al., 2006; Zheng et al., 2022)

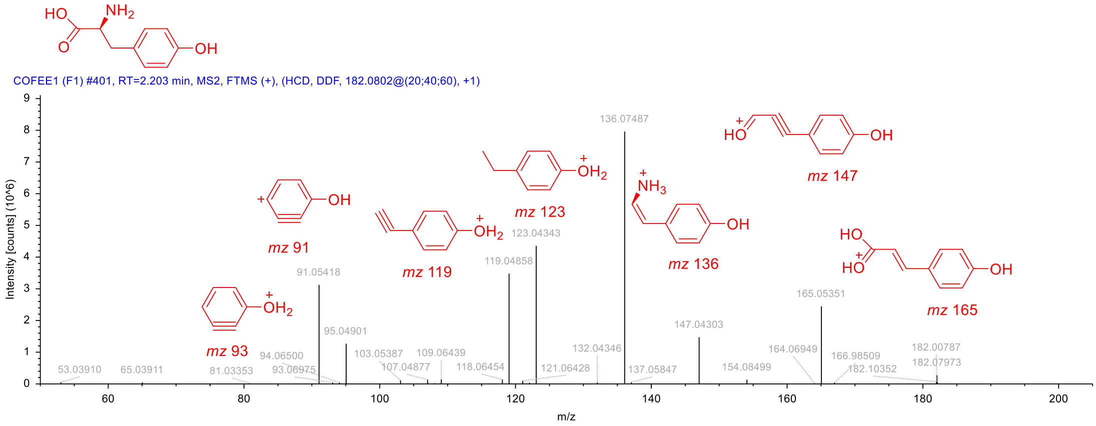

[8] *L*-Leucine (Jiang et al., 2020)

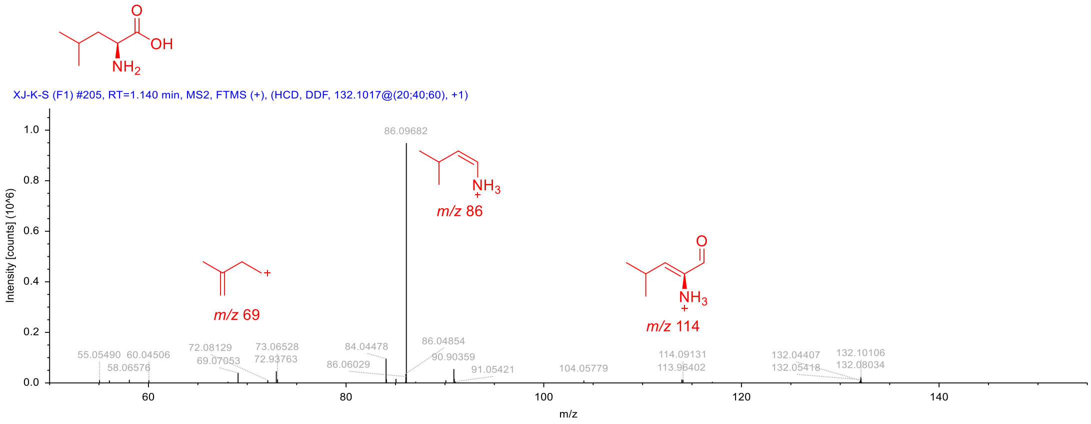

[9] Gallic acid (Shi et al., 2022)

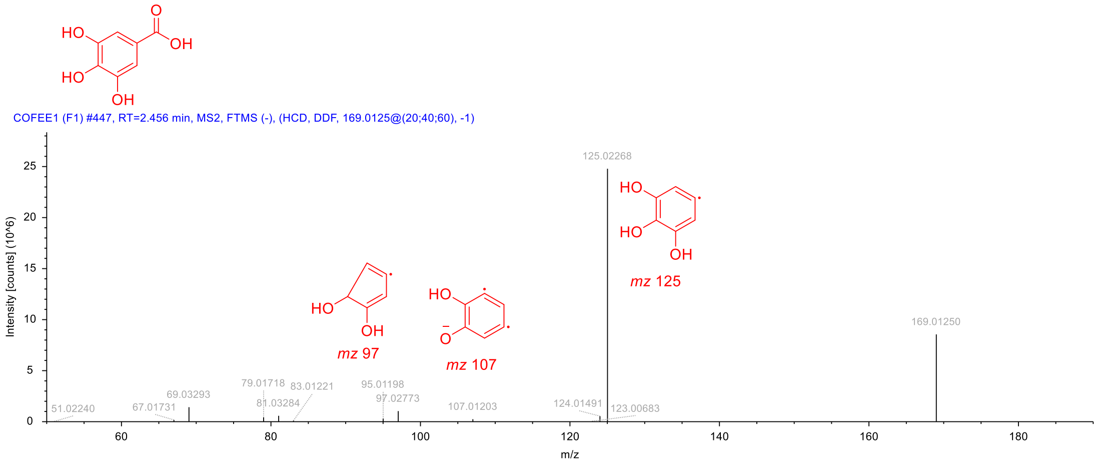

[10] Corilagin (Yisimayili et al., 2019)

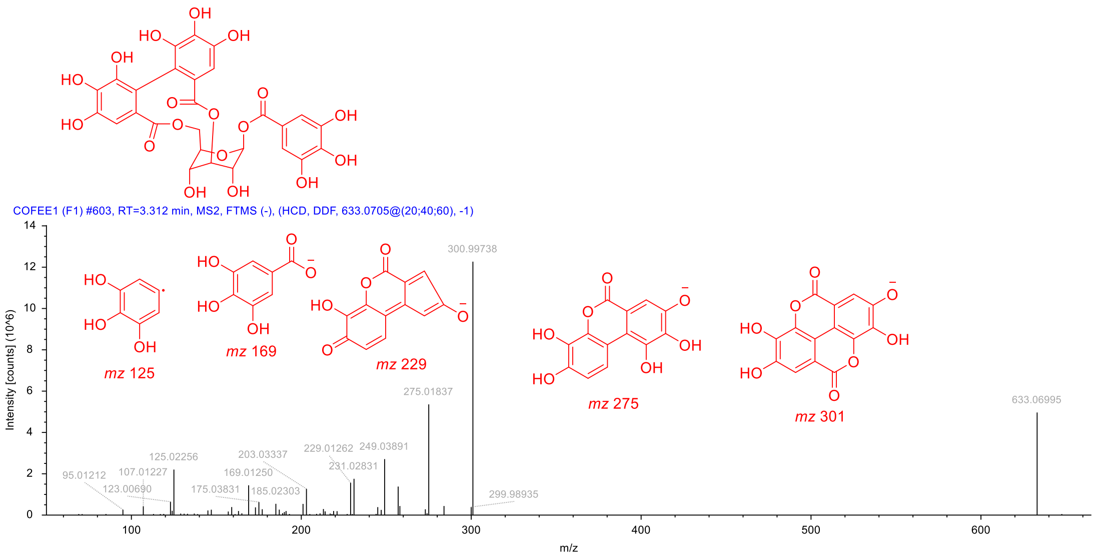

[11] Nicotinic acid (Stražić et al., 2014; Opitz et al., 2020)

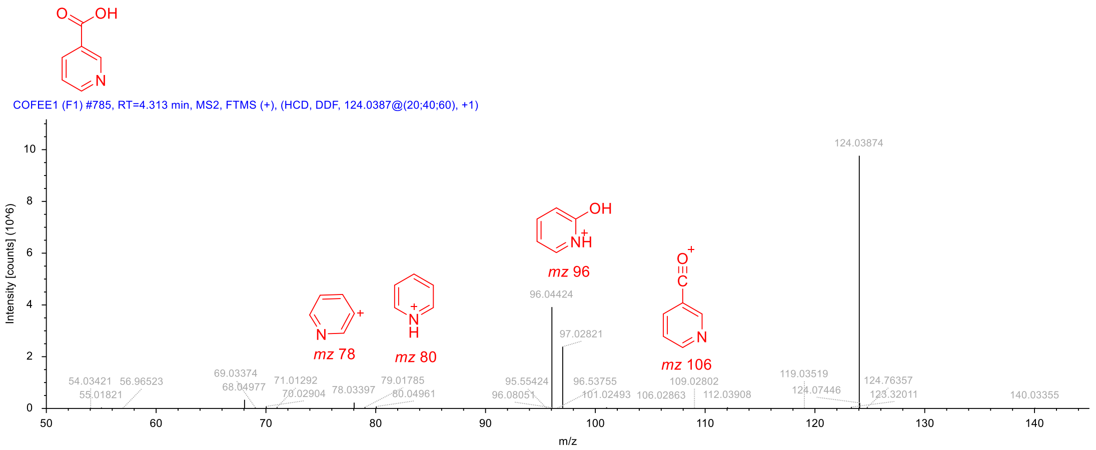

[12] *L*-Tryptophan (Zhang et al., 2019)

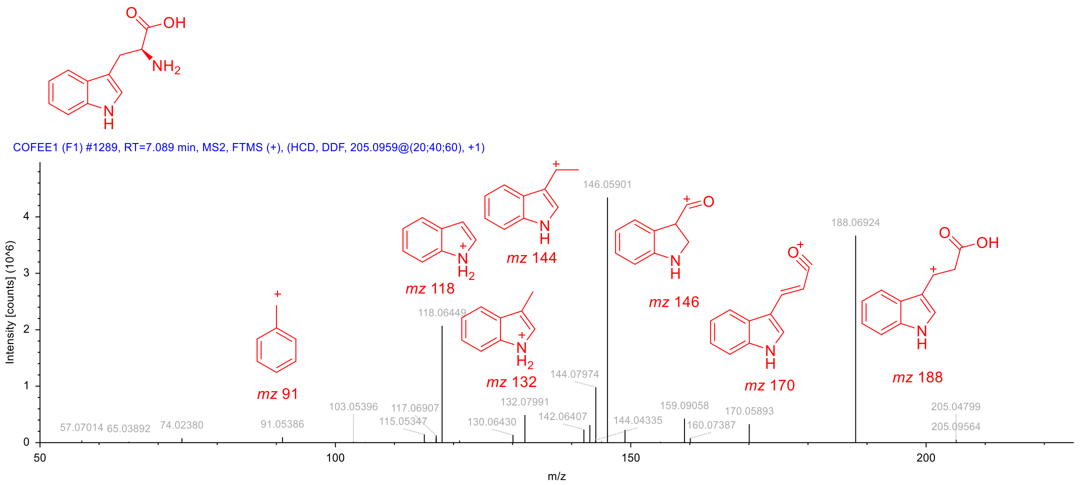

[13] 2-Isopropylmalic acid (Menicatti et al., 2020)

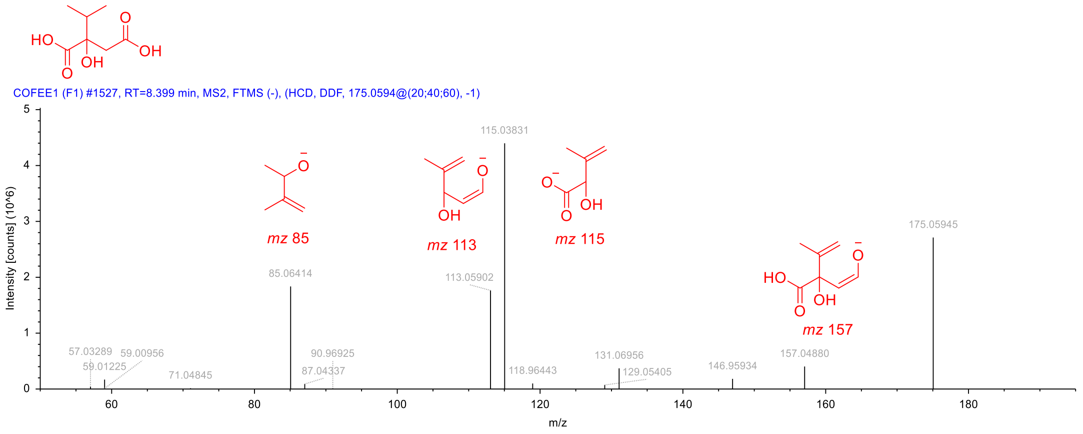

[14] Procyanidin B1 (Heger et al., 2022; Weinert et al., 2012; Callemien and Collin, 2008)

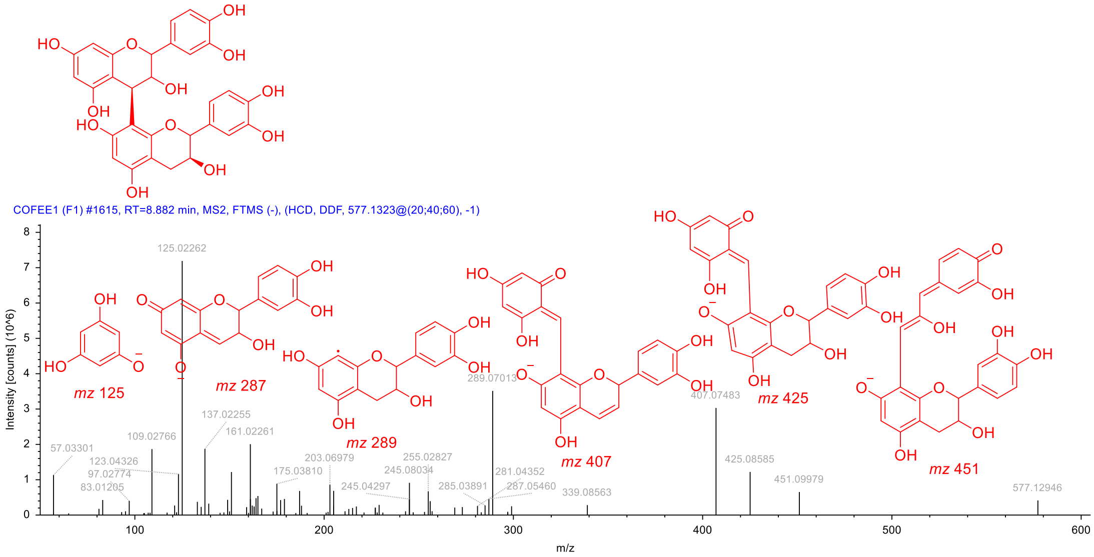

[15] Epicatechin (Wolf et al., 2010)

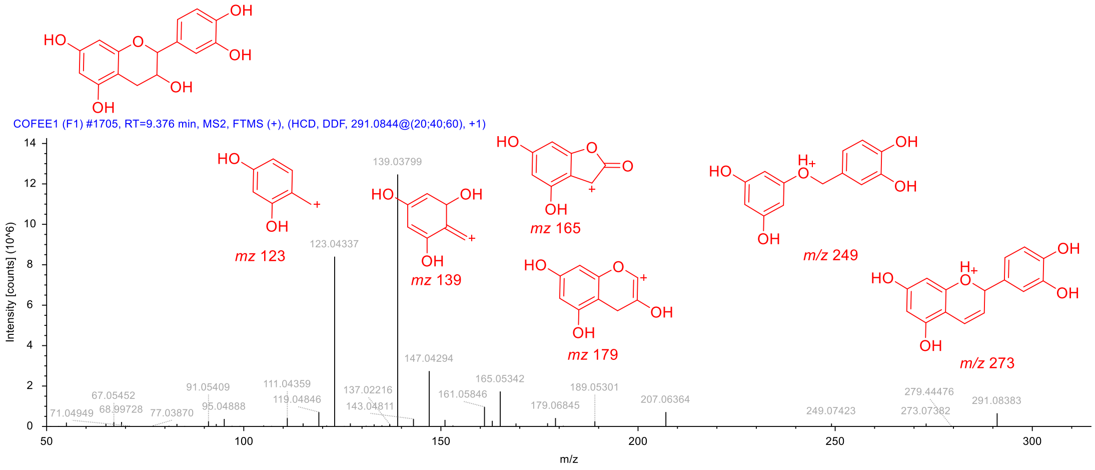

[16] (+)-Catechin hydrate (Xu et al., 2021)

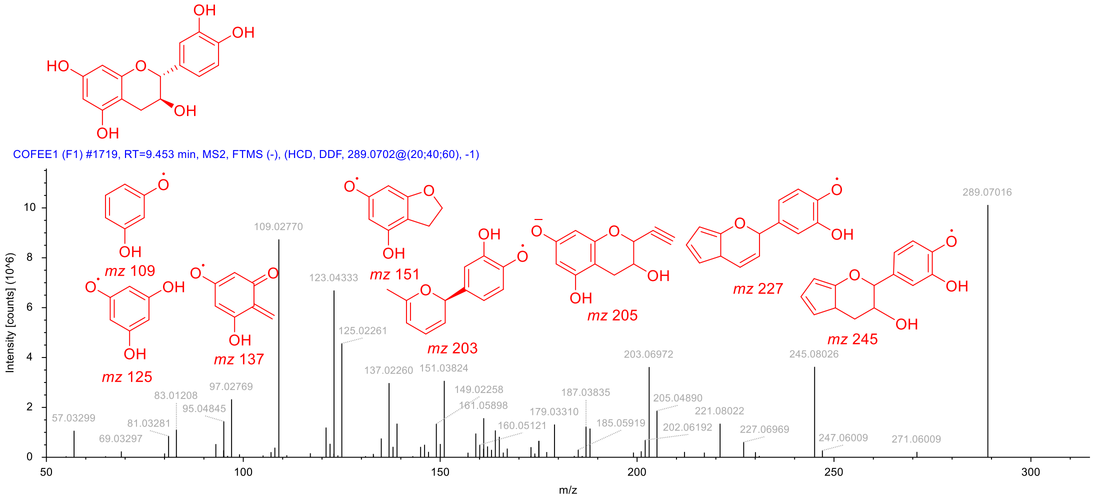

[17] Procyanidin B2 (Enomoto and Nirasawa, 2020; Xiao et al., 2017a)

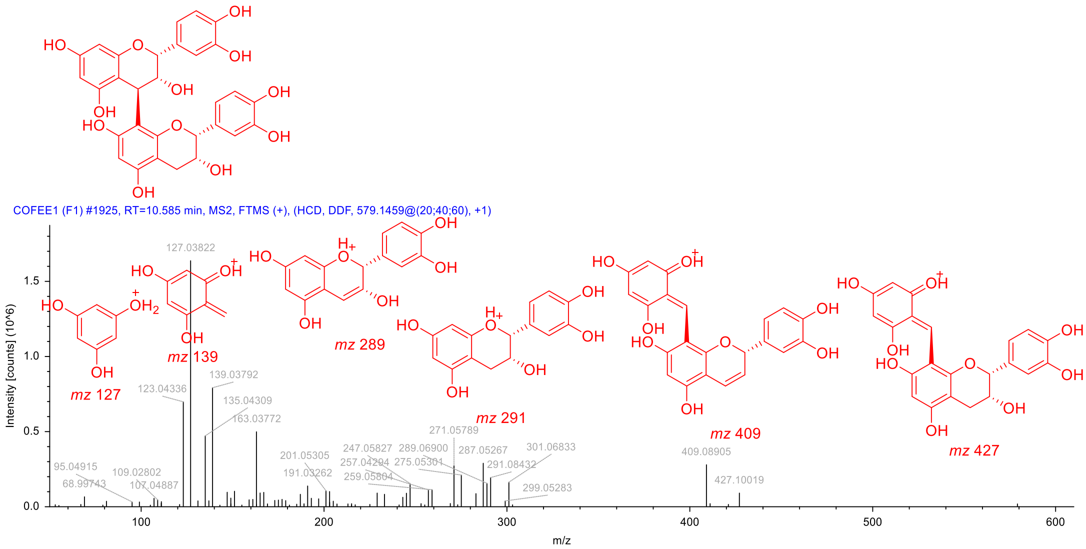

[18] Cianidanol (Tao, 2021)

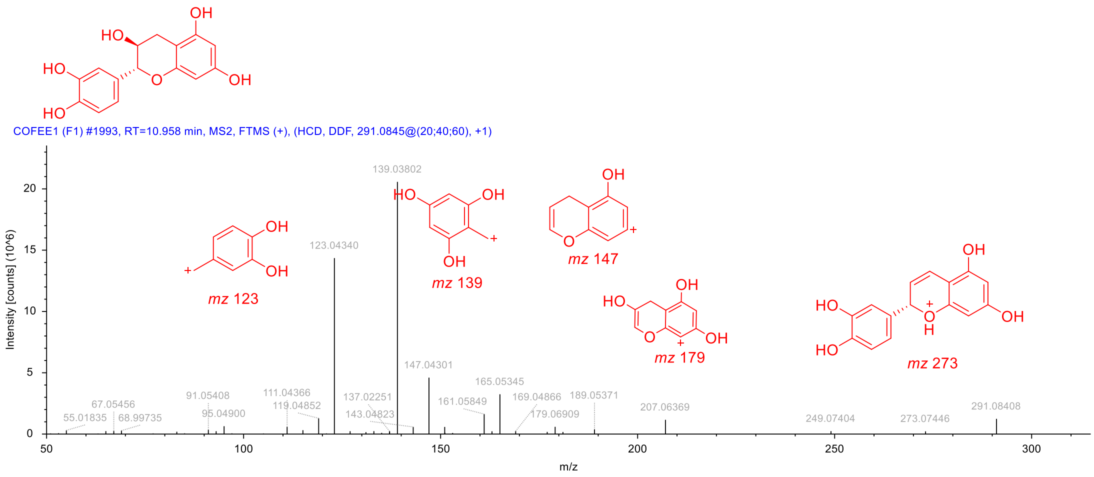

[19] Benzoic acid (Liang et al., 2022; Jia et al., 2023)

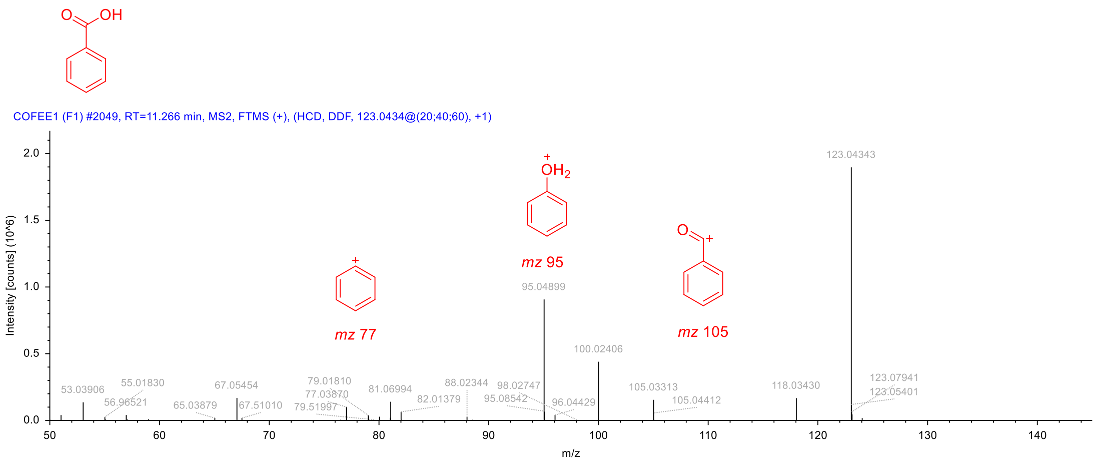

[20] 2-Hydroxy-4-methoxybenzaldehyde (Ding et al., 2019)

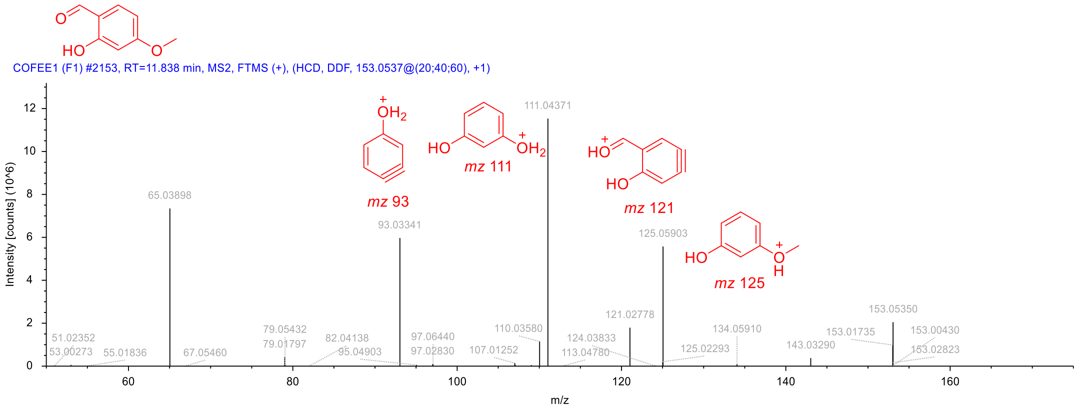

[21] Ethyl gallate (Shahzad et al., 2022; Zhang et al., 2018)

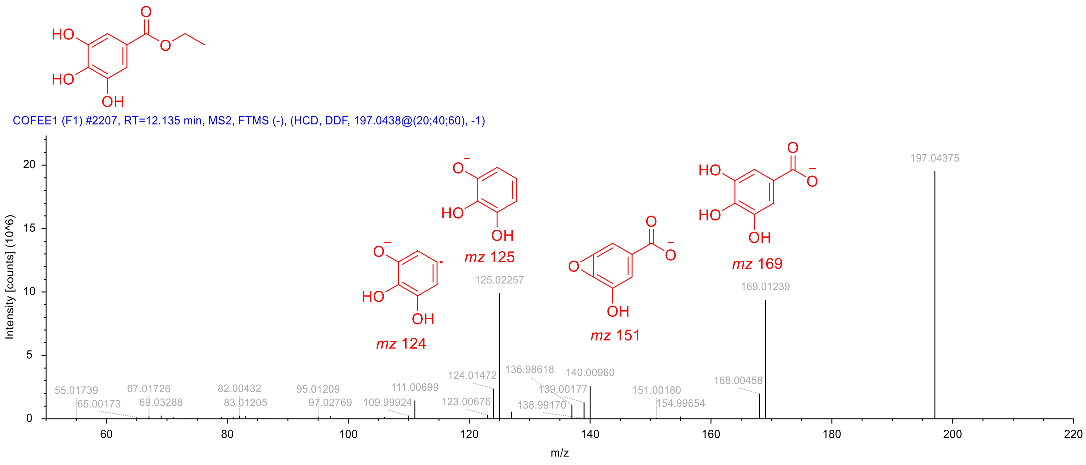

[22] 3, 5-Dimethoxy-4-hydroxybenzaldehyde (Wu et al., 2019)

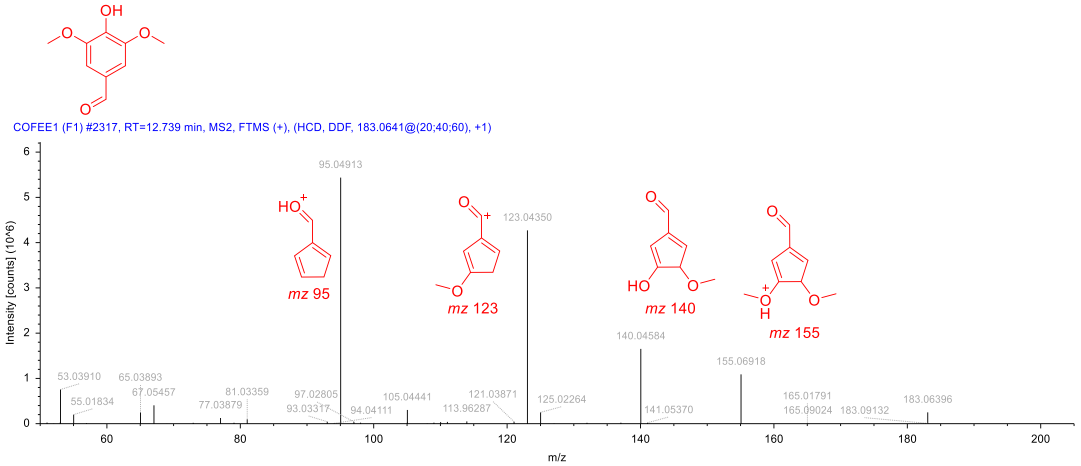

[23] 2'-*O*-Galloylhyperin (Ji et al., 2022)

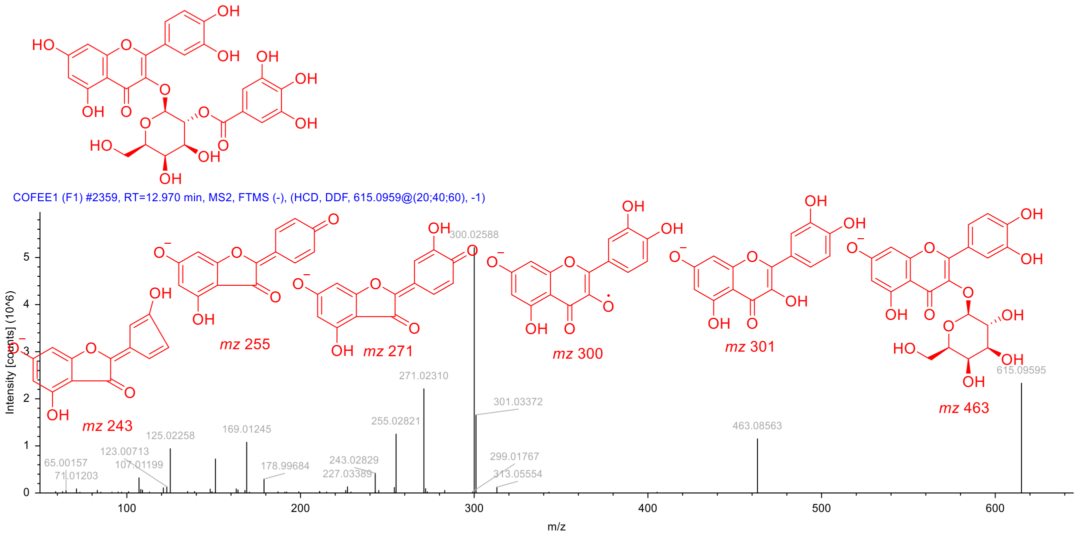

[24] Isorhamnetin (Justesen 2020; Sriseadka et al., 2012; Fabre et al., 2001)

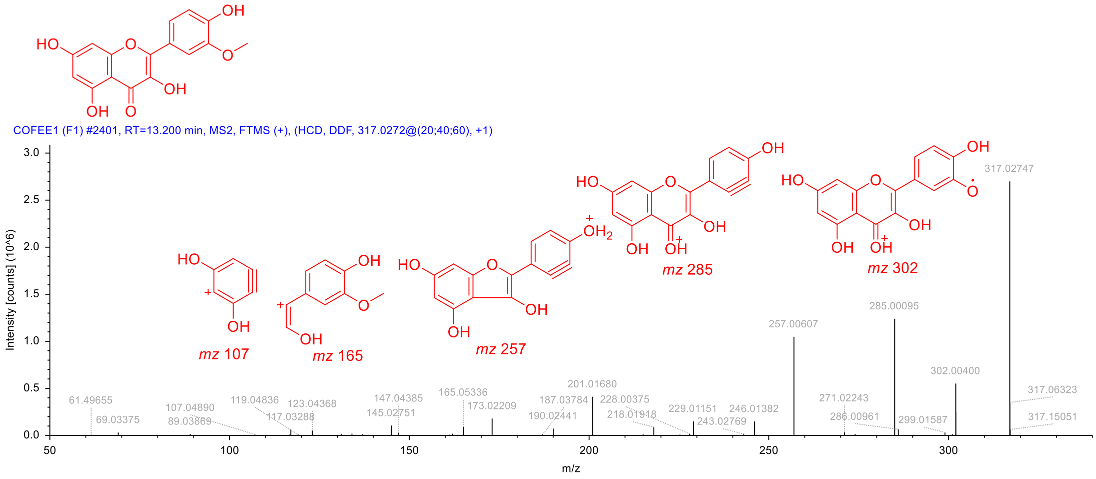

[25] Dihydroresveratrol (Wang et al., 2020)

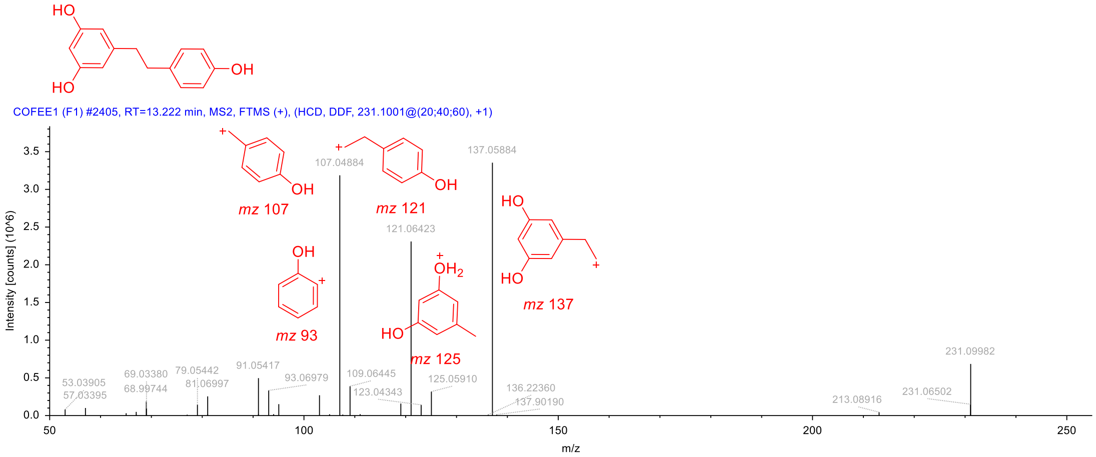

[26] Ellagic acid (Li et al., 2022)

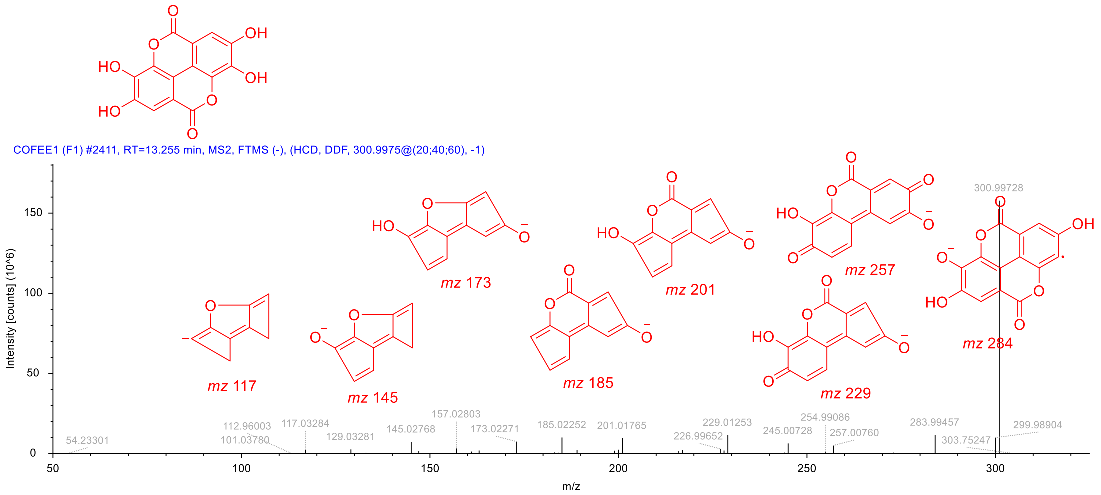

[27] (-)-Epicatechin gallate (Castro et al., 2020; Li et al., 2021)

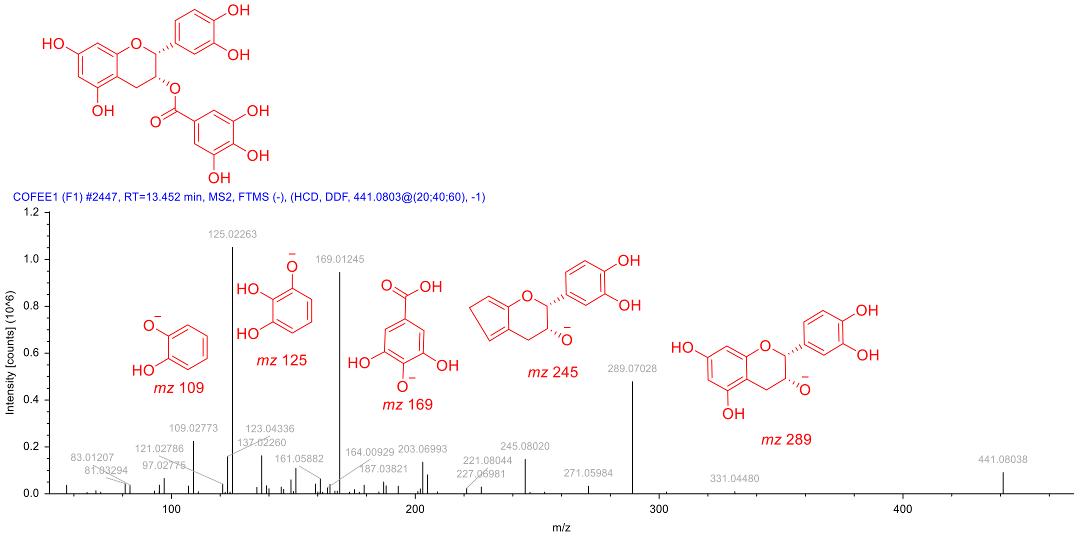

[28] Isoquercitrin (Wang, 2015)

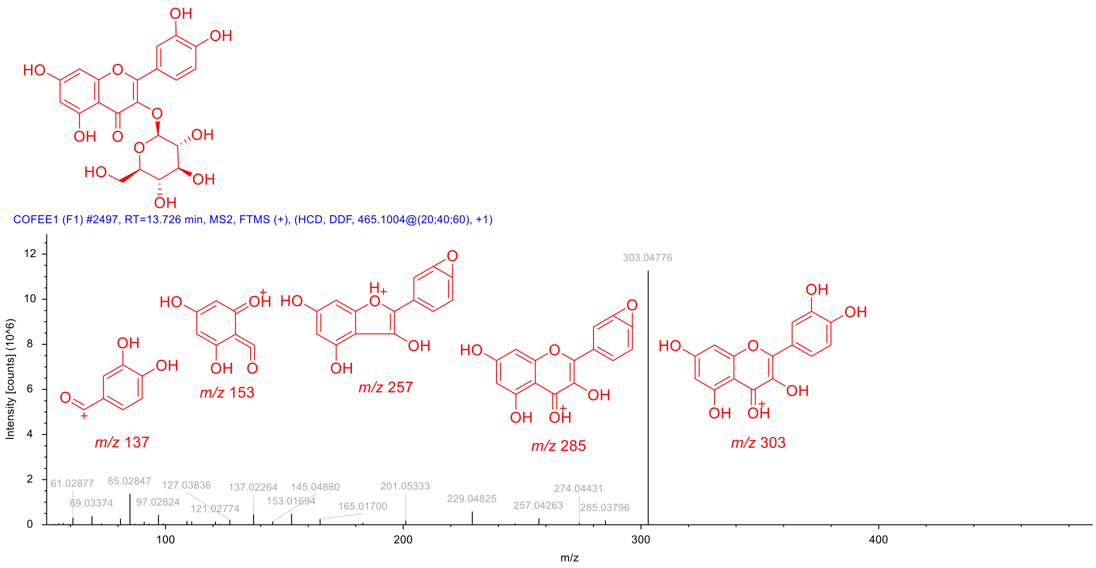

[29] Astilbin (de Brito et al., 2021)

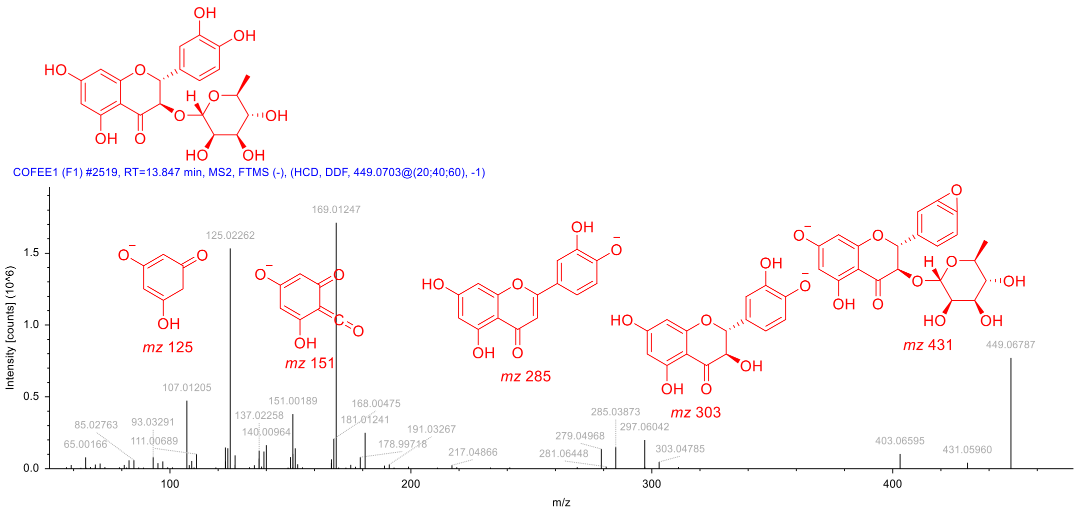

[30] 1,2,3,4,6-Pentagalloylglucose (Sun et al., 2023)

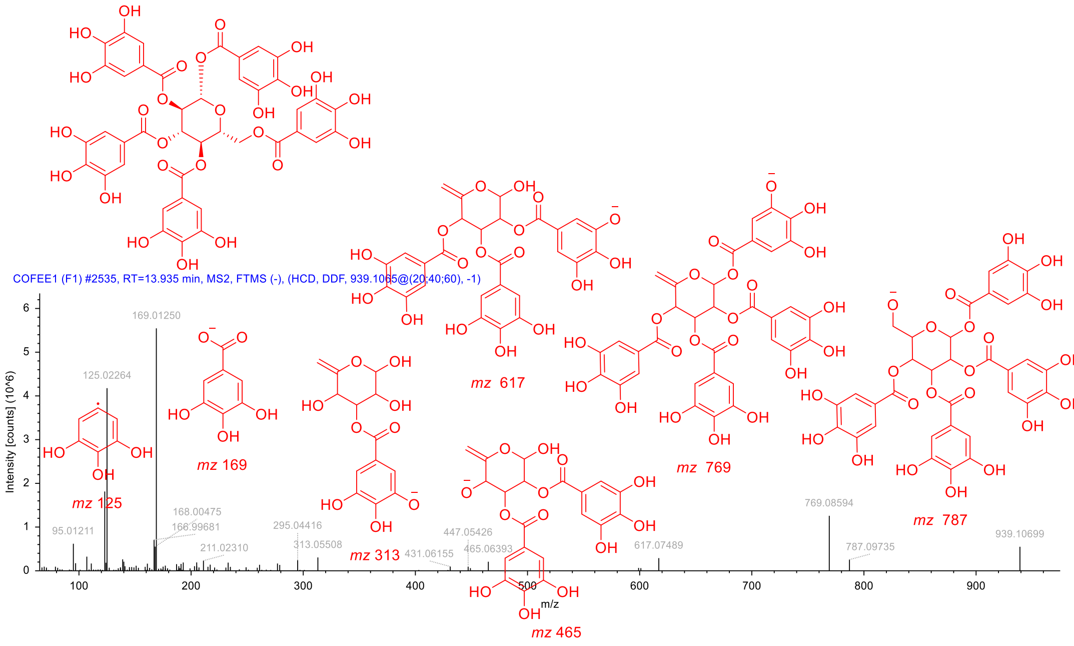

[31] Astragalin (Wu et al., 2023; Xiao et al., 2017b)

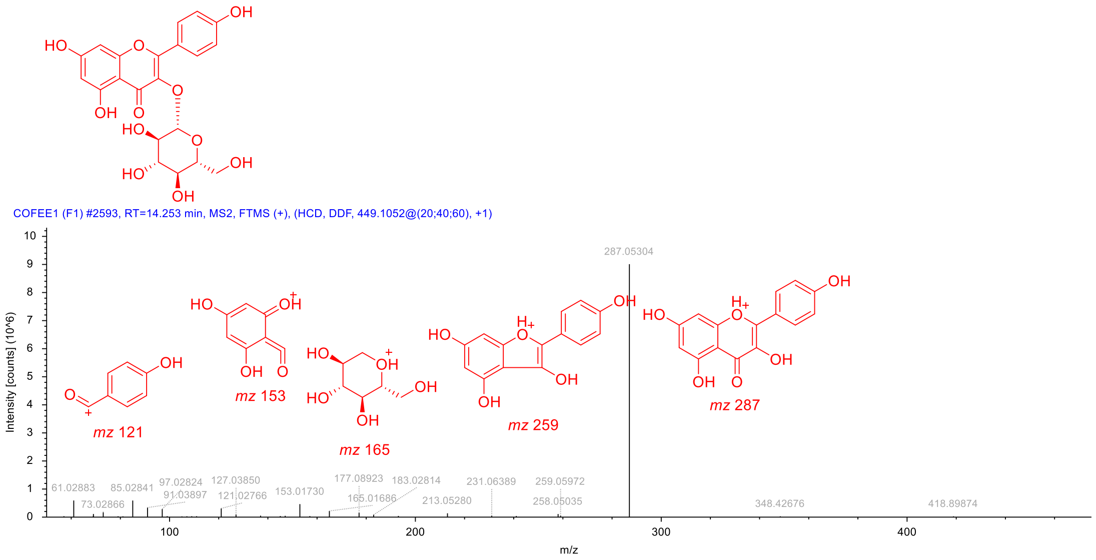

[32] Kaempferol (March and Miao, 2004)

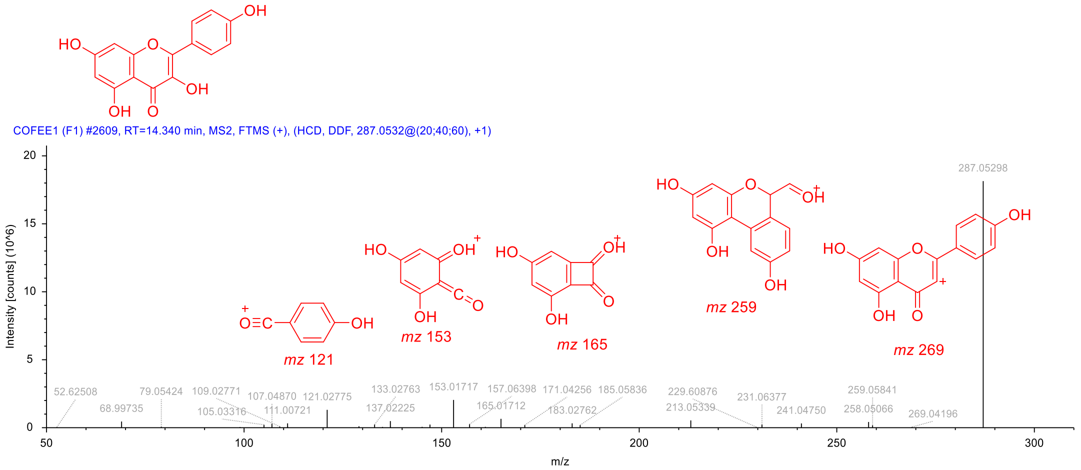

[33] Isosakuranetin (He et al., 2021)

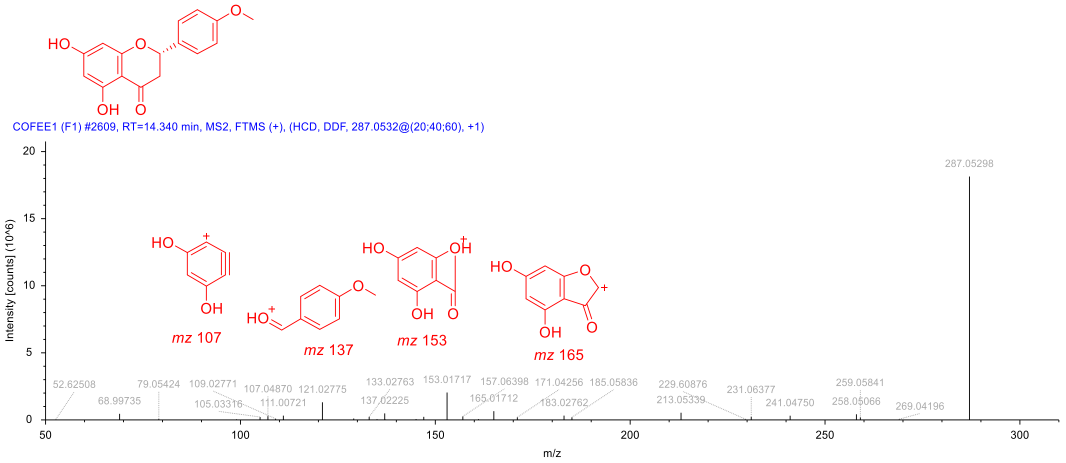

[34] Trilobatin (Dug éde Bernonville et al., 2010)

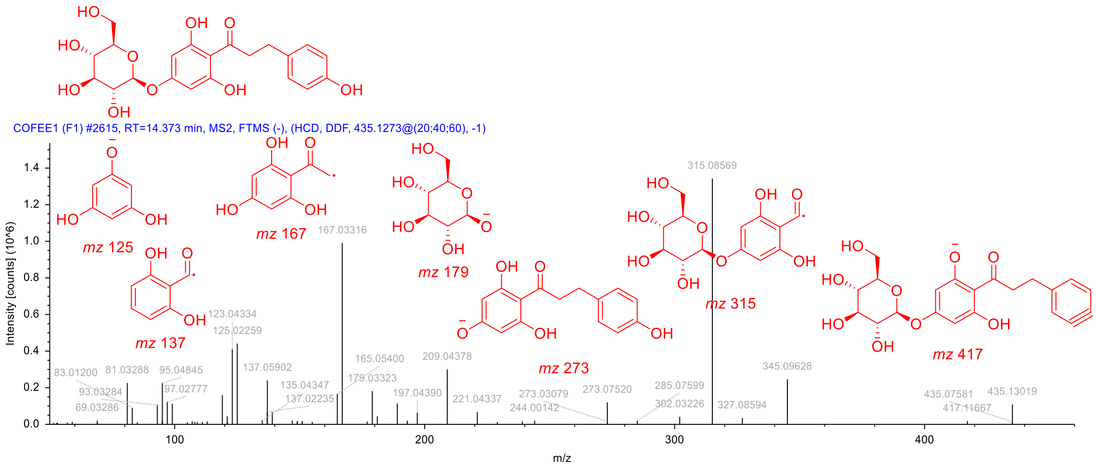

[35] Hesperetin (Wang et al., 2021; Jiao et al., 2020)

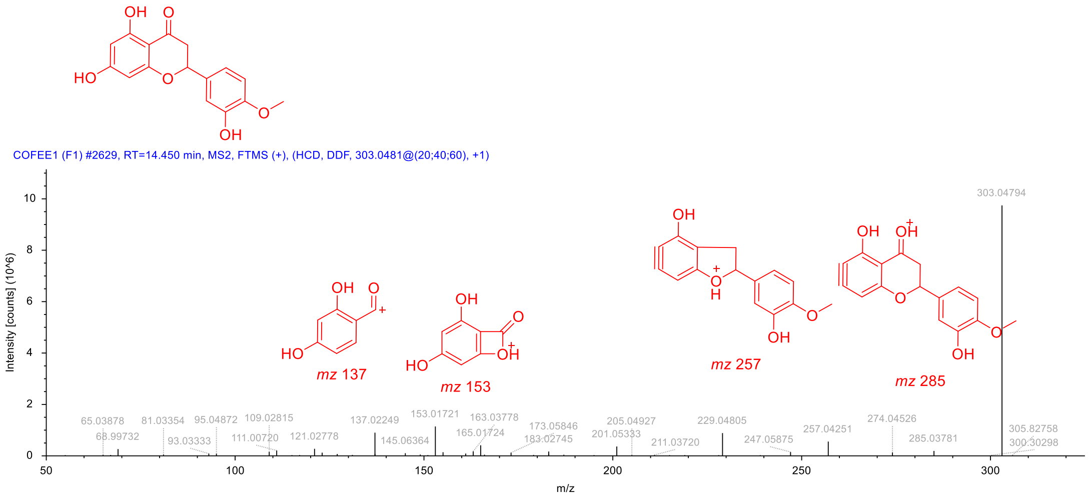

[36] Ferulaldehyde (Liang et al., 2022)

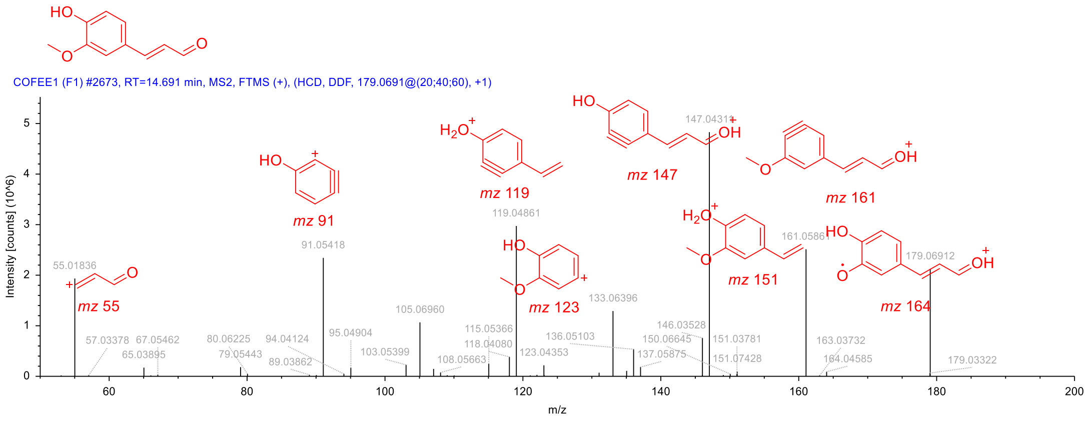

[37] 7-Methoxycoumarin (Concannon et al., 2000; Kutney et al., 1971)

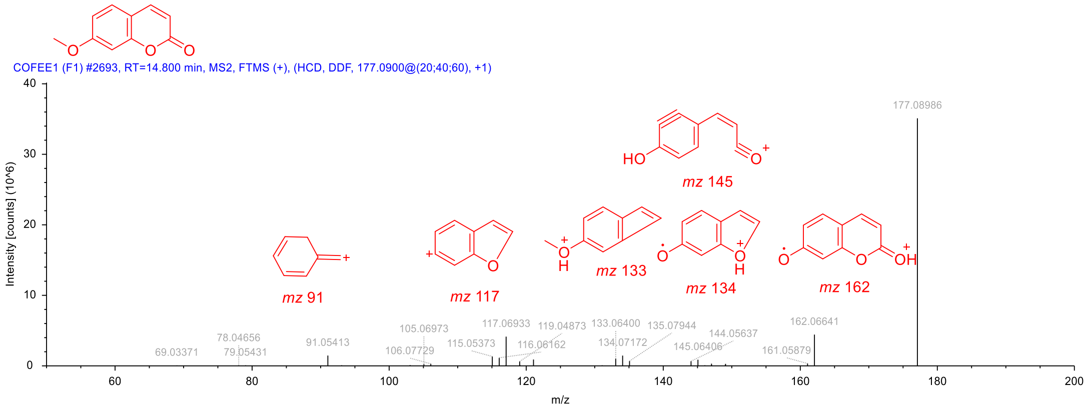

[38] Quercitrin (Dubber et al., 2005; da Costa, et al., 2016)

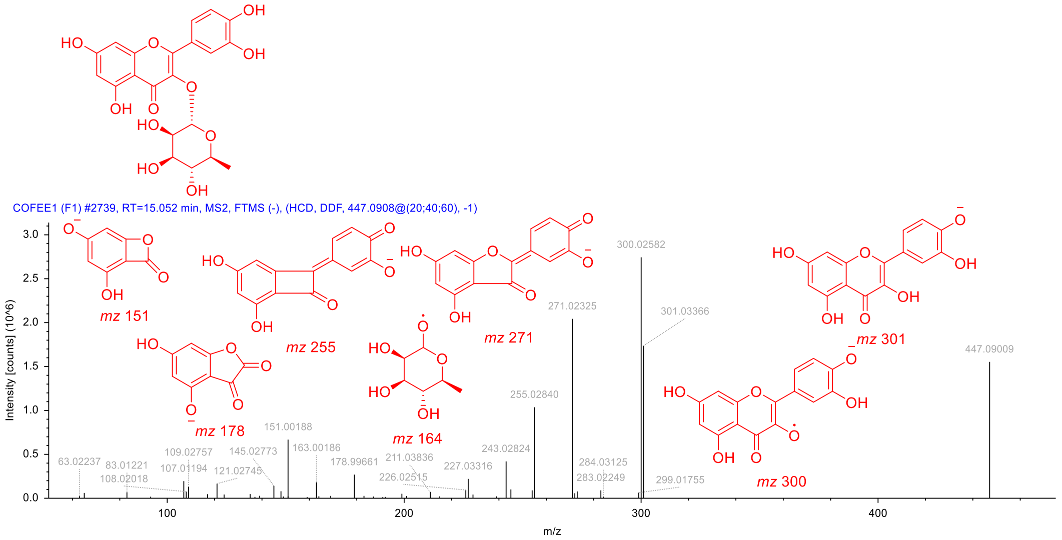

[39] Ailanthone (Chuang et al., 2022)

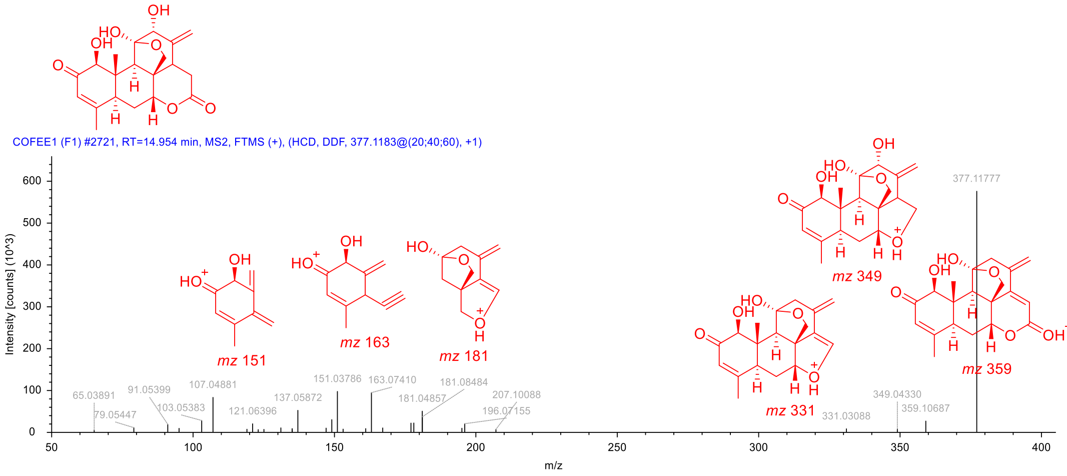

[40] Azelaic acid (Abu-Reidah et al., 2013)

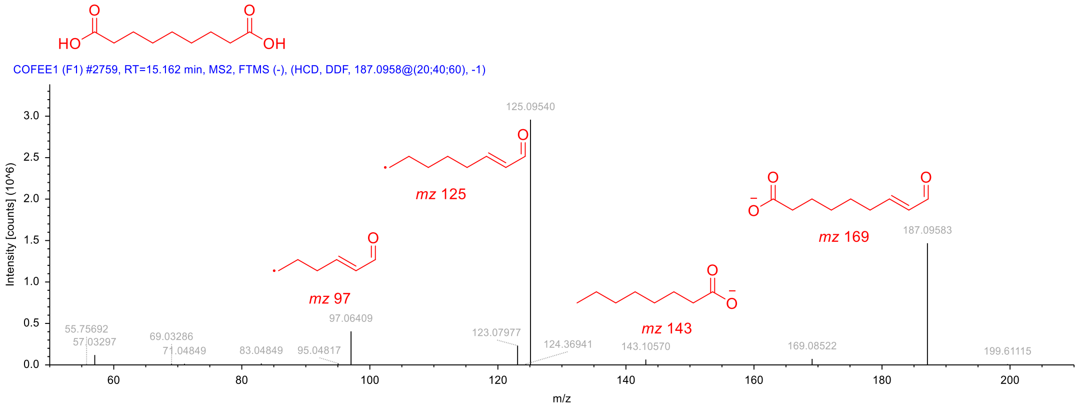

[41] Sinapyl aldehyde

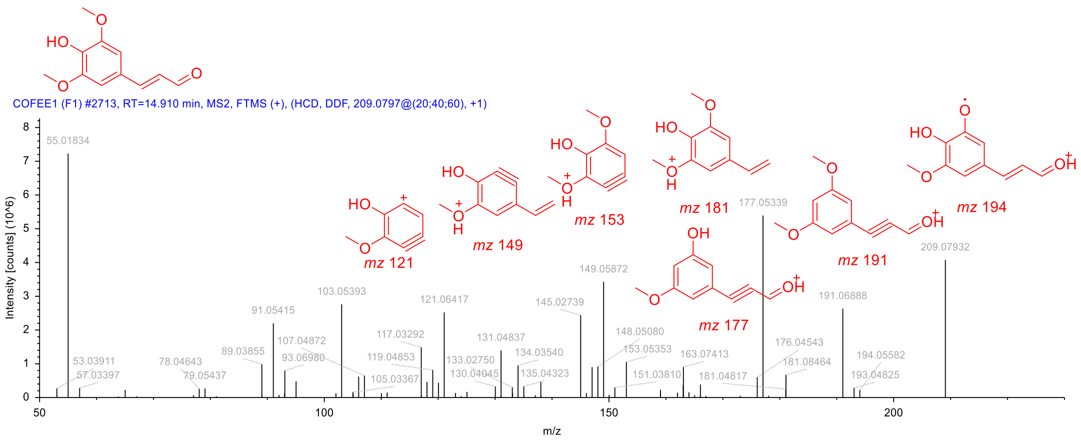

[42] Phloridzin (Lu et al., 2022)

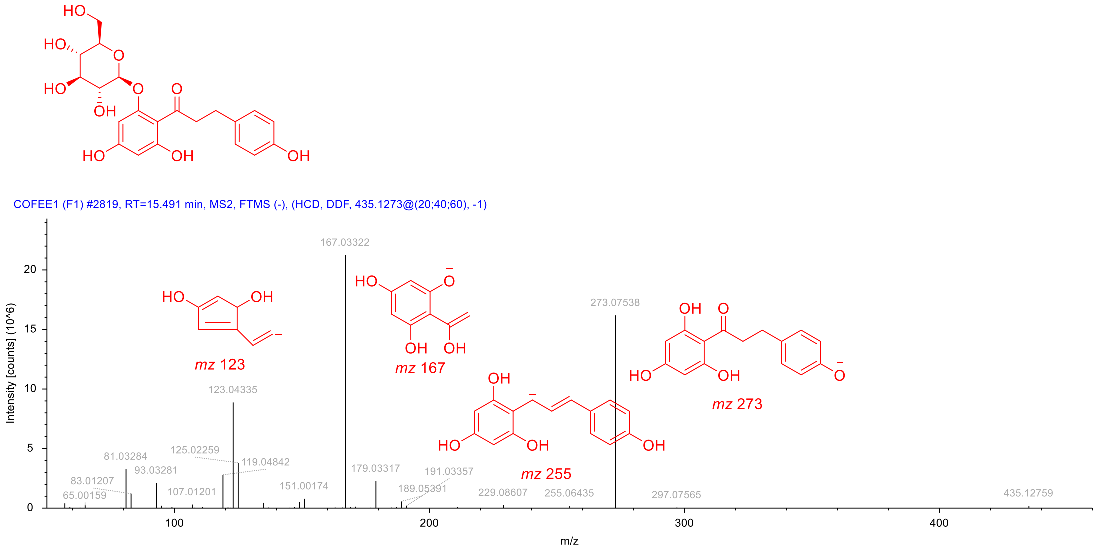

[43] Orsellinic acid (Ma et al., 2016)

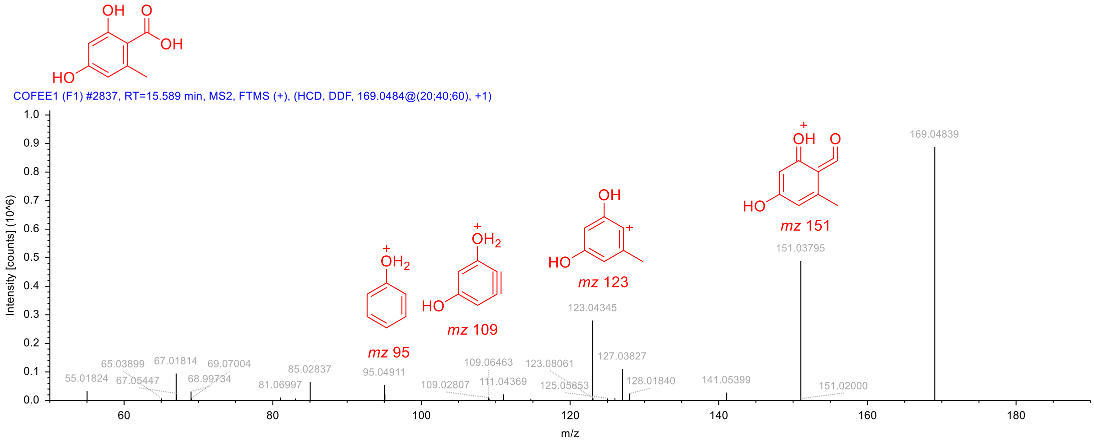

[44] α-Cyperone (Syahbirin et al., 2020)

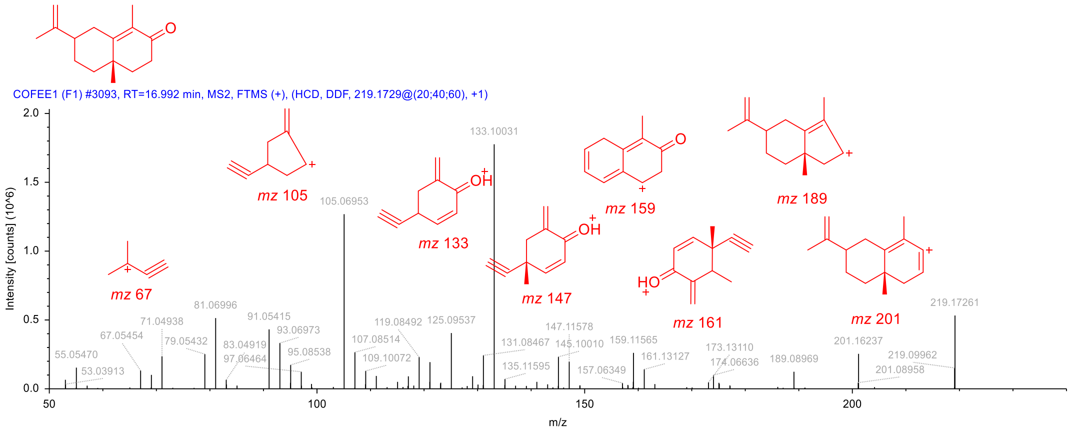

[45] Licochalcone B (Sun et al., 2022)

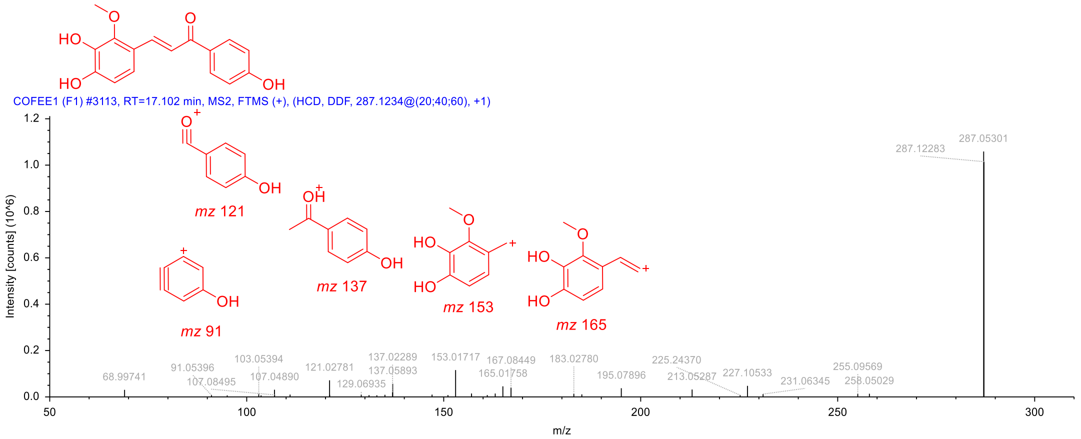

[46] Morin (Fabre et al., 2001)

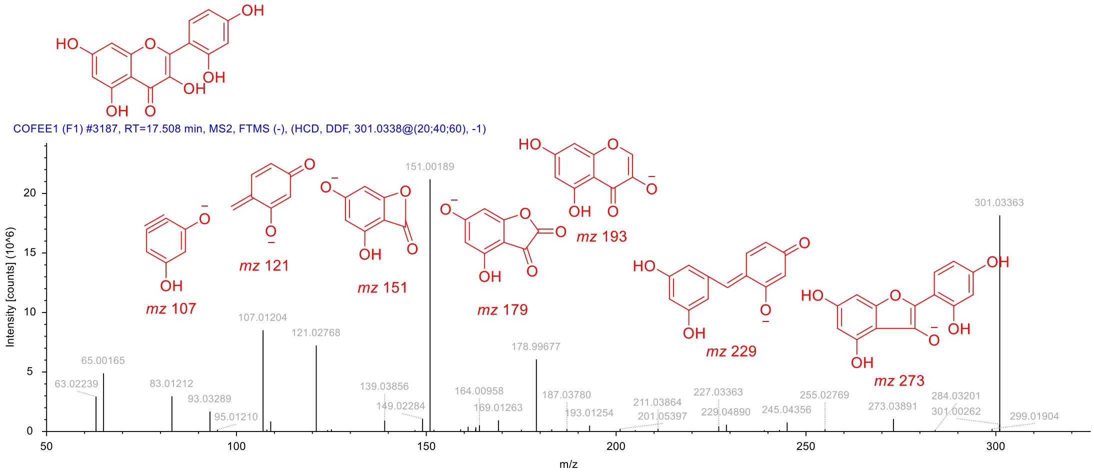

[47] *o*-Veratraldehyde (Wang et al., 2021)

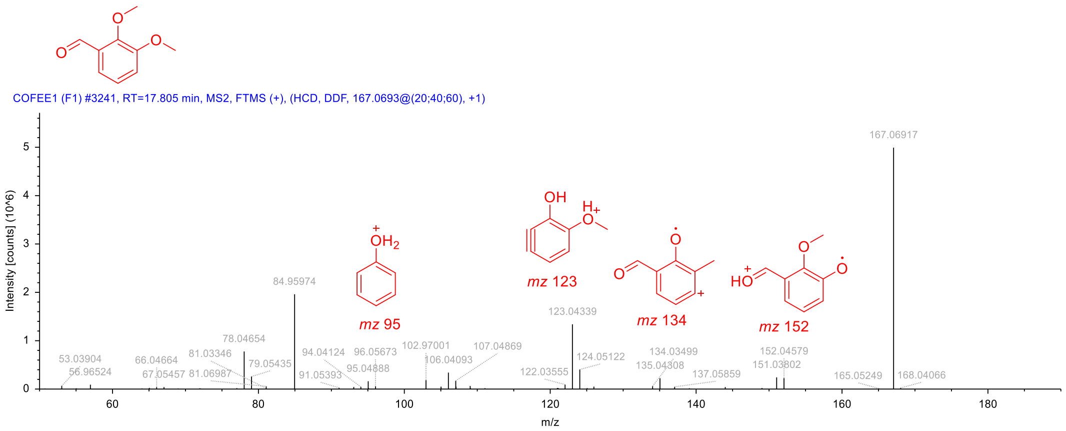

[48] Atractyloside A

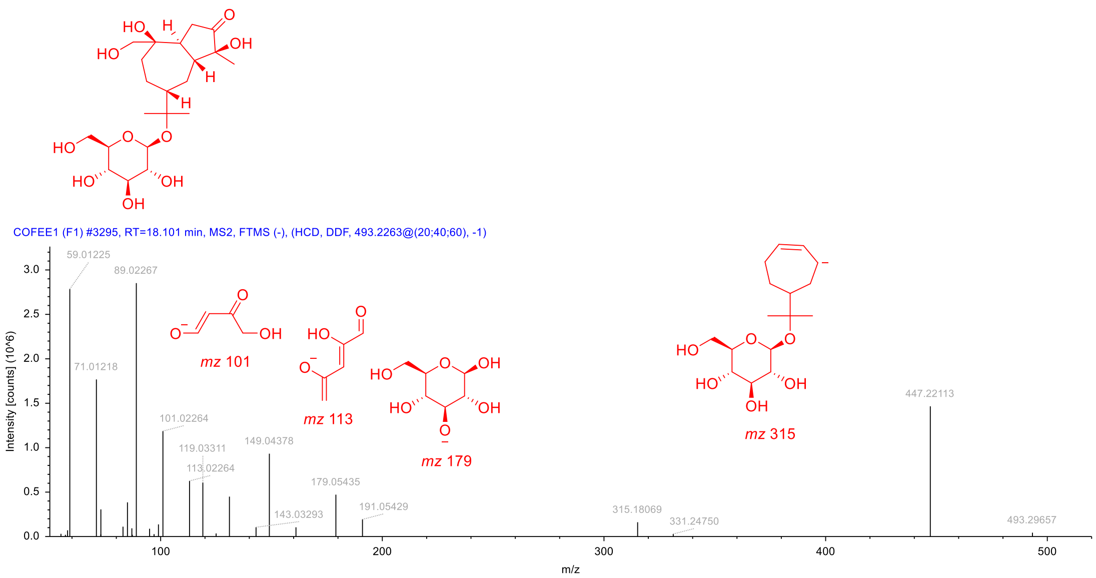

[49] Phloretin (Wang et al., 2020)

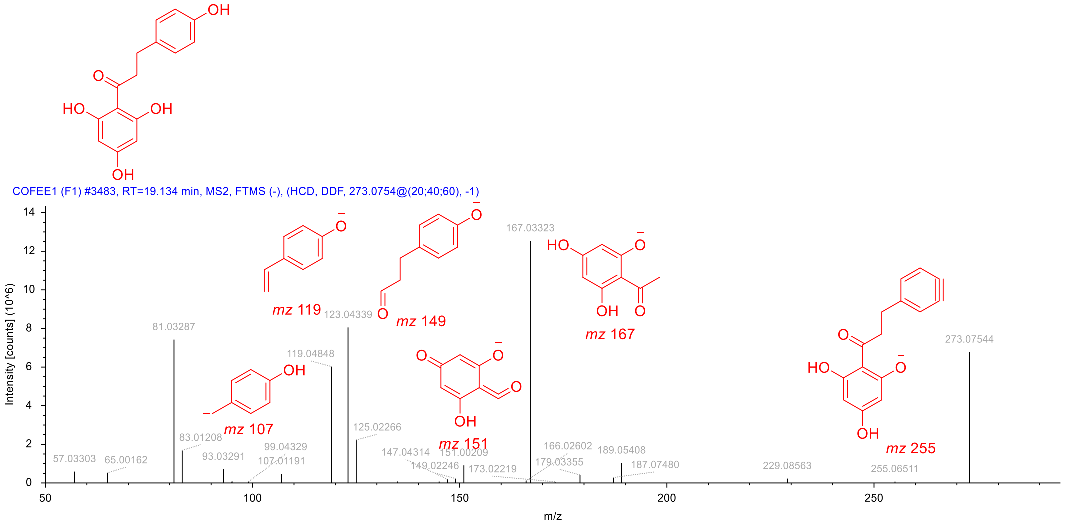

[50] Astringin (Moss et al., 2013)

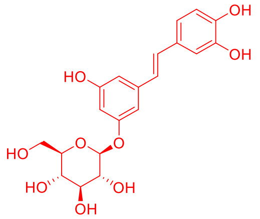

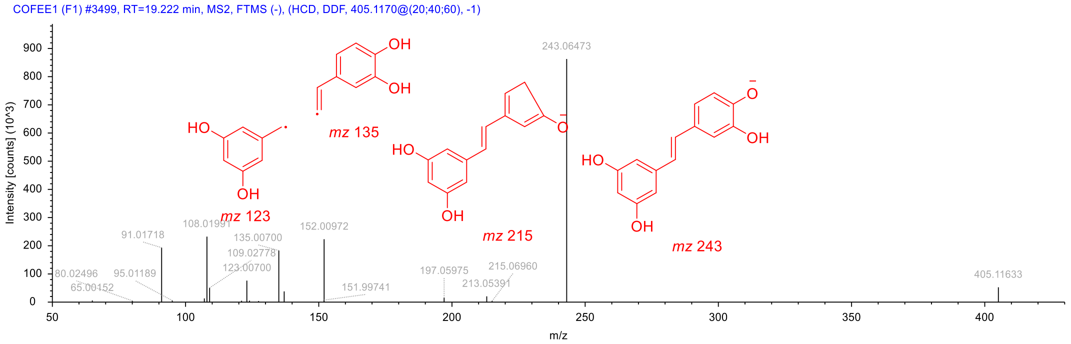

[51] Eupafolin (Su, 2023)

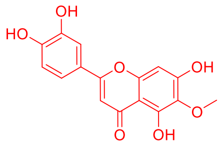

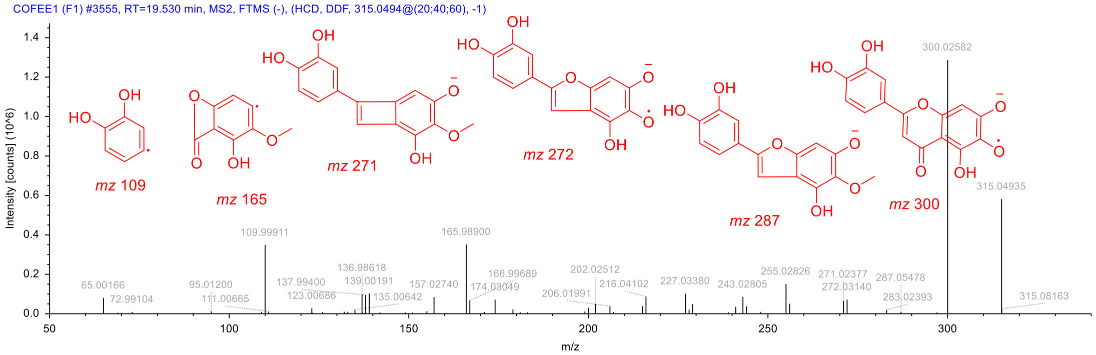

[52] Cinnamaldehyde (Flamini et al., 2007)

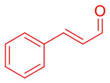

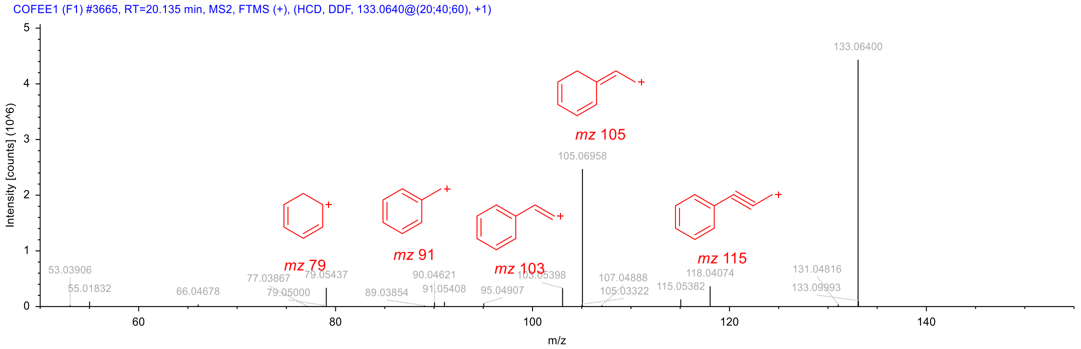

[53] Sauchinone

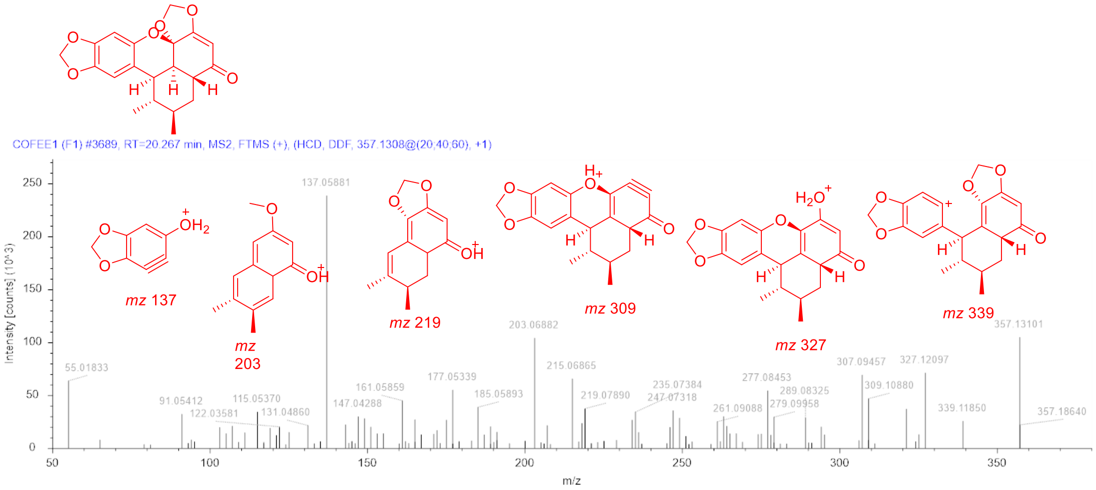

[54] Dichotomitin (Wei et al., 2012; Zhang et al., 2011)

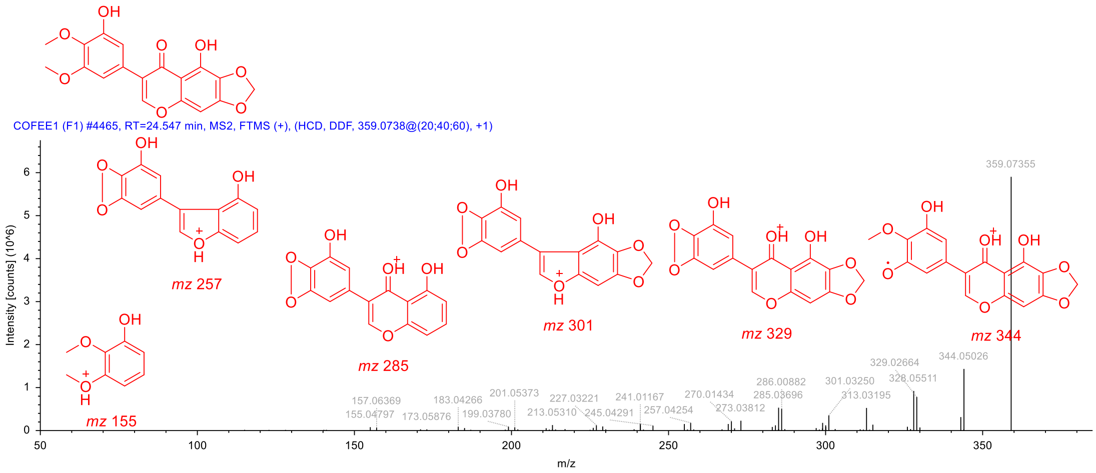

[55] Medicagenic acid (Biazzi et al., 2015; Peeters et al., 2020)

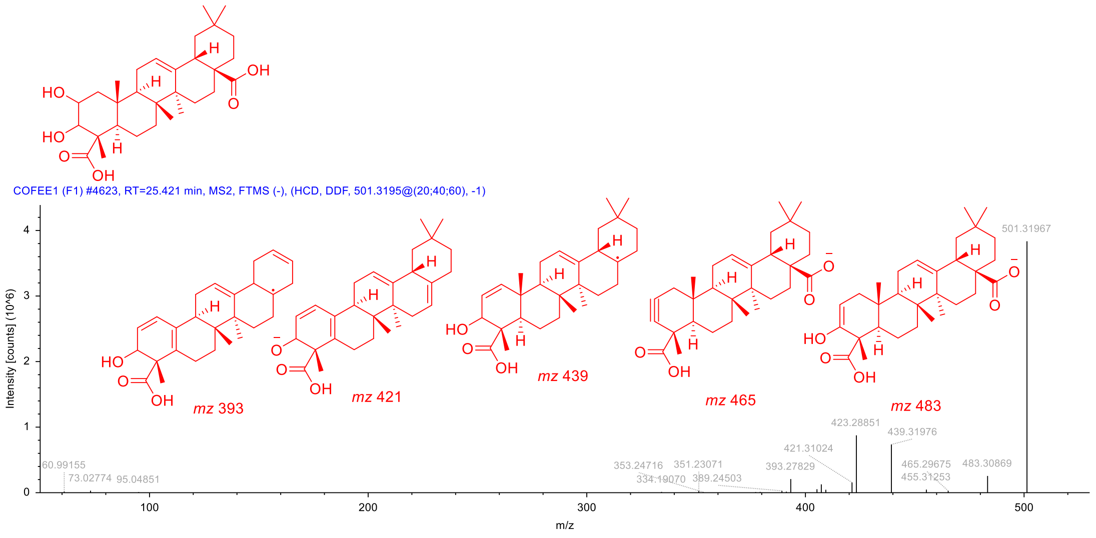

[56] Echinocystic acid (Han., et al., 2015)

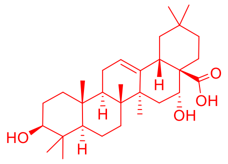

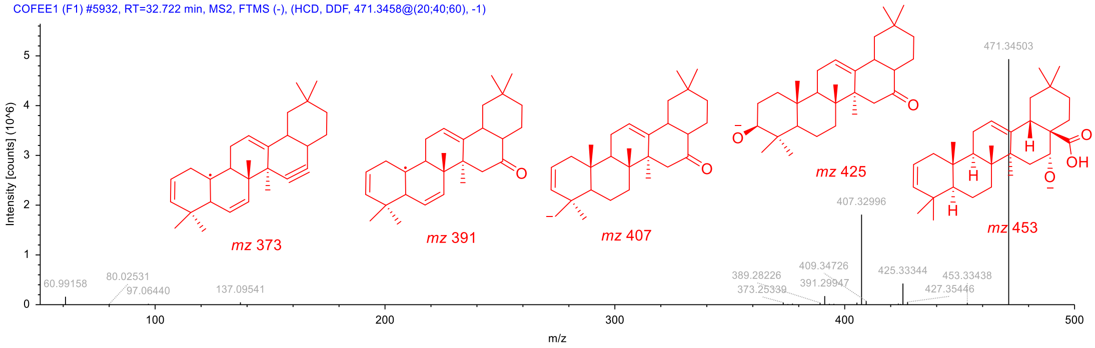

[57] 18β-Glycyrrhetintic acid (Musharraf et al., 2013)

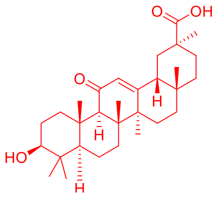

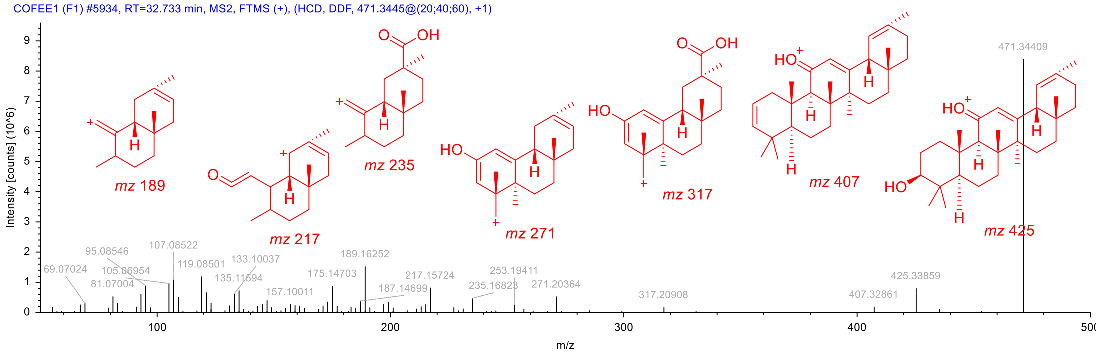

[58] Quillaic acid (Ferreira et al., 2018)

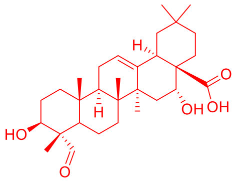

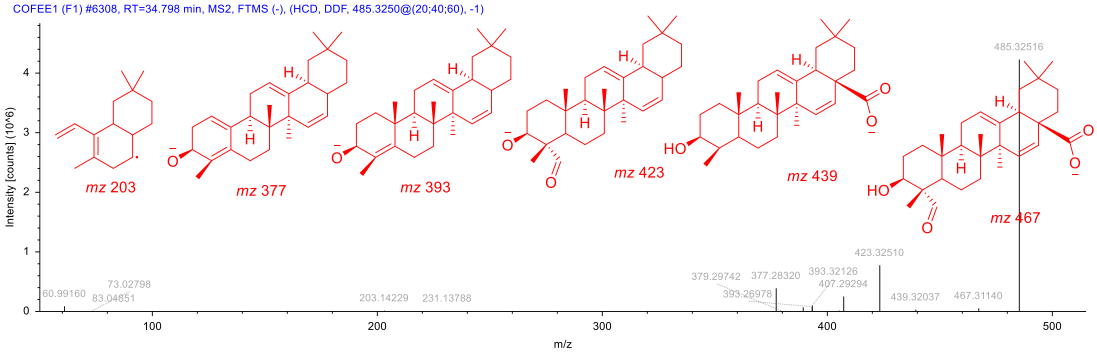

[59] Maslinic acid (Ayatollahi et al., 2011)

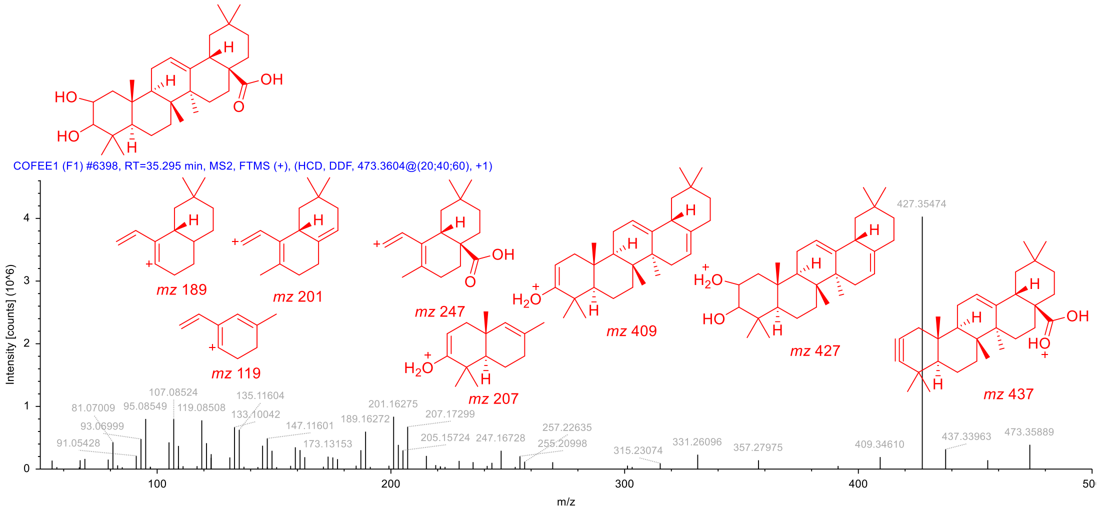

[60] Bayogenin

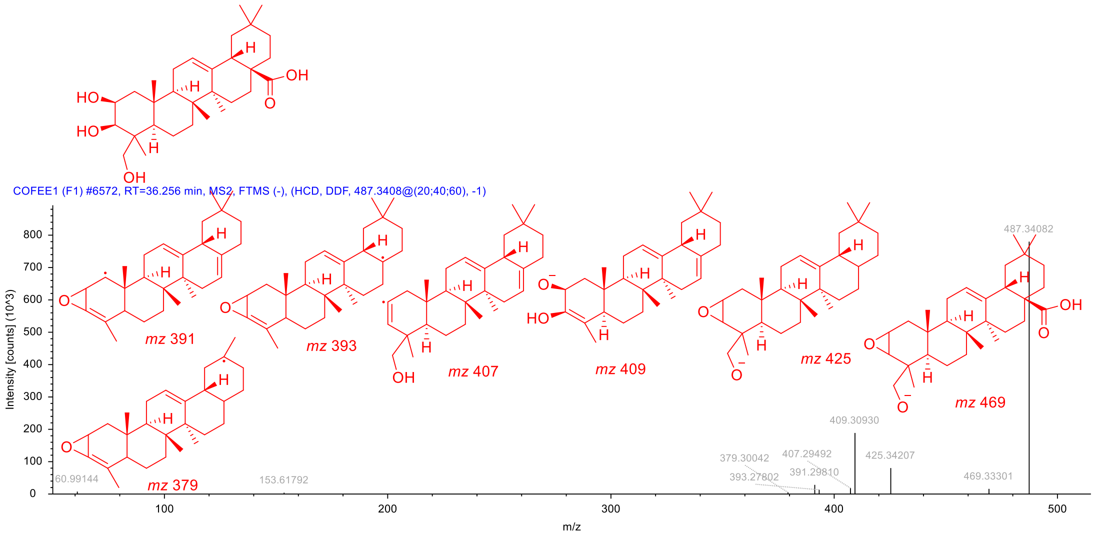

[61] Acetyl-11-keto-*β*-boswellic acid

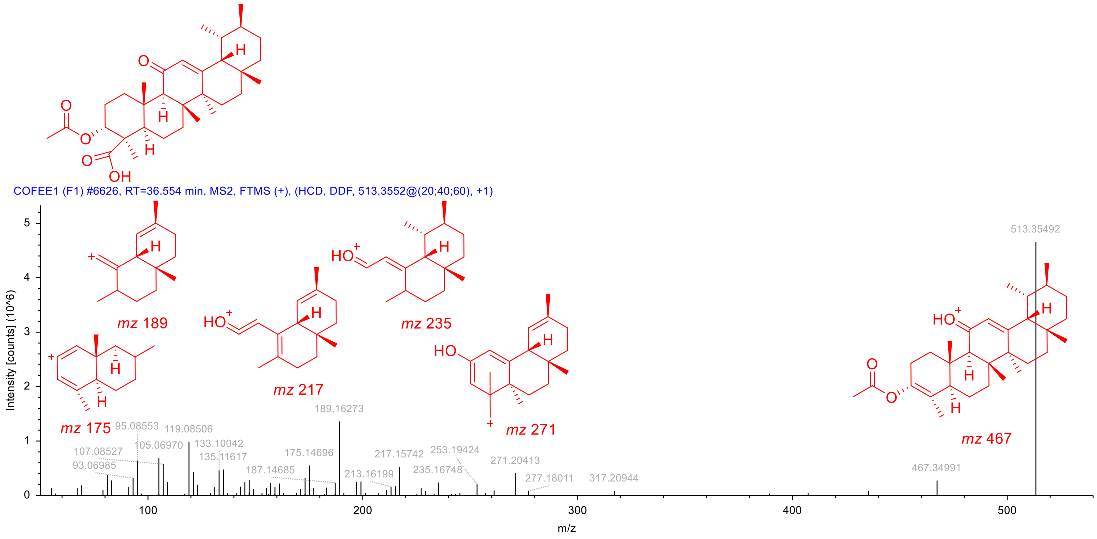

[62] α-Linolenic acid

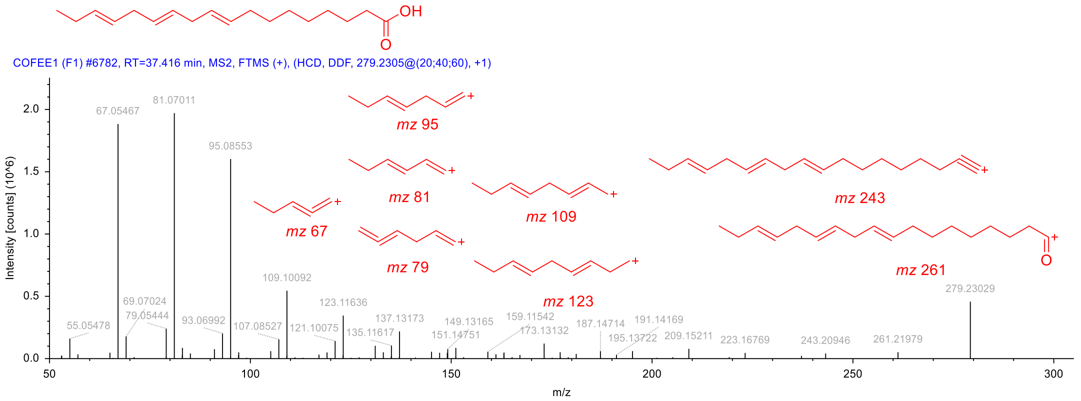

[63] 3-*O*-Acetyl-16α-hydroxytrametenolic acid (Jin et al., 2019)

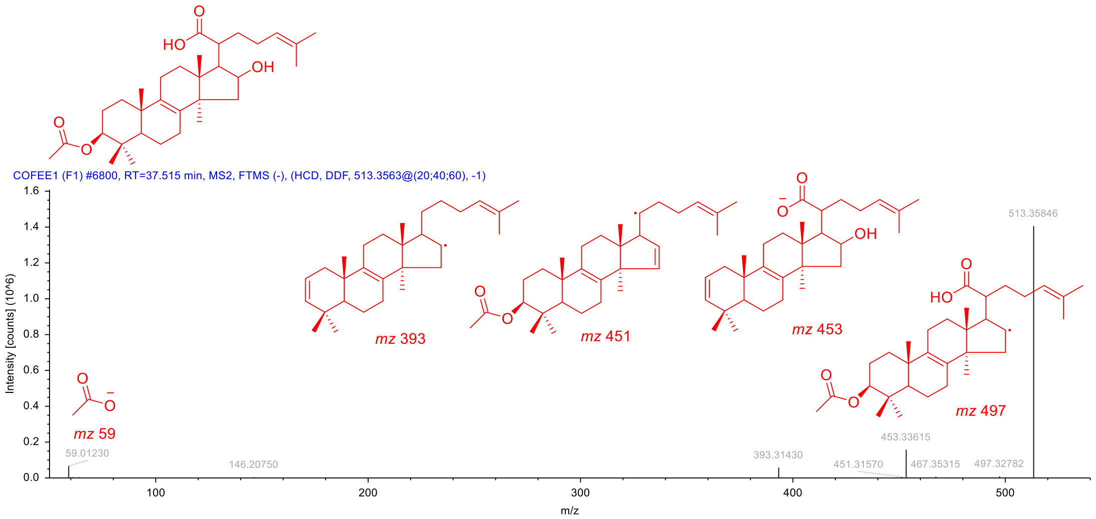

[64] Ursolic acid (Novotny et al., 2003; Ayatollahi et al., 2011)

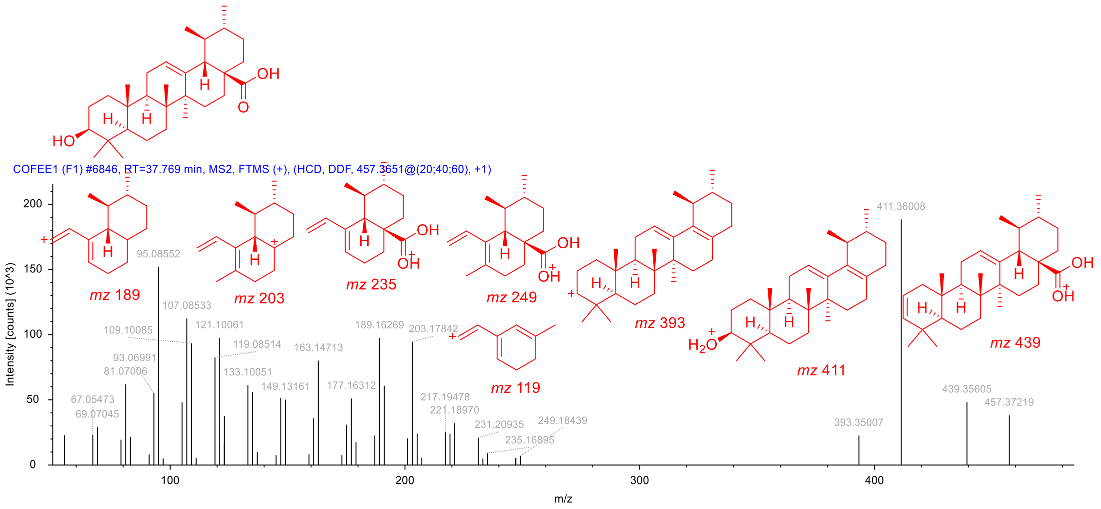

[65] Oleanonic acid (Ayatollahi et al., 2011)

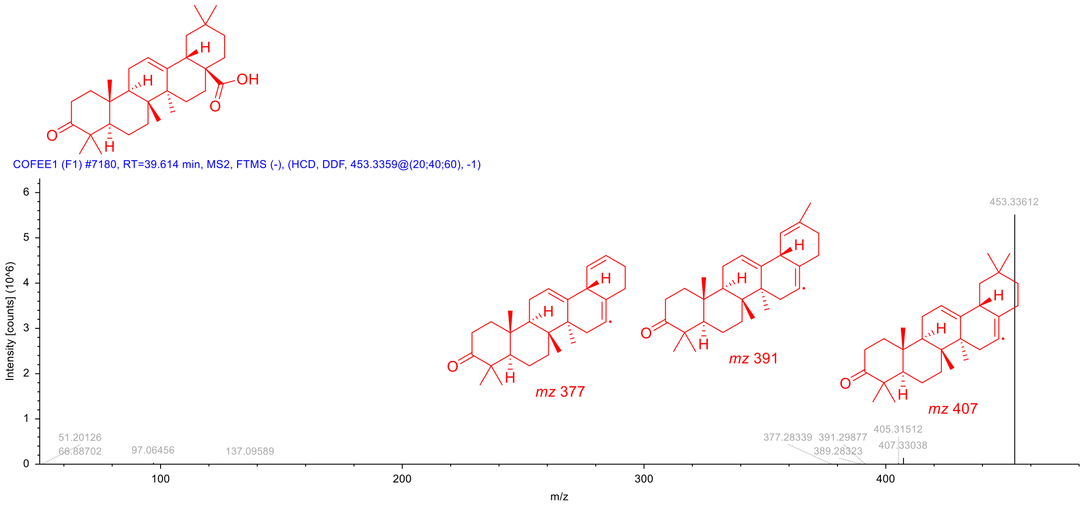

[66] Lupenone (Heinzen et al., 1996)

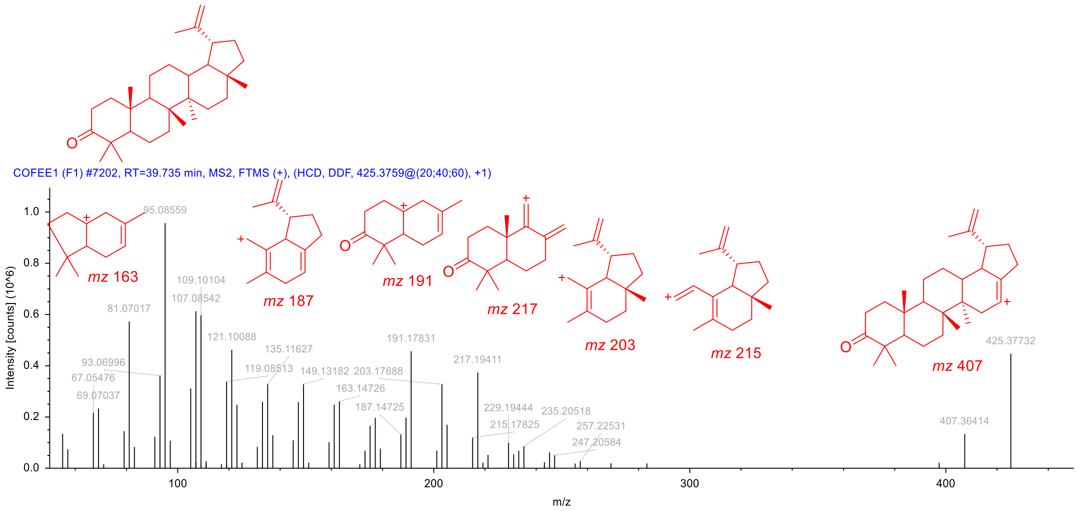

[67] Roburic acid

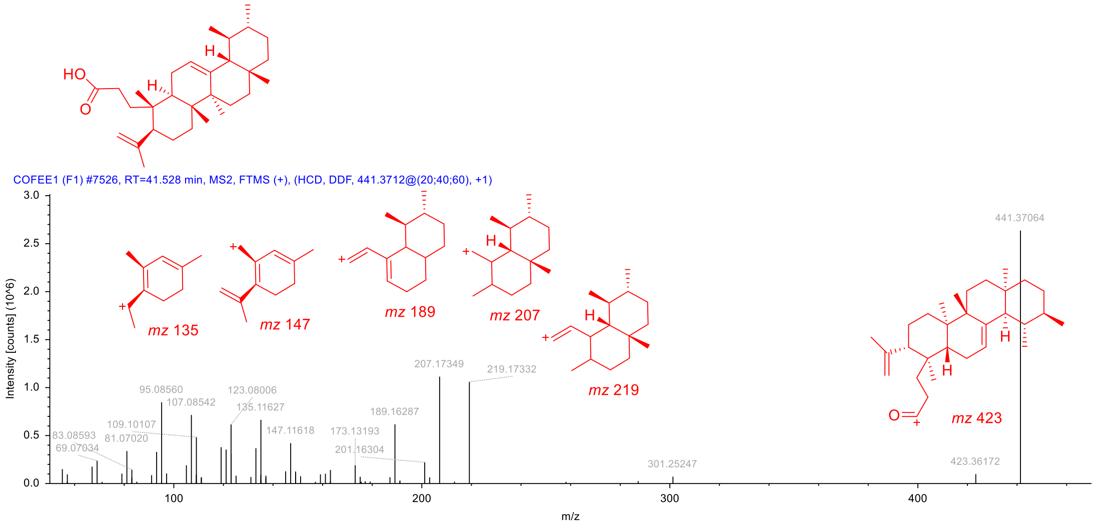

[68] α-Boswellic acid

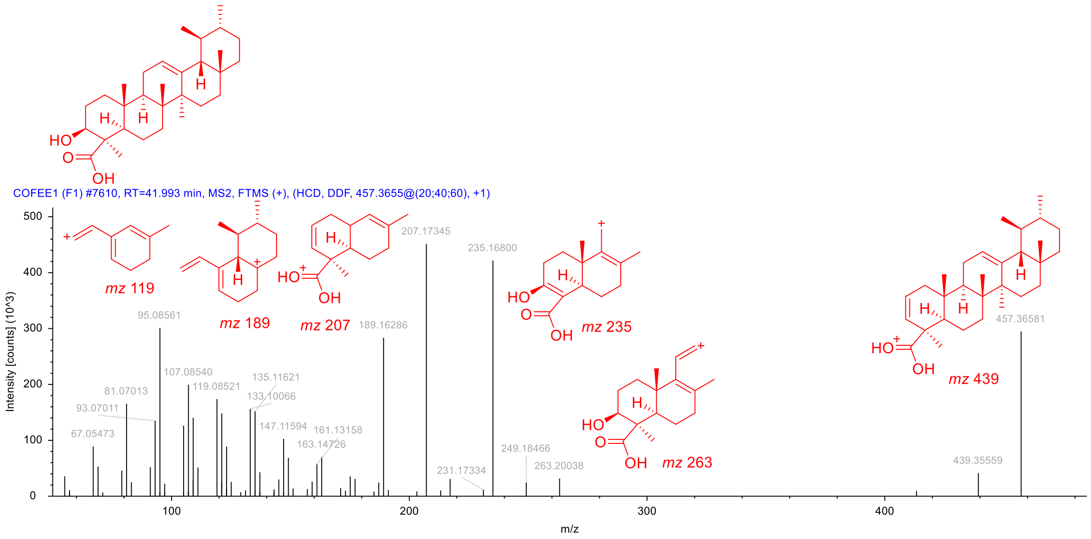

[69] β-Elemonic acid (Badria et al., 2003)

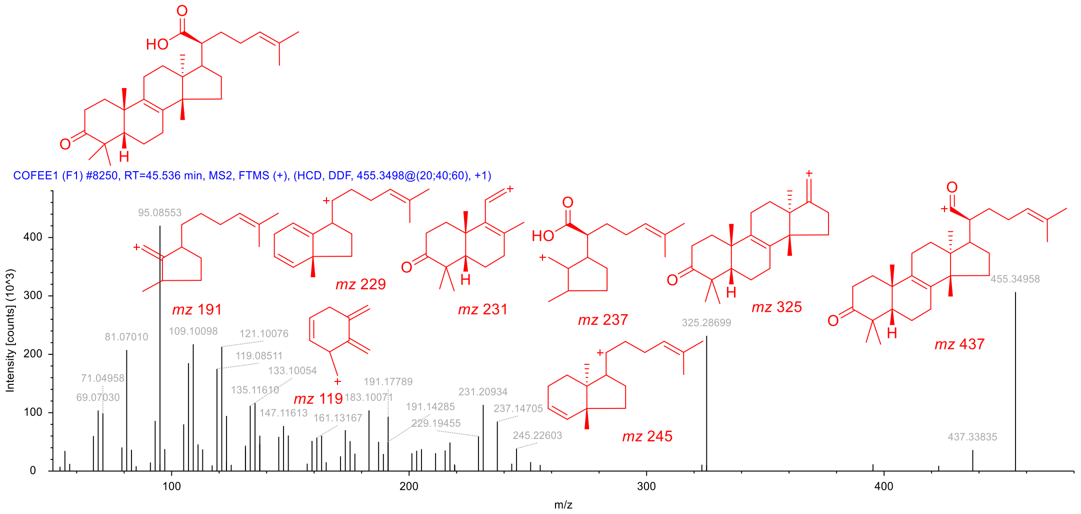

[70] Ethylparaben

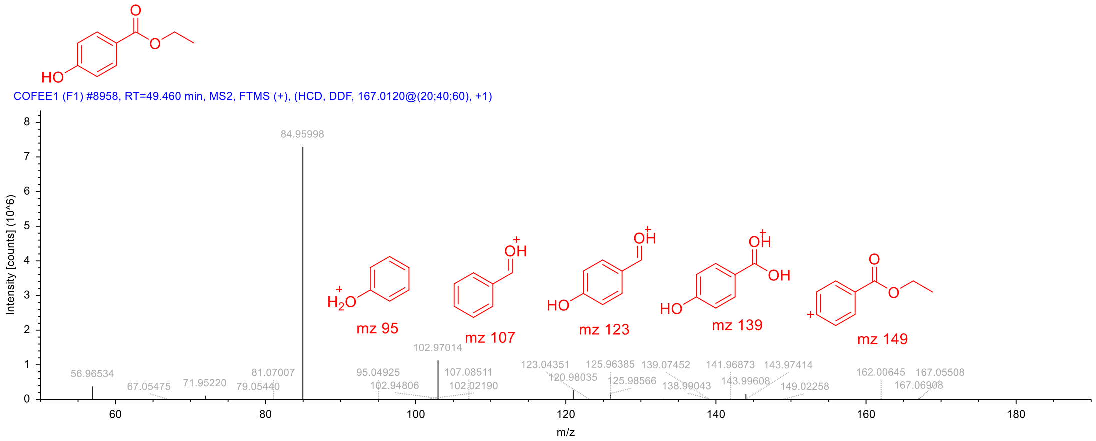

REFERENCES

Abu-Reidah, I. M., Contreras, M. M., Arráez-Román, D., Segura-Carretero, A., and Fernández-Gutiérrez, A.

(2013). Reversed-phase ultra-high-performance liquid chromatography coupled to electrospray ionization-quadrupole-time-of-flight mass spectrometry as a powerful tool for metabolic profiling of vegetables: *Lactuca sativa* as an example of its application. *J. Chromatogr. A* 1313, 212–227. doi: 10.1016/j.chroma.2013.07.020

Ayatollahi, A. M., Ghanadian, M., Afsharypour, S., Abdella, O. M., Mirzai, M., and Askari, G. (2011). Pentacyclic Triterpenes in *Euphorbia microsciadia* with Their T-cell Proliferation Activity. *Iran. J. Pharm. Res.* 10, 287–294. doi: 10.22037/ijpr.2011.963

Badria, F. A., Mikhaeil, B. R., Maatooq, G. T., and Amer, M. M. A. (2003). Immunomodulatory triterpenoids from the oleogum resin of *Boswellia carterii* Birdwood. *Z. Naturforsch. C. J. Biosci.* 58, 505–516. doi: 10.1515/znc-2003-7-811

Biazzi, E., Carelli, M., Tava, A., Abbruscato, P., Losini, I., Avato, P., et al. (2015). CYP72A67 Catalyzes a Key Oxidative Step in Medicago truncatula Hemolytic Saponin Biosynthesis. *Mol. Plant.* 8, 1493-1506. doi: 10.1016/j.molp.2015.06.003

Callemien, D., and Collin, S. (2008). Use of RP-HPLC-ESI(–)-MS/MS to Differentiate Various Proanthocyanidin Isomers in Lager Beer Extracts. *J. Am. Soc. Brew. Chem.* 66, 109–115. doi: 10.1094/ASBCJ-2008-0215-01

Castro, C. B., Luz, L. R., Guedes, J. A. C., Porto, D. D., Silva, M. F. S., Silva, G. S., et al. (2020). Metabolomics-Based Discovery of Biomarkers with Cytotoxic Potential in Extracts of *Myracrodruon urundeuva*. *J. Braz. Chem. Soc.* 31, 775–787. doi: 10.21577/0103-5053.20190242

Chuang, L., Liu, S. Y., Biedermann, D., and Franke, J. (2022). Identification of early quassinoid biosynthesis in the invasive tree of heaven (*Ailanthus altissima*) confirms evolutionary origin from protolimonoids. *Front. Plant Sci.* 13, 958138. doi: 10.3389/fpls.2022.958138

Concannon, S., Ramachandran, V. N., and Smyth, W. F. (2000). A study of the electrospray ionisation of selected coumarin derivatives and their subsequent fragmentation using an ion trap mass spectrometer. *Rapid Commun. Mass Spectrom.* 14, 1157–1166. doi: 10.1002/1097-0231(20000730)14:14<1157::AID-RCM4>3.0.CO;2-V

da Costa, M. F., Galaverna, R. S., Pudenzi, M. A., Ruiz, A. L. T. G., De Carvalho, J. E., Eberlin, M. N., et al. (2016). Profiles of phenolic compounds by FT-ICR MS and antioxidative and antiproliferative activities of Stryphnodendron obovatum Benth leaf extracts. *Anal. methods* 8, 6056–6063. doi: 10.1039/C6AY01272H

de Brito, J. A. G., Pinto, L. D. S., Chaves, C. F., Ribeiro da Silva, A. J., Silva, M. F. D. G. F. D., and Cotinguiba, F. (2021). Chemophenetic Significance of *Anomalocalyx uleanus* Metabolites Are Revealed by Dereplication Using Molecular Networking Tools. *Molecules* 26, 925. doi: 10.3390/molecules26040925

Deshpande, S., Matei, M. F., Jaiswal, R., Bassil, B. S., Kortz, U., and Kuhnert, N., (2016). Synthesis, structure, and tandem mass spectrometric characterization of the diastereomers of quinic acid. *J. Agric. Food Chem.* 64, 7298–7306.

Ding, F. J., Liu, J. T., Du, R. K., Yu, Q. H., Gong, L. L., Jiang, H. Q., et al. (2019). Qualitative and Quantitative Analysis for the Chemical Constituents of *Tetrastigma hemsleyanum* Diels et Gilg Using Ultra-High Performance Liquid Chromatography/Hybrid Quadrupole-Orbitrap Mass Spectrometry and Preliminary Screening for Anti-Influenza Virus Components. *Evid Based Complement. Alternat. Med*. 18, 9414926. doi: 10.1155/2019/9414926

Dubber, M. J., Sewram, V., Mshicileli, N., Shephard, G. S., and Kanfer, I. (2005). The simultaneous determination of selected flavonol glycosides and aglycones in *Ginkgo biloba* oral dosage forms by high-performance liquid chromatography–electrospray ionisation–mass spectrometry. *J. Pharm. Biomed. Anal.* 37, 723–731. doi: 10.1016/j.jpba.2004.11.052

Dugé de Bernonville, T., Guyot, S., Paulin, J. P., Gaucher, M., Loufrani, L., Henrion, D., et al. (2010). Dihydrochalcones: Implication in resistance to oxidative stress and bioactivities against advanced glycation

end-products and vasoconstriction. *Phytochemistry*, 71, 443–452. doi: 10.1016/j.phytochem.2009.11.004

Enomoto, H., and Nirasawa, T. (2020). Localization of Flavan-3-ol Species in Peanut Testa by Mass Spectrometry Imaging. *Molecules* 25, 2373. doi: 10.3390/molecules25102373

Fabre, N., Rustan, I., de Hoffmann, E., and Quetin-Leclercq, J. (2001). Determination of Flavone, Flavonol, and Flavanone Aglycones by Negative Ion Liquid Chromatography Electrospray Ion Trap Mass Spectrometry. *J. Am. Soc. Mass Spectrom.* 12, 707–715. doi: 10.1016/S1044-0305(01)00226-4

Ferreira, J. P. A., Miranda, I., and Pereira, H. (2018). Chemical composition of lipophilic extractives from six *Eucalyptus* barks. *Wood Sci. Technol*. 52, 1685–1699. doi: 10.1007/s00226-018-1054-6

Flamini, R., Vedova, A. D., Cancian, D., Panighel, A., and De Rosso, M. (2007). GC/MS−positive ion chemical ionization and MS/MS study of volatile benzene compounds in five different woods used in barrel making. *J. Mass Spectrom.* 42, 641–646. doi: 10.1002/jms.1193

Fortier, A. M., Asselin, E., and Cadrin, M. (2011). Functional specificity of Akt isoforms in cancer progression. *Biomol. Concepts* 2, 1–11. doi: 10.1515/bmc.2011.003

Han, L. F., Liu, E. W., Kojo, A., Zhao, J., Li, W., Zhang, Y., et al. (2015). Qualitative and quantitative analysis of *Eclipta prostrata* L. by LC/MS. 2015, 980890. *The Scientific World Journal*(无简称) doi: 10.1155/2015/980890

Han, Z. Y., Hu, E. M., Zhang, L. X., Cao, F., Chen, J. Y. Zhou, F. J., et al. (2022). Analysis on Chemical Components for the Leaves of the Miao Medicine *Toricellia Angulata* Oliv. Var. *Intermedia*(Harms) Hu by HPLC-HESI-HRMS. *Chin. J. Mod. Appl. Pharm*. 39, 1721–1730.

He, Y. J., Zhou, Y., Qin, Y., Zhou, Z. S., Zhu, M., Zhu, Y. Y., et al. (2021). Development of a LC-HRMS based approach to boost structural annotation of isomeric citrus flavanones. *Phytochem. Anal*. 32, 749–756. doi: 10.1002/pca.3020

Heger, T., Zatloukal, M., Kubala, M., Strnad, M., and Gruz, J. (2022). Procyanidin C1 from *Viola odorata* L. inhibits Na^+, K^+-ATPase. *Sci. Rep.* 12, 7011. doi: 10.1038/s41598-022-11086-y

Heinzen, H., de Vries, J. X., Moyna, P., Remberg, G., Martinez, R., and Tietze, L. F. (1996). Mass Spectrometry of Labelled Triterpenoids: Thermospray and Electron Impact Ionization Analysis. *Phytochem. Analysis* 7, 237–244. doi: 10.1002/(SICI)1099-1565(199609)7:5<237::AID-PCA310>3.0.CO;2-M

Ji, L., Shi, W. J., Li, Y. L., He, J., Xu, G., Qin, M., et al. (2022). Systematic Identification, Fragmentation Pattern, And Metabolic Pathways of Hyperoside in Rat Plasma, Urine, And Feces by UPLC-Q-Exactive Orbitrap MS. *J. Anal. Methods Chem.* 2022, 2623018. doi: 10.1155/2022/2623018

Jia, X. Y., Yang, Y., Wang, Q. Q., Tian, Y. F., Hong, Y., Tian, M. Y., et al. (2023). Phytochemical composition, antioxidant, anti-tyrosinase, anti-cholinesterase, and anti-inflammatory activities of *Rhynchanthus beesianus* rhizome extracts. *Arab. J. Chem.*16, 104952. doi: 10.1016/j.arabjc.2023.104952

Jiang, C., Arthur, C. J., and Gates, P. J. (2020). A computational and experimental study of the fragmentation of L-leucine, L-isoleucine and L-allo-isoleucine under collision-induced dissociation tandem mass spectrometry. *Analyst* 145, 6632–6638. doi: 10.1039/d0an00778a

Jiao, Q. S., Xu, L. L., Jiang, L. J., Jiang, Y. Y., Zhang, J. Y., and Liu, B. (2020). Metabolism study of hesperetin and hesperidin in rats by UHPLC-LTQ-Orbitrap MS^n. *Xenobiotica* 50, 1311–1322. doi: 10.1080/00498254.2019.1567956

Jin, J., Zhou, R. R., Xie, J., Ye, H. X., Liang, X. J., Zhong, C., et al. (2019). Insights into Triterpene Acids in Fermented Mycelia of Edible Fungus *Poria cocos* by a Comparative Study. *Molecules* 24, 1331. doi: 10.3390/molecules24071331

Justesen U. (2000). Negative atmospheric pressure chemical ionisation low-energy collision activation mass spectrometry for the characterisation of flavonoids in extracts of fresh herbs. *J. Chromatogr. A* 902, 369–379.

doi: 10.1016/s0021-9673(00)00861-x

Kutney, J. P., Engendorf, G., Inaba, T., and Dreyer, D. L., (1971). MASS SPECTRAL FRAGMENTATION STUDIES IN MONOMERIC AND DEMERIC COUMARINS*. *Org. Mass Spectrom*. 5, 249–263. doi: 10.1002/oms.1210050303

Li, Z. Q., Zhang, H. N., He, M. Z., Feng, Y. L., Yang, S. L., and Zhong, G. Y. (2021). Study on the chemical constituents by UHPLC-Q-TOF/MS and antioxidant activities in vitro of *Rhodiola fastigiata*. *Lishizhen Med. Mater. Med. Res*. 32, 2361–2365. doi: 10.3969 /j.issn.1008-0805.2021.10.14

Li, Y. J., He, F. Q., Zhao, H. H., Li, Y., and Chen, J. (2022). Screening and identification of acetylcholinesterase inhibitors from *Terminalia chebula* fruits by immobilized enzyme on cellulose filter paper coupled with ultra-performance liquid chromatography-quadrupole time-of-flight mass spectrometry and molecular docking. *J. Chromatogr. A* 1663, 462784. doi: 10.1016/j.chroma.2021.462784

Liang, G. P., Wang, W., Wu, J. L., Liu, J., and Yang, J. (2022). Rapid identification on chemical compounds in the extracts from viburnum foetidum fruits with anti-tumor activities based on UPLC-ESI-HRMS^n. *J. Shaanxi Univ. Sci. Technol.* 40, 70–77. doi: 10.19481/j.cnki.issn2096-398x.2022.06.008

Lu, Y. C., Wang, X., Wu, Y., Wang, Z. Y., Zhou, N., Li, J. J., et al. (2022). Chemical characterization of the antioxidant and α-glucosidase inhibitory active fraction of *Malus transitoria* leaves. *Food Chem*. 386, 132863. doi: 10.1016/j.foodchem.2022.132863

Ma, Y. H., Tian, T. T., Xie, W. W., Jin., Y. R., Xu, H. J., Zhang, L. T., et al. (2016). Major phenolic acids in *Usneae Filum* by UHPLC-Triple-TOF-MS. *Chin. Tradit. Herbal Drugs* 3, 392–400. doi: 10.7501/j.issn.0253-2670.2016.03.007

March, R. E., Miao, X. S., (2004). A fragmentation study of kaempferol using electrospray quadrupole time-of-flight mass spectrometry at high mass resolution. *Int. J. Mass Spectrom.* 231, 157–167. doi: 10.1016/j.ijms.2003.10.008

Menicatti, M., Pallecchi, M., Ricciutelli, M., Galarini, R., Moretti, S., Sagratini, G., et al. (2020). Determination of coeluted isomers in wine samples by application of MS/MS deconvolution analysis. *J. Mass Spectrom*. 55, e4607. doi: 10.1002/jms.4607

Moss, R., Mao, Q. Y., Taylor, D., and Saucier, C. (2013). Investigation of monomeric and oligomeric wine stilbenoids in red wines by ultra‐high‐performance liquid chromatography/electrospray ionization quadrupole time‐of‐flight mass spectrometry. *Rapid Commun. Mass Spectrom*. 27, 1815–1827. doi: 10.1002/rcm.6636

Musharraf, S. G., Kanwal, N., and Arfeen, Q. U. (2013). Stress degradation studies and stability-indicating TLC-densitometric method of glycyrrhetic acid. *Chem. Cent. J.* 7, 1–10. doi: 10.1186/1752-153X-7-9

Novotny, L., Abdel-Hamid, M. E., Hamza, H., Masterova, I., and Grancai, D. (2003). Development of LC–MS method for determination of ursolic acid: application to the analysis of ursolic acid in *Staphylea holocarpa* Hemsl. *J. Pharm. Biomed. Anal.* 31, 961–968. doi: 10.1016/s0731-7085(02)00706-9

Oliveros, J. C. (2007). VENNY. An interactive tool for comparing lists with Venn Diagrams. *http://bioinfogp. cnb. csic. es/tools/venny/index. html*.

Opitz, J., Hashmi, A S. K, Miehlich, B., Wölfle, M. (2020). Electron-induced ionization of undeuterated and deuterated benzoic acid isopropyl esters and nicotinic acid isopropyl esters: Some implications for the mechanism of the McLafferty rearrangement. *Eur. J. Mass Spectrom.* 26, 3–24. doi: 10.1177/1469066719857994

Peeters, L., Vervliet, P., Foubert, K., Hermans, N., Pieters, L., and Covaci, A. (2020). A comparative study on the *in vitro* biotransformation of medicagenic acid using human liver microsomes and S9 fractions. *Chem. Biol. Interact.* 328, 109192. doi: 10.1016/j.cbi.2020.109192

Scheubert, K., Hufsky, F., and Böcker, S. (2013). Computational mass spectrometry for small molecules. *J. Cheminform.* 5, 1–24. doi: 10.1186/1758-2946-5-12

Shahzad, M. N., Ahmad, S., Tousif, M. I., Ahmad, I., Rao, H., Ahmad, B., et al. (2022). Profiling of phytochemicals from aerial parts of *Terminalia neotaliala* using LC-ESI-MS^2 and determination of antioxidant and enzyme inhibition activities. *PloS one* 17, e0266094. doi: 10.1371/journal.pone.0266094

Shi, J. C., Gao, X. X., Zhang, A. R., Qin, X. M., and Du, G. H. (2022). Characterization of multiple chemical components of GuiLingJi by UHPLC-MS and ^{1}H NMR analysis. *J. Pharm. Anal.* 12, 460–469. doi: 10.1016/j.jpha.2021.09.013

Sriseadka, T., Wongpornchai, S., and Rayanakorn, M. (2012). Quantification of flavonoids in black rice by liquid chromatography-negative electrospray ionization tandem mass spectrometry. *J. Agric. Food Chem.* 60, 11723–11732. doi: 10.1021/jf303204s

Stražić, D., Benković, T., Gembarovski, D., Kontrec, D., and Galić, N. (2014). Comprehensive ESI-MS and MS/MS analysis of aromatic hydrazones derived from nicotinic acid hydrazide. *Int. J. Mass Spectrom*. 371, 54–64. doi: 10.1016/j.ijms.2014.07.036

Su, H. N. (2023). Quality Evaluation of Yixuean Granule’s Chinese medicinal material Blumeae Ripariae Herba. [master's thesis]. [Chengdu (Sichuan Province)]: Southwest Minzu University.

Sun, L. Q., Tang, Z. J., Wang, M. X., Shi, J., Lin, Y. J., Sun, T. F., et al. (2022). Exploration of Antimicrobial Ingredients in *Psoralea corylifolia* L. Seed and Related Mechanism against Methicillin-Resistant *Staphylococcus aureus*. *Molecules* 27, 6952. doi: 10.3390/molecules27206952

Sun, X. Y., Xue, S. J., Cui, Y. X., Li, M., Chen, S. Q., Yue, J. Y., et al. (2023). Characterization and identification of chemical constituents in Corni Fructus and effect of storage using UHPLC-LTQ-Orbitrap-MS. *Food Res. Int.* 164, 112330. doi: 10.1016/j.foodres.2022.112330

Syahbirin, G., Chahyaningtias, A. L., Radita, R., and Ilmiawati, A. (2020). Secondary metabolites of Temu *Putih* (*Curcuma zedoaria*) rhizome. *AIP Conf. Proc.* 2243, 030026. doi: 10.1063/5.0006477

Tao, J. (2021). Study on Chemical Substances and Pharmacodynamics of Malt Alkaloids Based on UPLC-Q-TOF-MS/MS and Network Pharmacology. [master's thesis]. [Wuhan (Hubei Province)]: Hubei University of Traditional Chinese Medicine.

UniProt Consortium. (2023). UniProt: the Universal Protein Knowledgebase in 2023. *Nucleic Acids Res*. 51, D523–D531. doi: 10.1093/nar/gkac1052

Wang, Y. (2015). Studies on analysis of flavonoids of flowers, stems and leaves of *Abelmoschus manihot* (L.) Medicus. [master's thesis]. [Beijin]: Beijing University of Chinese Medicine).

Wang, Z. X., Liu, J. Y., Zhong, X. J., Li, J. J., Wang, X., Ji, L. L., et al. (2019). Rapid Characterization of Chemical Components in Edible Mushroom *Sparassis crispa* by UPLC-Orbitrap MS Analysis and Potential Inhibitory Effects on Allergic Rhinitis. *Molecules* 24, 3014. doi: 10.3390/molecules24163014

Wang, X., Zhong, X. J., Zhou, N., Cai, N., Xu, J. H., Wang, Q. B., et al. (2020). Rapid Characterizaiton of Chemical Constituents of the Tubers of *Gymnadenia conopsea* by UPLC-Orbitrap-MS/MS Analysis. *Molecules* 25, 898. doi: 10.3390/molecules25040898

Wang, K. H., Tian, J. Y., Li, Y. S., Liu, M. S., Chao, Y. X., Cai, Y., et al. (2021). Identification of Components in Citri Sarcodactylis Fructus from Different Origins via UPLC-Q-Exactive Orbitrap/MS. *ACS omega* 6, 17045–17057. doi: 10.1021/acsomega.1c02124

Wee, P., and Wang, Z., X. (2017). Epidermal Growth Factor Receptor Cell Proliferation Signaling Pathways. *Cancers* 9, 52. doi: 10.3390/cancers9050052

Wei, Y. L., Shu, P., Hong, J. L., and Qin, M. J. (2012). Qualitative and quantitative evaluation of phenolic compounds in Iris dichotoma Pall. *Phytochem. Anal.* 23, 197–207. doi: 10.1002/pca.1343

Weinert, C. H., Wiese, S., Rawel, H. M., Esatbeyoglu, T., Winterhalter, P., Homann, T., et al. (2012). Methylation of Catechins and Procyanidins by Rat and Human Catechol-*O*-Methyltransferase: Metabolite Profiling and Molecular Modeling Studies. *Drug Metab. Dispos.* 40, 353–359. doi: 10.1124/dmd.111.041871

Wojnicz, A., Ortiz, J. A., Casas, A. I., Freitas, A. E., López, M. G., and Ruiz-Nuño, A. (2016). Data supporting the rat brain sample preparation and validation assays for simultaneous determination of 8 neurotransmitters and their metabolites using liquid chromatography-tandem mass spectrometry. *Data Brief* 7, 714–720. doi: 10.1016/j.dib.2016.03.025

Wolf, S., Schmidt, S., Müller-Hannemann, M., and Neumann, S. (2010). In silico fragmentation for computer assisted identification of metabolite mass spectra. *BMC Bioinformatics* 11, 148. doi: 10.1186/1471-2105-11-148

Wu, N., Aimaitijiang, T., Chang J. M., and Li, G. R. (2023). Screening of anti-asthmatic active extracts from *Sorbus tianschanica* Rupr. and component analysis. *Cent. South Pharm.*, 4, 863–869. doi: 10.7539/j.issn.1672-2981.2023.04.005

Xiao, Y., Hu, Z. Z., Yin, Z. T., Zhou, Y. M., Liu, T. Y., Zhou, X. L., et al. (2017a). Profiling and Distribution of Metabolites of Procyanidin B2 in Mice by UPLC-DAD-ESI-IT-TOF-MS^n Technique. *Front. Pharmacol. 8*, 231. doi: 10.3389/fphar.2017.00231

Xiao, X., Xu, L. J., Hu, H. G., Yang, Y. J., Zhang, X. Y., Peng, Y., et al. (2017b). DPPH Radical Scavenging and Postprandial Hyperglycemia Inhibition Activities and Flavonoid Composition Analysis of Hawk Tea by UPLC-DAD and UPLC-Q/TOF MS^E. *Molecules* 22, 1622. doi: 10.3390/molecules22101622

Xu, Y., Liang, P. L., Chen, X. L., Gong, M. J., Zhang, L., Qiu, X. H., et al. (2021). The Impact of *Citrus*-Tea Cofermentation Process on Chemical Composition and Contents of Pu-Erh Tea: An Integrated Metabolomics Study. *Front. Nutr.* 8, 737539. doi: 10.3389/fnut.2021.737539

Yisimayili, Z., Guo, X. Z., Liu, H., Xu, Z., Abdulla, R., Akber, Aisa. H, et al. (2019). Metabolic profiling analysis of corilagin *in vivo* and *in vitro* using high-performance liquid chromatography quadrupole time-of-flight mass spectrometry. *J. Pharm. Biomed. Anal*. 165, 251–260. doi:10.1016/j.jpba.2018.12.013

Youssef, M. E., Cavalu, S., Hasan, A. M., Yahya, G., Abd-Eldayem, M. A., and Saber, S. (2023). Role of Ganetespib, an HSP90 Inhibitor, in Cancer Therapy: From Molecular Mechanisms to Clinical Practice. *Int. J. Mol. Sci.* 24, 5014. doi: 10.3390/ijms24055014

Zhao, J. F., Shoeib, T., Siu, K. W. M., and Hopkinson, A. C. (2006). The fragmentation of protonated tyrosine and iodotyrosines: The effect of substituents on the losses of NH_3 and of H_2O and CO. *Int. J. Mass Spectrom.* 255–256, 265–278. doi: 10.1016/j.ijms.2006.03.012

Zhang, Y. Y., Wang, Q., Qi, L. W., Qin, X. Y., and Qin, M. J. (2011). Characterization and determination of the major constituents in Belamcandae Rhizoma by HPLC-DAD-ESI-MS^n. *J. Pharm. Biomed. Anal.* 56, 304–314. doi: 10.1016/j.jpba.2011.05.040

Zhang, P. W., Chan, W., Ang, I. L., Wei, R., Lam, M. M. T., Lei, K. M. K., et al. (2019). Revisiting Fragmentation Reactions of Protonated α-Amino Acids by High-Resolution Electrospray Ionization Tandem Mass Spectrometry with Collision-Induced Dissociation. *Sci. Rep*. 9, 6453. doi: 10.1038/s41598-019-42777-8

Zhang, Y. Y., Liu, X. H., Gao, S. T., Qian, K., Liu, Q. S., and Yin, X. Y. (2018). Research on the neuro-protective compounds in *Terminalia chebula* Retz extracts in-vivo by UPLC–QTOF-MS. *Acta Chromatogr.* 30, 169–174. doi: 10.1556/1326.2017.00147

Zheng, W., Shi, H. Y., Wang, P., Wang, S. L., Cai, M. C. and Xu, N. (2022). Indentification of chemical constituents and blood components of Banxia Baizhu Tianma Decoction by UPLC-Q-Orbitrap-HRMS. *Chin. J. Hosp. Pharm.* 22, 2331–2339. doi: 10.13286/j.1001-5213.2022.22.04
